# Boosting Peroxide Reactivity: New Mn(II) Chelidamate Catalysts for Efficient Low-Temperature Bleaching

**DOI:** 10.1021/acsomega.6c04886

**Published:** 2026-08-07

**Authors:** Immacolata Manco, Vincenzo Langellotti, Maria E. Cucciolito, Carlo De Luca, Roberto Esposito, Matteo Lega, Massimo Melchiorre, Gabriella Pinto, Oreste Tarallo, Francesco Ruffo

**Affiliations:** † Dipartimento di Scienze Chimiche, Università di Napoli Federico II, Via Cintia 21, Napoli 80126, Italy; ‡ Consorzio Interuniversitario di Reattività Chimica e Catalisi, Via Celso Ulpiani 27, Bari 70126, Italy; § ISusChem Srl, Piazza Carità 32, Napoli 80134, Italy; ∥ Impresa Fater SpA, Via Mare Adriatico 122, Spoltore 65010, Italia

## Abstract

The development of efficient catalytic systems for low-temperature bleaching is a key challenge for reducing energy consumption in laundry processes. In this work, five manganese­(II) complexes derived from chelidamic acid were designed, synthesized, and evaluated as bleaching catalysts for the activation of hydrogen peroxide under mild and aqueous conditions. The ligand framework was functionalized by *O*-alkylation at the 4-hydroxy group with neutral, cationic, and anionic substituents to modulate hydrophobicity and charge distribution. All complexes were tested in morin bleaching as a benchmark reaction, exhibiting good conversions (>90%) already at 20 °C within a short reaction time (30 min). The *O*-functionalized Mn­(II) complexes significantly outperformed the free hydroxyl analogue and Mn­(II) salt precursor. Kinetic analysis revealed the presence of an induction period, followed by a *quasi*-linear regime used to determine apparent turnover frequencies (TOF), ranging from ∼80 to 180 h^–1^. The catalytic behavior was strongly influenced by the reaction medium, showing a marked dependence on bicarbonate buffer concentration, achieving optimal activity at moderate alkaline pH (≈9.3). Notably, efficient bleaching was achieved at low catalyst concentrations (μM range) and low hydrogen peroxide loadings (0.05% w/w). Furthermore, the catalytic system was also effective in the oxidation of the azo dye Sunset Yellow FCF, supporting its potential application in the wastewater treatment of degradation-resistant pollutants. These results highlight the importance of ligand design in tuning catalytic performance and support the potential application of these manganese complexes as sustainable bleaching catalysts operating under mild conditions.

## Graphical abstract

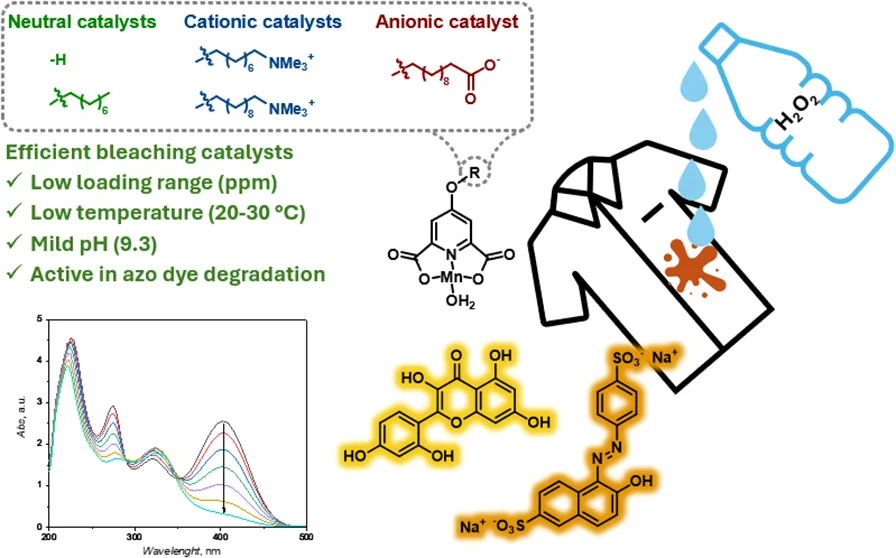

## Introduction

Developing high-performance detergent systems capable of effective cleaning at low washing temperatures is a technological challenge, as it offers the potential to reduce energy consumption, lower costs, and minimize environmental impact.^1–3^ Hydrogen peroxide-based bleaching systems are widely used in laundry detergents due to their high efficiency and environmental compatibility. Upon decomposition, this oxidant produces only water and oxygen, thereby minimizing the environmental impact of effluents. In addition, it is preferred over chlorine-based bleaches because it prevents the formation of harmful halogenated byproducts in wastewater streams.^4–6^ However, at temperatures below 40 °C, hydrogen peroxide is kinetically inert, and its bleaching performance is inefficient. A strategy to enhance the reactivity of hydrogen peroxide is to use activators. Bleaching activators are generally acylating agents that generate peracids, species that are more active than hydrogen peroxide at temperatures between 40 and 60 °C.^3,7–9^

The most widely used commercial bleaching activators include tetraacetylethylenediamine (TAED), nonanoyloxybenzenesulfonate (NOBS), sodium lauroyloxybenzenesulfonate (LOBS), and decanoyloxybenzoic acid (DOBA) (Figure 1A). These activators promote hydrogen peroxide bleaching at temperatures around 40 °C; however, this temperature is still higher than those commonly employed in many regions, where washing is often performed below 40 °C.^10^ Moreover, the use of organic bleaching activators requires stoichiometric amounts to generate peracids, which can lead to increased formulation costs and, under certain conditions, fabric and dye damage.^11^ As an alternative strategy, hydrogen peroxide can be activated through the use of transition-metal catalysts, which are capable of catalyzing substrate oxidation and enabling effective bleaching at low temperatures.^12–14^ Manganese is typically preferred for this type of activation, as it is one of the earth-abundant metals, has a good ecotoxicological profile, and is also present in different biological systems.^15^ Also, manganese is an essential trace element naturally present in aquatic environments. Nevertheless, elevated manganese concentrations have been reported as a potential emerging environmental concern because of their effects on aquatic organisms and developmental processes. In the present study, however, the catalyst was employed at concentrations below 1 ppm, which are substantially lower than those generally associated with ecotoxicological effects reported in the literature.^16^ Manganese ions act as versatile redox centers in a variety of oxidoreductases, where they enable both peroxidase-like and catalase-like reactivity. For instance, manganese catalase enzymes (MnCat) promote the disproportionation of hydrogen peroxide into molecular oxygen and water, thereby protecting cells from oxidative stress caused by an excess of reactive oxygen species (ROS).^15,17^ In contrast, manganese peroxidases (MnP) exhibit pronounced peroxidase activity in which hydrogen peroxide is used as an oxidant to convert Mn­(II) into Mn­(III). The resulting Mn­(III) species acts as a diffusible oxidant capable of oxidizing phenolic structures in lignin as well as a range of organic pollutants.^18,19^

**Figure 1:**
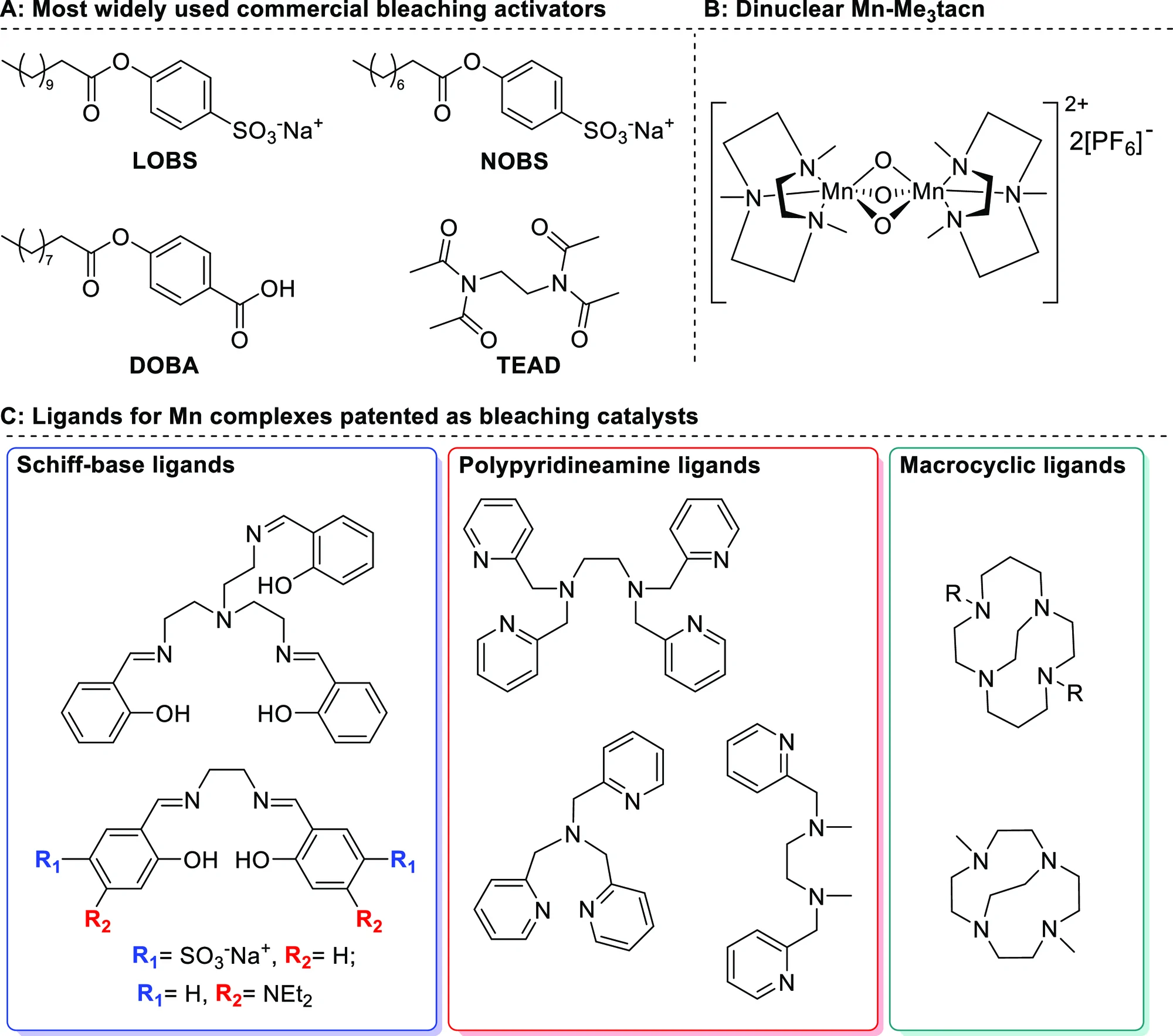
(A) Organic hydrogen peroxide activators, (B) Lewis structure of the first Mn-based complex used in hydrogen peroxide bleaching, and (C) state-of-the-art of widely used ligands for Mn­(II) complexes applied as bleaching catalysts.

Many synthetic biomimetics of catalase and peroxidase have been developed as efficient catalytic systems for the degradation of various pollutants and bleaching, which can be applied, for example, in food, dairy, and textile industries.^13,20–22^ Mn–Me_3_tacn, a dinuclear manganese catalyst containing 1,4,7-trimethyl-1,4,7-triazacyclononane (Me_3_tacn) as ligand, is an example of the first prominent manganese-based bleaching catalysts (Figure 1B) inspired by the active center of dimanganese catalase enzymes.^23,24^ This catalyst was studied in different reactions such as C–H activation,^25^ phenols and alcohols oxidation,^26,27^ olefine epoxidation,^28–30^ and also for laundry applications.^31,32^ In particular, Mn–Me_3_tacn was the first bleaching catalyst commercialized thanks to its catalytic ability to remove tea stains at 40 °C and alkaline pH (between 9 and 11); however, it was withdrawn from the market because of fabric and dye damage.^13,14,33^ However, Mn–Me_3_tacn is still applied in various machine-dishwashing products and is responsible for superior removal of tea residues.^34^

Following this idea, over the years, other manganese complexes bearing several ligands, such as Schiff base, terpyridine, cross-bridged macrocyclic, and polypyridineamine ligands (Figure 1C), were synthesized and applied as bleaching catalysts.^35–41^

Aiming to contribute to this emerging field of research, in this work, we present the synthesis and characterization of new manganese­(II) complexes derived from chelidamic acid (Figure 2) and the study of their catalytic performance in the bleaching of morin in alkaline conditions at low temperature. Chelidamic acid (4-hydroxypyridine-2,6-dicarboxylic acid) was chosen thanks to its distinctive characteristics, such as the ability to form complexes with Fe^3+^ and divalent ions, Mn^2+^, Cu^2+^, Ni^2+^, and Zn^2+^,^42^ the capability of different coordination modes,^43^ and importantly the presence of a hydroxyl group at the 4-position that enables a convenient functionalization of the ligand. In fact, in this study, alkyl substituents of variable length (C_8_–C_10_) were introduced to modulate the hydrophobicity and charge distribution of the ligand framework. The dependence of the catalytic activity on the pH, temperature, buffer concentration, and H_2_O_2_ concentration was investigated. To simplify the ligands (**L**) and complexes (**C**) labeling, the number of carbon atoms (**n**) involved in the *O*-alkyl chain is reported in the subscript (**L**_**n**_, **C**_**n**_), and the presence of an ionic chain-end moiety (**i**) in the applied conditions in the superscript (**L**_**n**_^**i**^, **C**_**n**_^**i**^). For instance, Mn-complex with undecanoic carboxylic acid as the *O*-group is reported as **C**_**11**_^**–**^ since it is expected to be in its carboxylate form in the investigated pH ranges.

**Figure 2:**
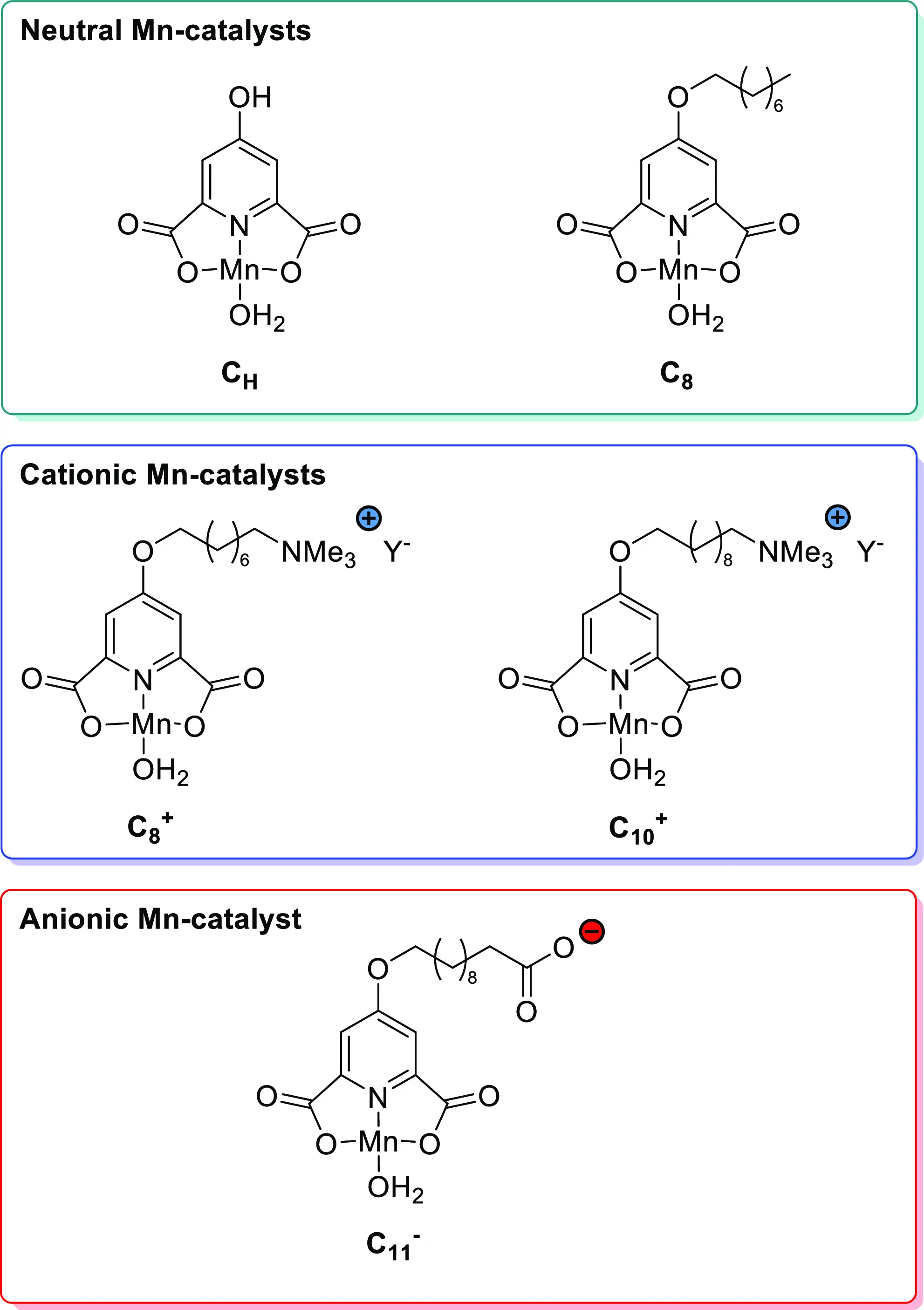
Manganese­(II) complexes derived from chelidamic acid used in this study.

## Results and Discussion

### Synthesis and Characterization

The synthesis of the ligands involved few sequential steps (Scheme 1). Initially, the carboxylic groups were protected via acid-catalyzed Fischer esterification. The hydroxyl group was then functionalized through an S_N_2 reaction using alkyl chains of different lengths (C_8_–C_10_) and terminal groups. Three end-group functionalities were examined: alkyl chains bearing a cationic trimethylammonium group (**L**_**8**_^**+**^ and **L**_**10**_^**+**^); a neutral octyl chain (**L**_**8**_); and a chain bearing a carboxylic acid moiety, expected to be in its anionic carboxylate form under applied conditions (**L**_**11**_^**–**^). Finally, the ester groups were deprotected under basic conditions to afford the desired ligand. These substituents were incorporated to fine-tune the hydrophobicity (e.g., alkyl chain length) and the expression of a charge (e.g., cationic, neutral, or anionic groups) on the ligand framework.

**Scheme 1:**
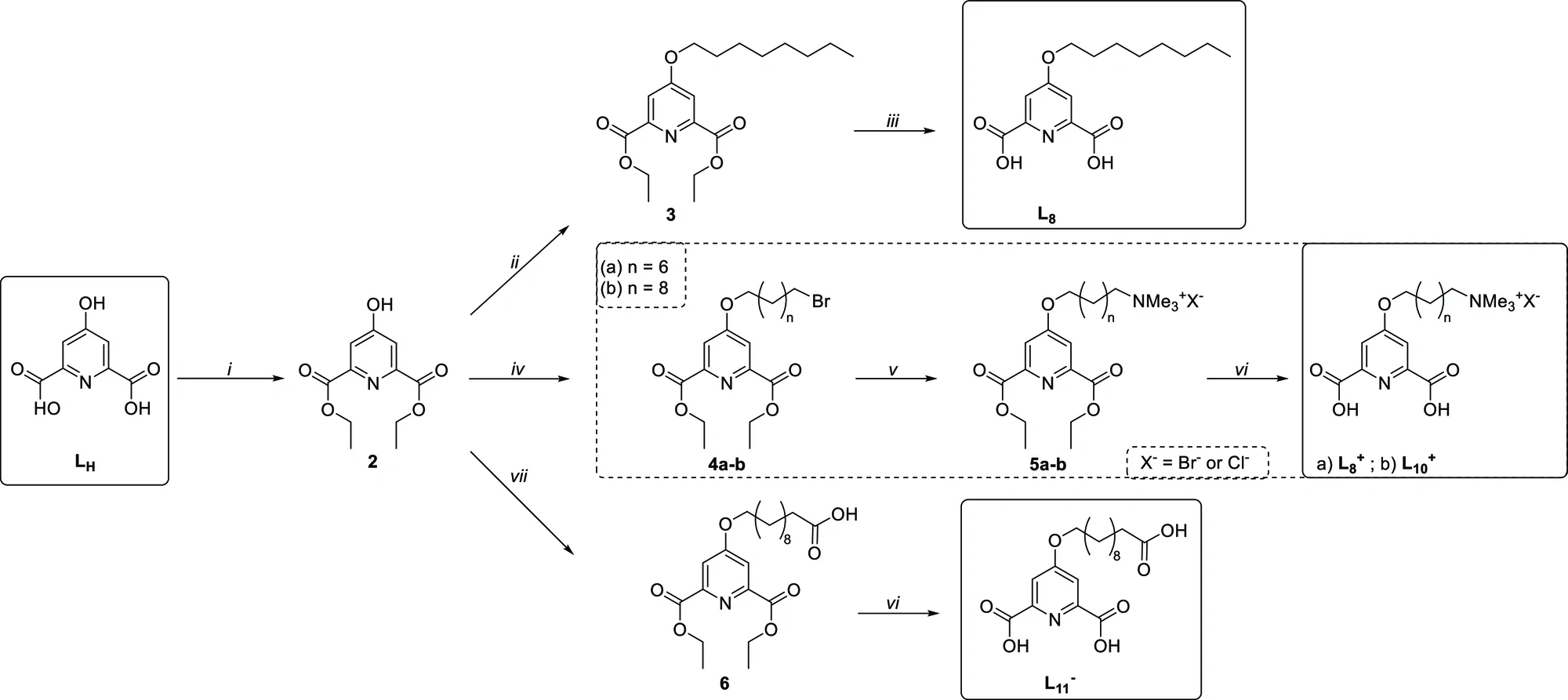
Synthetic Pathway for Different Ligands^a^

a (i) EtOH, H_2_SO_4_ 96%, reflux 4 h; (ii) 1-iodooctane, K_2_CO_3_, DMF, 90 °C, 20 h; (iii) KOH, EtOH/H_2_O r.t., 2 h, then HCl to reach pH = 4; (iv) 1,8-dibromooctane (or 1,10-dibromodecane), K_2_CO_3_, CH_3_CN, 80 °C, 3 h; (v) NMe_3_, EtOH, reflux, o.n.; (vi) KOH, THF/H_2_O r.t., 15 h, then HCl to reach pH = 4; (vii) methyl-11-bromoundecanoate, K_2_CO_3_, DMF, 90 °C, 20 h.

All the intermediate species and the ligands were characterized by NMR and FT-IR spectroscopies (in the Supporting Information). As an example, Figure 3 shows the ^1^H NMR spectrum of ligand **L**_**11**_^**–**^, featuring a singlet at 7.44 ppm attributable to the pyridinic ring protons. The triplet at 4.13 ppm corresponds to the −OCH_2_– group, while the triplet at 2.08 ppm is assigned to the −CH_2_– group α to the carbonyl moiety. Additional signals in the low-frequency region arise from the methylene protons of the alkyl chains. The spectra of the other ligands display a similar pattern of resonances with the expected variations. It should be noticed that for the cationic ligands **L**_**8**_^**+**^ and **L**_**10**_^**+**^, it has not been possible to control the exchange phenomena of the anionic counterion. Thus, they have been isolated as mixtures of **L**_**8/10**_^**+**^ X^–^ where X^–^ is Br^–^ or Cl^–^.

**Figure 3:**
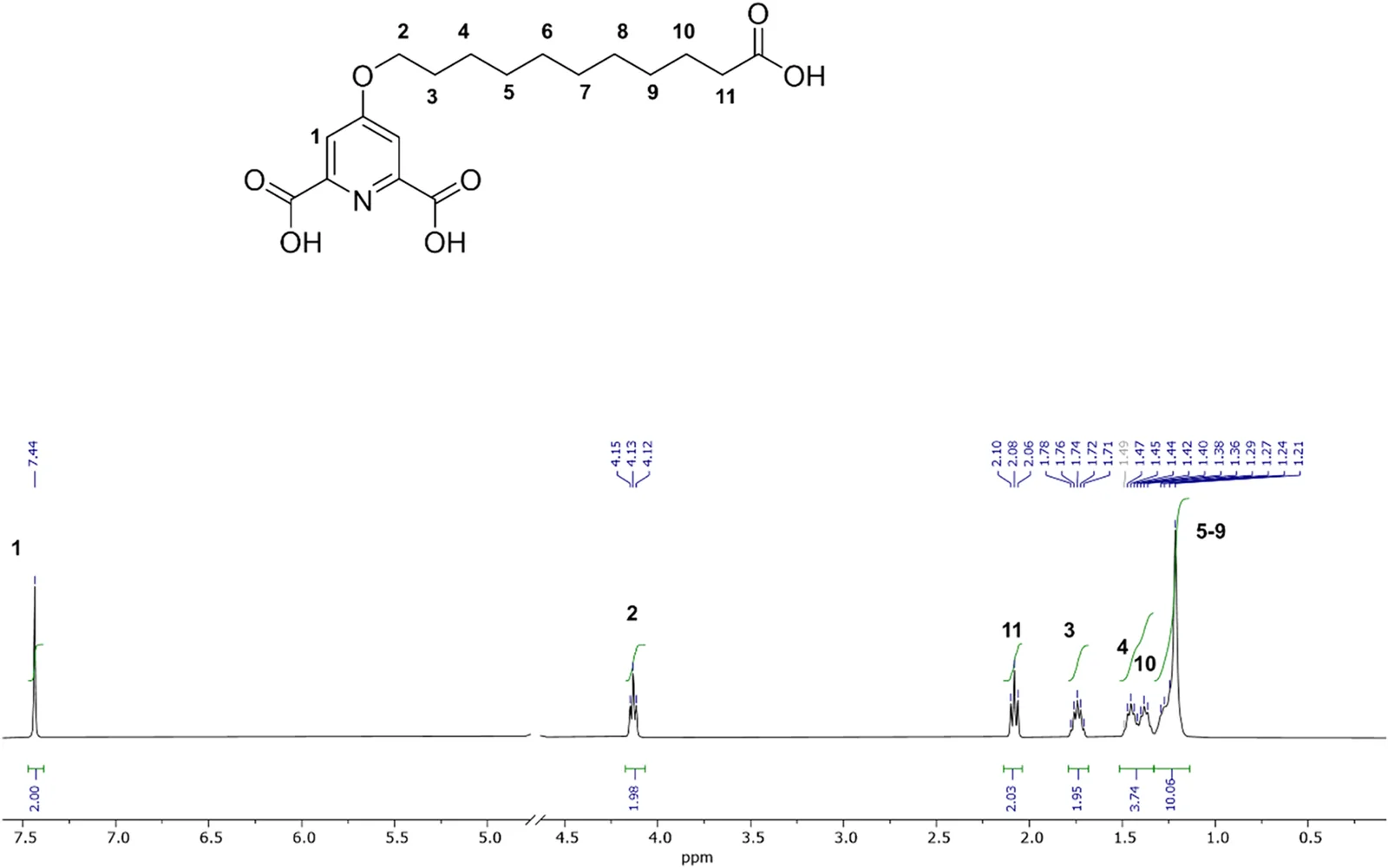
^1^H NMR spectrum of ligand **L**_**11**_^–^ (400 MHz, 25 °C, D_2_O).

The complexes were synthesized by the reaction of the appropriate chelidamic-type ligand with manganese­(II) acetate tetrahydrate (Scheme 2). The reactions were carried out in methanol under reflux for 3 h, after which the products either precipitated or were isolated by the addition of ether. The synthesized compounds were characterized by FT-IR, MS, and RXD of microcrystalline powders.

**Scheme 2:**
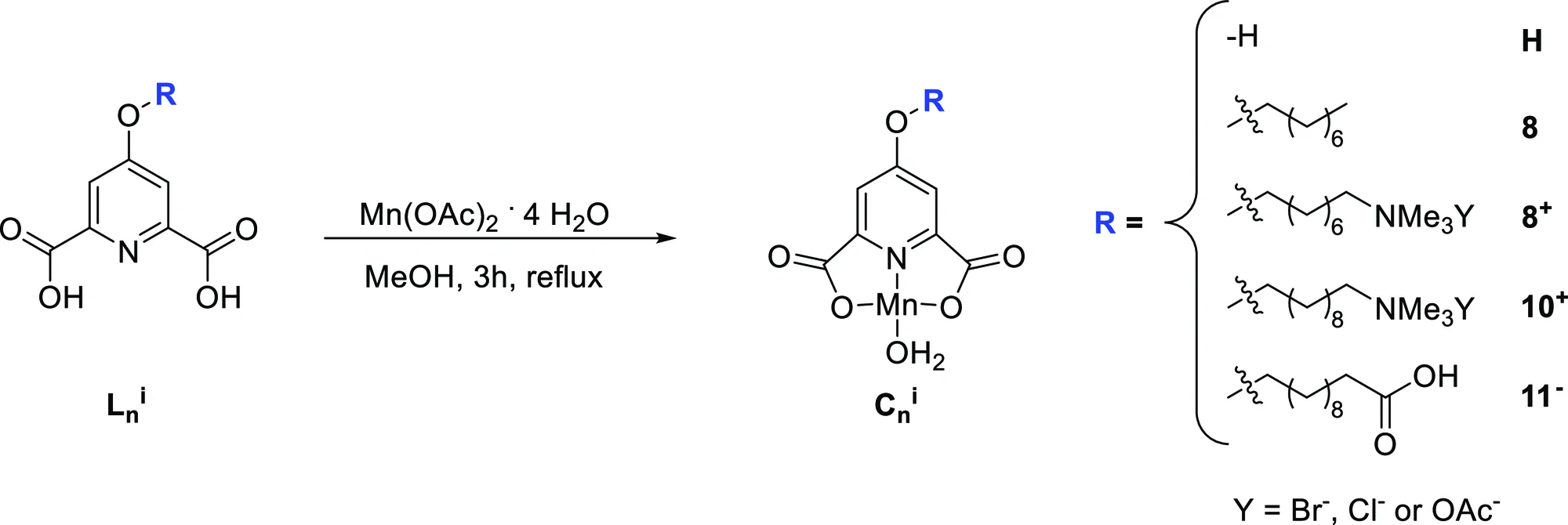
Synthetic Pathway of Different Manganese­(II) Complexes Derived from Chelidamic Acid Ligands

As an example, Figure 4 shows a comparison of the IR spectra of ligand **L**_**11**_^**–**^ vs complex **C**_**11**_^**–**^. The pronounced changes observed in the spectrum of the complex in the 1700–500 cm^–1^ region are attributed to coordination of the ligand to the metal, consistent with those reported in the literature for complexes containing dipicolinic ligands.^44–46^ The most notable difference appears in the region associated with carboxylate stretchings. Specifically, the ν­(C = O) bands of the free acid **L**_**11**_^**–**^ are converted into asymmetric and symmetric stretching vibrations of the carboxylate group upon coordination. These bands are shifted with respect to the ν­(C = O) vibrations of the free ligand and appear as two strong signals in the ranges 1550–1600 cm^–1^ and 1420–1300 cm^–1^. Another pronounced difference is observed in the 1000–500 cm^–1^ region. In particular, the band at 700 cm^–1^ in the ligand, assigned to the δ­(COO) deformation mode, shifts to higher frequency (around 730 cm^–1^) in the IR spectrum of the complex. The region between 2800 and 3000 cm^–1^ is attributed to the aromatic C–H stretching of the pyridine rings. For compound **C**_**11**_^**–**^, an additional band appears at approximately 3400 cm^–1^, arising from the O–H stretching vibrations of coordinated water molecules. This description is analogous for the other compounds.

**Figure 4:**
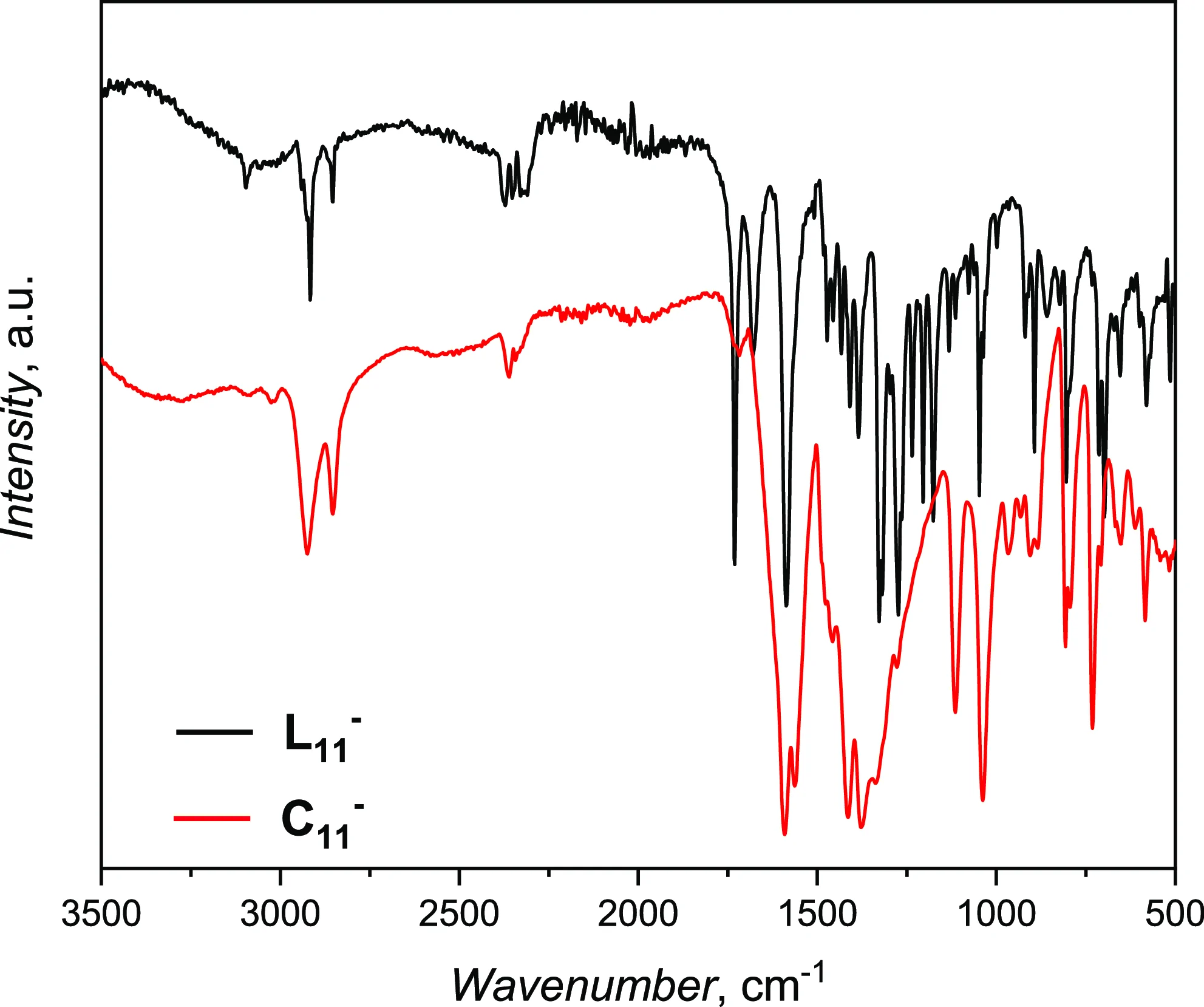
Comparison of FT-IR spectra between **L**_**11**_^–^ and **C**_**11**_^–^.

As illustrated in Figure 5, the WAXD patterns of the Mn­(II) complexes derived from chelidamic acid demonstrate that substitution at the 4-position of the ligand has a significant influence on the final crystalline organization. Traces a and b are both clearly crystalline and share intense reflections in the low-angle region, indicating related long-range packing motifs; this behavior is consistent with the known structural diversity of Mn–chelidamate solids, which commonly form hydrated one-dimensional coordination polymers sustained by carboxylate and/or aqua bridges, including staircase-like chains in triclinic P–1 as well as other hydrogen-bonded chain architectures.^43,47,48^ By contrast, trace c exhibits a new dominant low-angle reflection at 2θ = 8.6°, revealing the formation of a distinct phase with a larger characteristic repeat distance and therefore a more expanded lattice than in a and b, indicating that the complex **C**_**11**_^**–**^, which has a carboxyl terminal group, promotes substantial reorganization of the Mn–chelidamate assembly into a different crystalline phase. In this particular context, it is noteworthy to mention that chelidamate has recently been demonstrated to facilitate the development of a porous, three-dimensional Mn­(II) cage-based framework with large cavities.^49^

**Figure 5:**
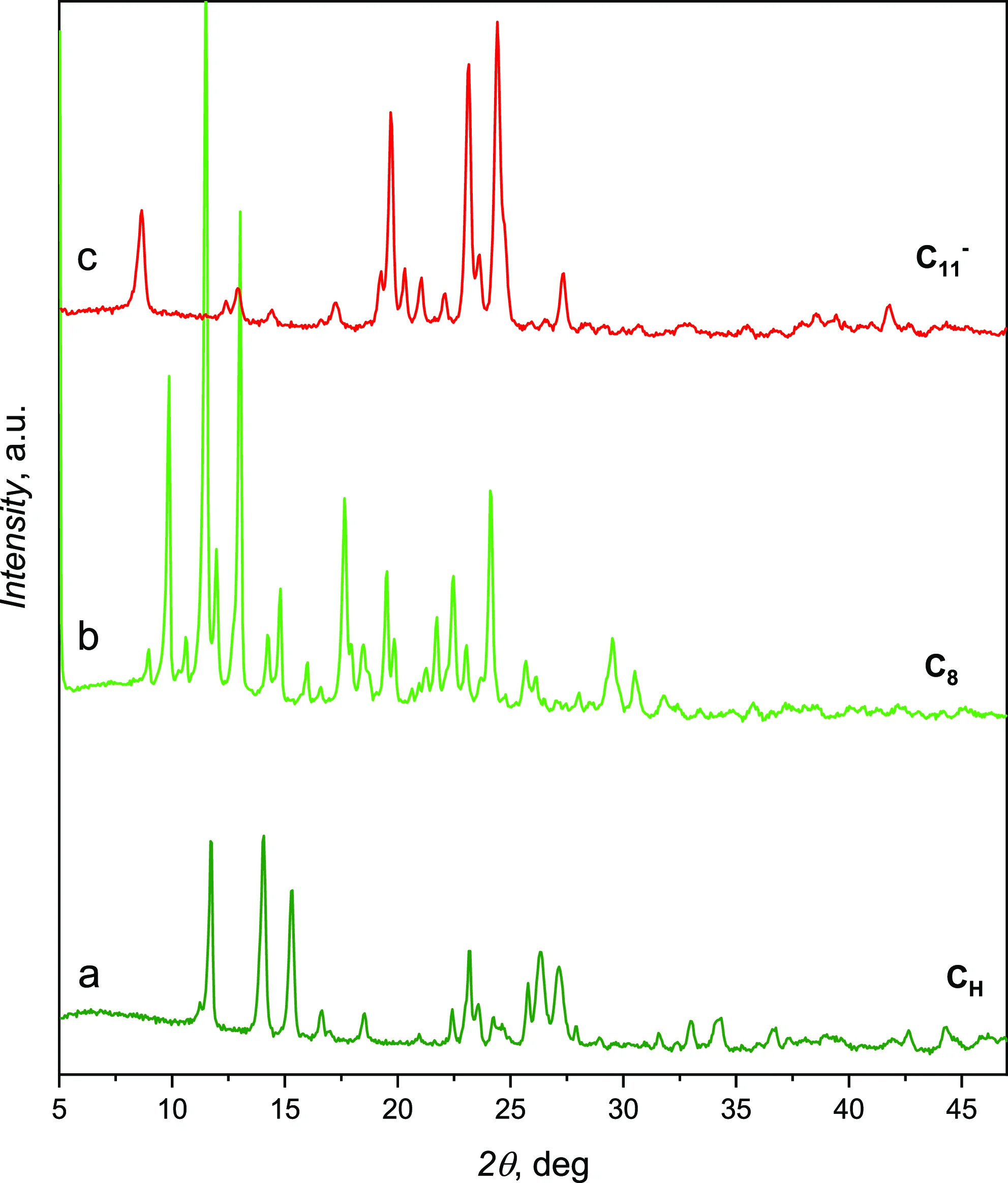
WAXD patterns recorded in the 5°–45° 2θ range using Cu Kα radiation (λ = 1.5418 Å) of Mn­(II) complexes derived from chelidamic acid and functionalized at the 4-position: (**a**) parent chelidamate, **C**_**H**_; (**b**) octyloxy derivative, **C**_**8**_; (**c**) and carboxyl derivative, **C**_**11**_^–^.

Finally, **C**_**8**_^**+**^ and **C**_**10**_^**+**^ WAXD profiles have shown a prominent diffusive background at low 2θ (data not reported), typical of amorphous compounds due to a poorly ordered organic fraction, suggesting that the quaternary-ammonium substituents strongly hinder the formation of a well-defined Mn–chelidamate coordination polymer. The poor crystallinity of the cationic complexes could also be due to the presence of different counter-anions, derived from the synthetic step for which it was not possible to limit exchange phenomena between Br^–^, Cl^–^, and OAc^–^.

### Catalytic Activity

#### Mn Catalysts and Buffer Concentration Screening

The catalytic activities of the five manganese complexes were compared in the bleaching of morin in buffered aqueous solution at pH 9.3, using hydrogen peroxide as an oxidant. The flavanol morin was chosen for these experiments since it is present in fruit and vegetables and is a target in the bleaching of laundry.^50^

The experimental setup is reported in Figure S30. In order to compare our data with similar catalytic systems,^39–41,51^ a morin (160 μM) aqueous buffered solution at pH 9.3 in the presence of a catalyst (2.65 μM) and sodium dodecyl sulfate (SDS) (150 μM) as surfactant was placed in a UV–vis cell. Then, hydrogen peroxide was injected and the reaction monitored over time following the maximum of absorption at 402 nm; see Figure 6. The concentrations used for the experiments were inspired by those conceivably acting during laundry processes, that is, less than 1 ppm of catalyst and 500 ppm of oxidant. The substrate–catalyst ratio was set at 60:1 at a temperature of 30 °C.

**Figure 6:**
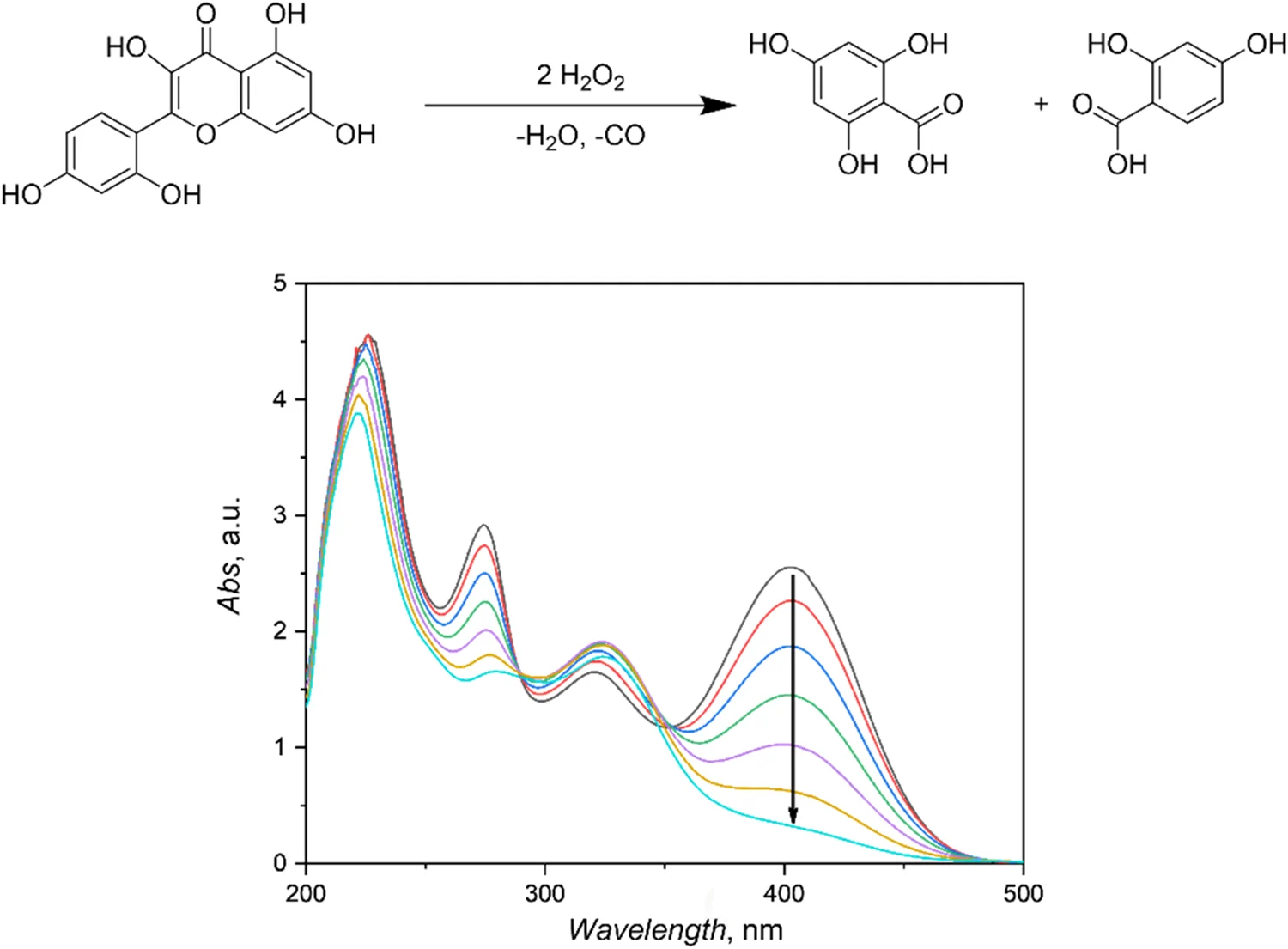
Representative monitoring of degradation of morin via UV–vis spectra in the following conditions: catalyst 2.65 μM, morin 160 μM, H_2_O_2_ 0.05% w/w, pH 9.3, 100 mM buffered solution, 30 °C.

The manganese complexes (**C**_**n**_^**i**^) exhibit a good catalytic activity. The variations observed among the catalytic performances indicate that *O*-substituents at the 4-position play a key role in determining the activity of the complexes. Indeed, catalysts bearing ligands substituted with any **R** group show higher activity compared to the free hydroxyl analogue, **C**_**H**_. Moreover, differences in the kinetic profiles are also observed among complexes bearing different **R** groups, indicating that not only the presence but also the nature of the substituents influences their catalytic behaviors.

Complexes **C**_**8**_, **C**_**10**_^**+**^, and **C**_**11**_^**–**^ exhibit the best performances, reaching approximately 95% conversion within 30 min (Figure 7), highlighting the prominent role of the *O*-alkyl chain over the end-of-chain charge. However, in the first 20 min of the reaction, complexes **C**_**8**_, **C**_**10**_^**+**^, and **C**_**11**_^**–**^ exhibit distinct kinetic profiles. Among these, complex **C**_**11**_^**–**^, which is present as the carboxylate species (**C**_**11**_^**–**^) at operating basic pH, with its anionic end-of-chain group, shows a slightly higher activity. Complex **C**_**10**_^**+**^, which has a longer alkyl chain compared to that of **C**_**8**_^**+**^ but the same cationic end-of-chain group, exhibits a more favorable kinetic profile. Also, two control experiments were conducted: one with H_2_O_2_ in the absence of a catalyst and another with manganese acetate. The latter is used as a precursor for the synthesis of complexes. In the first case, a poor decolorization of morin was observed in line with known phenomena of autoxidation of morin in basic media,^52^ while the second experiment proceeded with low conversion. Compared to the results reported in the literature, our system shows catalytically competitive performance using only 0.05% w/w hydrogen peroxide.^53,54^

**Figure 7:**
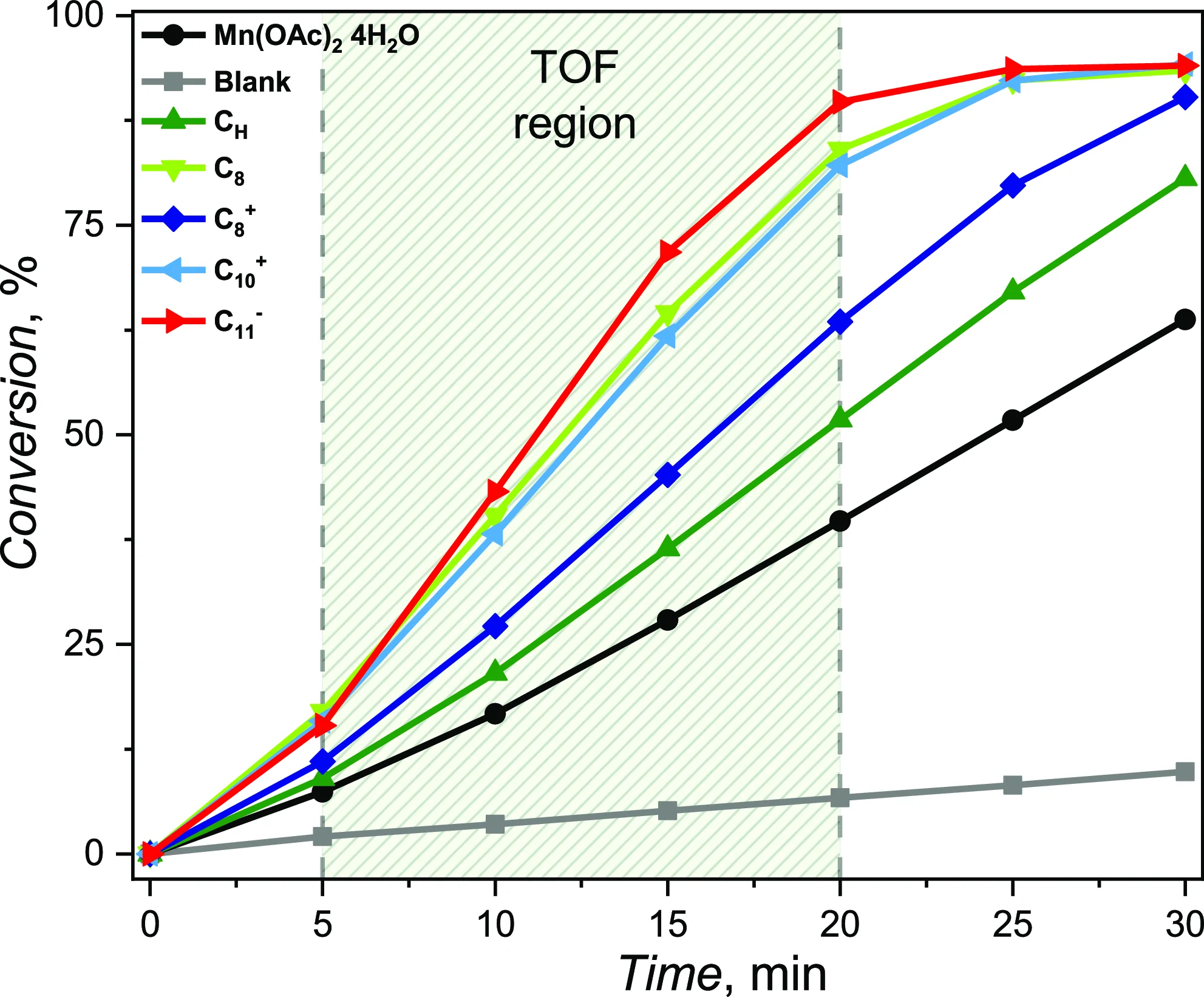
Catalytic activity comparison among all the complexes—catalyst 2.65 μM, morin 160 μM, H_2_O_2_ 0.05% w/w, pH 9.3, 100 mM buffered solution, 30 °C.

To enable a consistent comparison of catalytic performance, the analysis was focused between 5 and 20 min, where the reactions proceeded with quasi-linear kinetic profiles. This approach excludes the initial induction period (0–5 min), probably associated with catalyst activation, and the typical final plateau (20–30 min) related to high conversion rates. Within this selected time window (highlighted in Figure 7), linear fitting of the conversion versus time plots allowed the determination of an apparent average TOF (h^–1^) for each catalyst, reported in Table 1. The results confirm that the functionalization at the 4-position of the chelidamic acid scaffold plays a key role in tuning the catalytic activity, likely by modulating the interaction among the catalyst, substrate, and micellar environment, thereby affecting oxidant activation and substrate accessibility.

**Table 1:** TOF (h^–1^) Values and *R* ^2^

catalysts	TOF (h^–1^)	*R* ^2^
Mn(OAc)_2_·4H_2_O	81.4 ± 0.1	0.997
**C**_**H**_	103.0 ± 0.1	0.998
**C**_**8**_	162.0 ± 0.1	0.998
**C**_**8**_^**+**^	126.0 ± 0.1	0.999
**C**_**10**_^**+**^	160.2 ± 0.1	0.999
**C**_**11**_^**–**^	181.1 ± 0.3	0.990

The presence of an induction period in the first 5 min of reaction suggests that the catalytic system undergoes an activation phase before reaching its maximum efficiency. The presence of an induction period is often observed in the kinetic profiles for the activation of hydrogen peroxide; generally, this initial phase is due to the formation of the active species that allows bleaching of the dye.^22,55–57^ In this case study, the catalytic activity was evaluated in a bicarbonate-buffered solution; this latter has a noninnocent role in the activation of hydrogen peroxide, forming peroxymonocarbonate (HCO_4_^–^), a more active species than hydrogen peroxide. Aqueous CO_2_ in solution, in equilibrium with HCO_3_^–^, reacts with H_2_O_2_ and its conjugate base HOO^–^, in a perhydratation reaction that forms peroxymonocarbonate (HCO_4_^–^) (Figure 8A).^58^ In addition, Burgess et al.^59^ reported the higher reactivity of manganese salts in bicarbonate buffer using H_2_O_2_ as an oxidant. In particular, Burgess proposed the formation of peroxycarbonate-manganese species, which can attack directly the substrate, or an intermediate high-valent manganese-oxo species that transfers the oxygen atom to the substrate, as shown in Figure 8B.^59^ Both of these processes, which can occur during bleaching, can explain the presence of the induction period in the kinetics curve in Figure 7.

**Figure 8:**
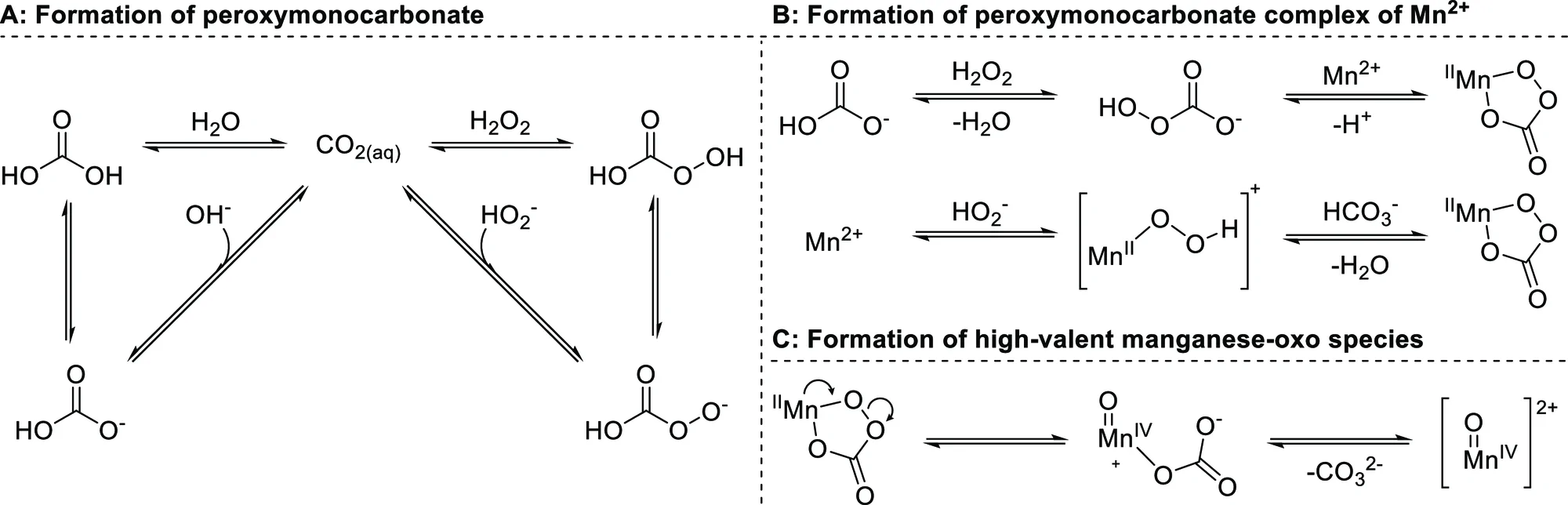
Formation of (A) peroxymonocarbonate; (B) peroxymonocarbonate complex of Mn^2+^; and (C) high-valent manganese-oxo species.

As further evidence of the above observations, the activity of the best catalyst, **C**_**11**_^**–**^ (hereinafter referred to as **C**_**11**_^**–**^ due to operative conditions), was evaluated in bicarbonate buffer at different concentrations (Figure 9). The best performances were achieved with the highest concentration tested (100 mM), while at 10 mM, a marked decrease in catalytic activity was observed. Eventually, at 1 mM, the reactivity is barely appreciable, achieving ≈2% of conversion. Overall, at low buffer concentrations, the induction period within the first reaction time is not observed (inset of Figure 9).

**Figure 9:**
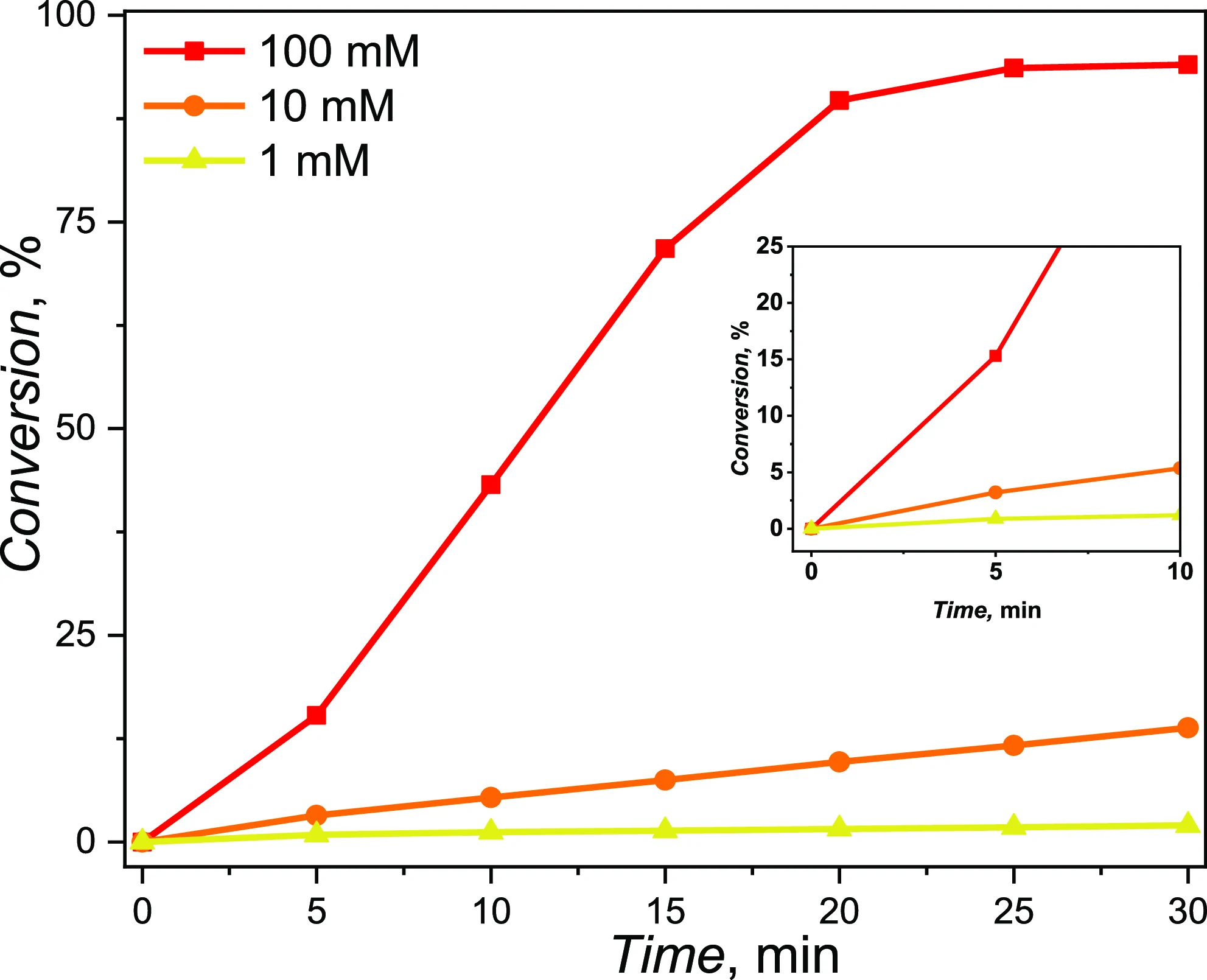
Catalytic bleaching activity of **C**_**11**_^–^ at different buffer concentrations—catalyst 2.65 μM, morin 160 μM, H_2_O_2_ 0.05% w/w, pH 9.3, 30 °C.

#### Effect of pH

The dependence on pH was also investigated for catalysts **C**_**8**_, **C**_**10**_^**+**^, and **C**_**11**_^**–**^. These complexes were selected because they showed the best catalytic performance at 30 min of reaction (see Figure 7) and because they bear three different chains with different charges. The study was carried out starting from a buffered solution of 100 mM bicarbonate at pH 8.3, and the catalytic activity was evaluated at pH values varied in increments of 0.5 units (Figure 10A). The conversion shows a bell-shaped trend as a function of pH, reaching a maximum at pH 9.3. Given the p*K*_a_ values of morin (3.5 and 8.1), protonation of the substrate below pH 9.3 could be one of the factors contributing to the decrease in the catalytic activity.^39,60^ The p*K*_a_ of hydrogen peroxide is 11.64, which means that at high pH the equilibrium H_2_O_2_/HO_2_^–^ lies toward HO_2_^–^, which presents greater nucleophilicity than hydrogen peroxide. This would suggest an increase in activity as the pH increases, due to the nucleophilicity of OOH^–^; however, high pH values can compromise the stability of the catalysts by the formation of manganese oxides.^14^ The combination of these factors influences the pH dependence of the bleaching process. In general, it is difficult to discuss the pH dependence by considering each of the aspects mentioned above individually. Nevertheless, the trend in catalytic activity as a function of pH observed for complexes **C**_**8**_, **C**_**10**_^**+**^, and **C**_**11**_^**–**^ is generally consistent with that reported for other manganese­(II) complexes and for Mn–Me_3_tacn, showing a strong activation of hydrogen peroxide at moderately alkaline pH values and a decrease in activity at increasingly acidic or more alkaline conditions.

**Figure 10:**
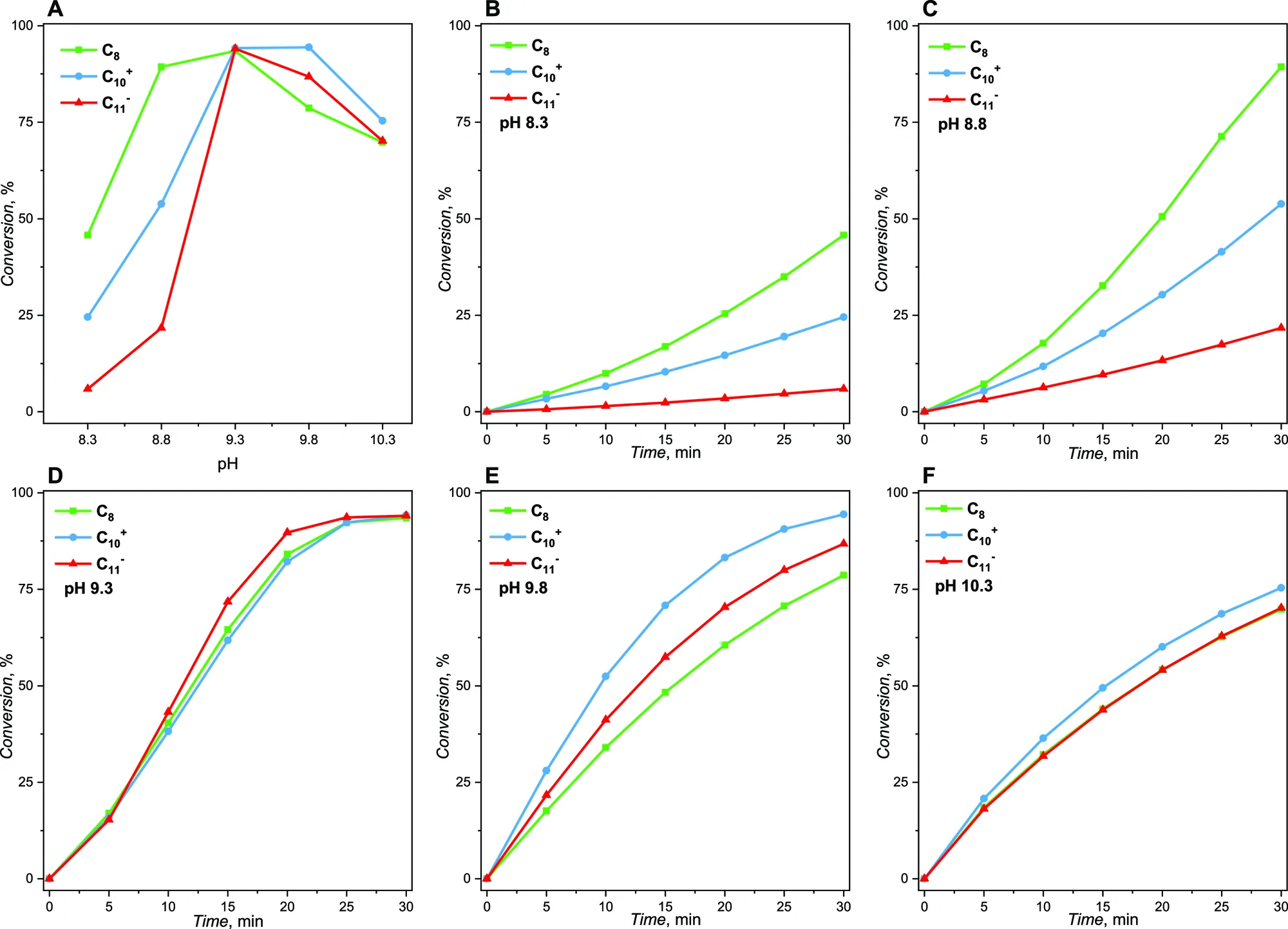
(A) pH dependence of **C**_**8**_, **C**_**10**_^**+**^, and **C**_**11**_^–^ at 30 min and (B–F) kinetic profiles at different pH values—catalyst 2.65 μM, morin 160 μM, H_2_O_2_ 0.05% w/w, 100 mM buffered solution, 30 °C.

Interestingly, the three complexes present different behaviors within the investigated pH range. At low pH values (around 8.3–8.8), the conversion remains modest for all three series, indicating that the reaction is not yet under optimal conditions. As the pH approaches 9.3, all curves rise sharply and converge to nearly full conversion, revealing a clear optimal pH where the process is most efficient. Beyond this point, with an increase in basicity, the conversion decreases again. Despite following the same overall trend, the three complexes show some differences in their sensitivity to pH. The neutral-chain-containing complex **C**_**8**_ displays the highest activity at lower pH values, with a maximum in activity between pH 8.8 and pH 9.3. In general, its catalytic behavior also appears to be less affected by pH variations. The cationic-chain-containing complex **C**_**10**_^**+**^ shows a smoother increase as the pH rises, with a maximum in activity at pH 9.8. In contrast, the anionic-chain-containing complex **C**_**11**_^**–**^, which appears to be the most sensitive to pH variations, is the least active at lower pH but exhibits a marked increase in activity around pH 9.3, possibly related to the deprotonation of its terminal carboxylic group. At this pH, **C**_**11**_^**–**^ showed the best kinetic profile (Figure 10B–F, red lines).

The extension of the kinetic study about the dependence on temperature, loading of catalyst, and loading of hydrogen peroxide was conducted using catalyst **C**_**11**_^***–***^.

#### Temperature Dependence

The temperature dependence of the activity of **C**_**11**_^**–**^ was evaluated, as shown in Figure 11. As expected, at temperatures below 30 °C, the catalytic activity decreased, although it remained significant. Notably, at 40 °C a reduction in the induction period was observed, a phenomenon already known in the literature.^61–65^

**Figure 11:**
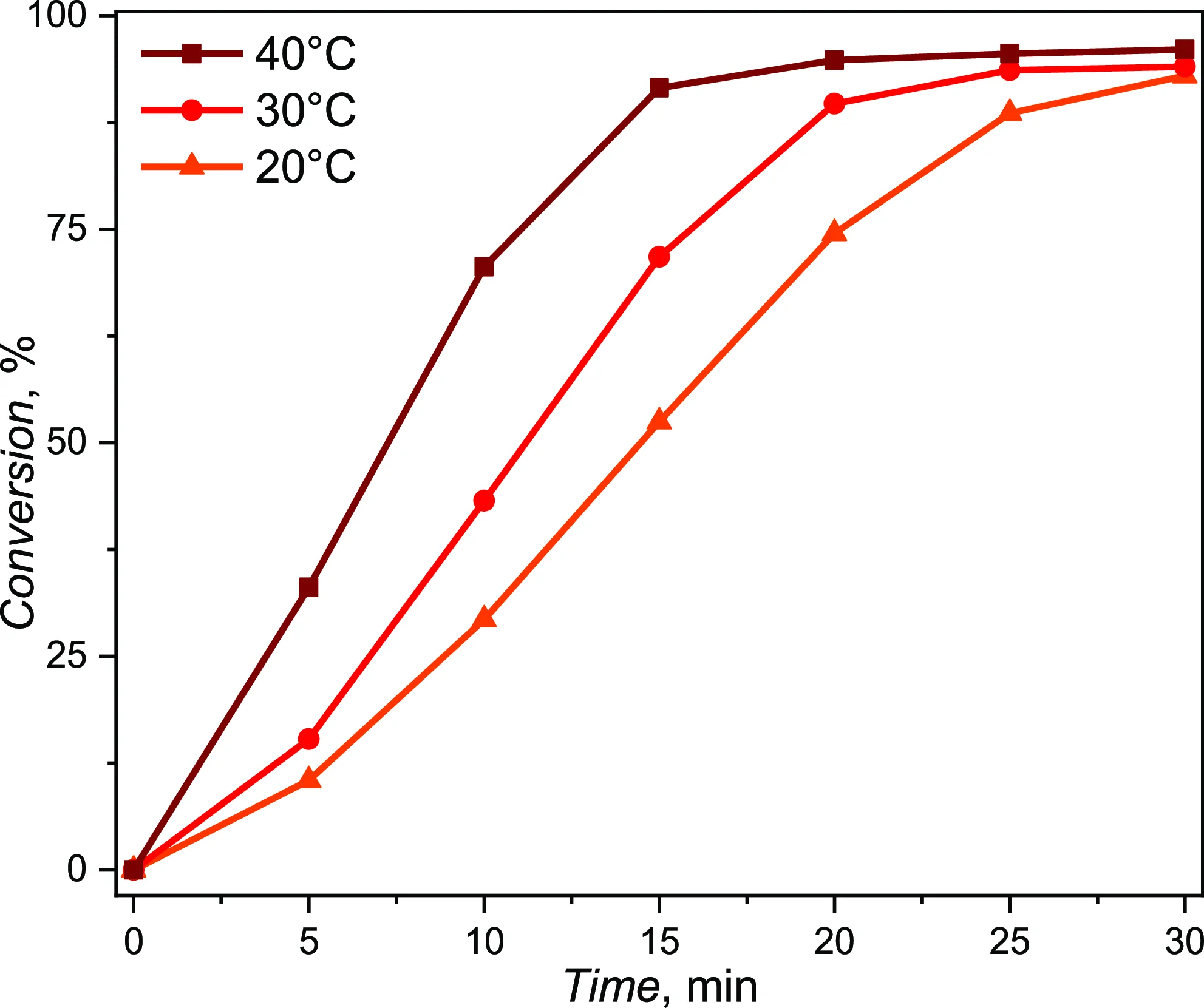
Dependence on temperature for **C**_**11**_^–^—catalyst 2.65 μM, morin 160 μM, H_2_O_2_ 0.05% w/w, pH 9.3, 100 mM buffered solution.

#### Dependence on Catalyst and Hydrogen Peroxide Concentration

The dependence on the catalyst (Figure 12) and hydrogen peroxide (Figure 13) loadings was also investigated using complex **C**_**11**_^**–**^. At lower catalyst concentration, a decrease in catalytic activity is observed. In particular, a drastic reduction was observed at a concentration of 0.80 μM, while at 1.60 μM, the activity remained significant. There is also a strong dependence of the catalytic activity on the loading of hydrogen peroxide, which shows a significant decrease simply by reducing the amount from 0.05% to 0.04% w/w.

**Figure 12:**
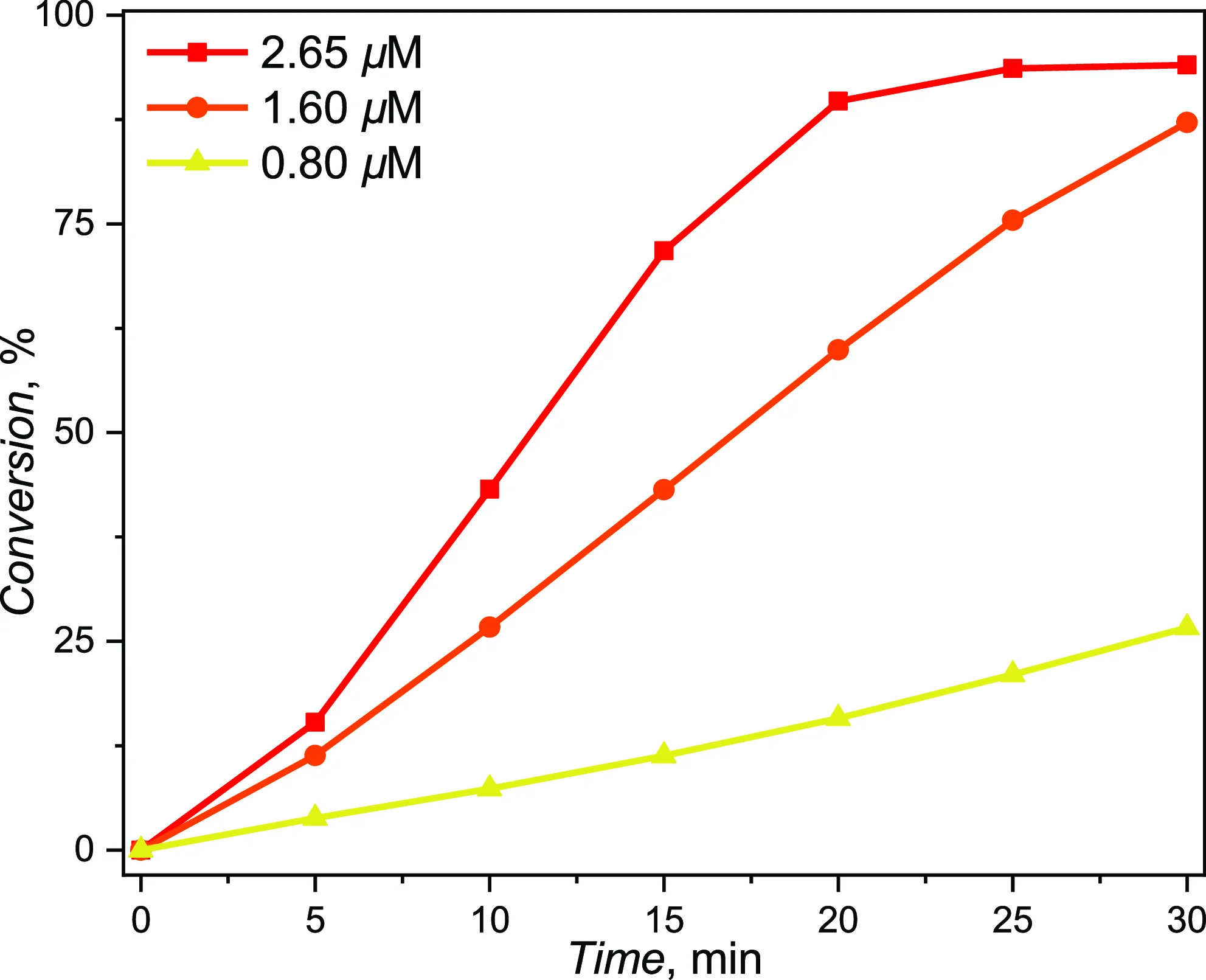
Dependence on catalyst concentration, **C**_**11**_^–^—morin 160 μM, H_2_O_2_ 0.05% w/w, pH 9.3, 100 mM buffered solution, 30 °C.

**Figure 13:**
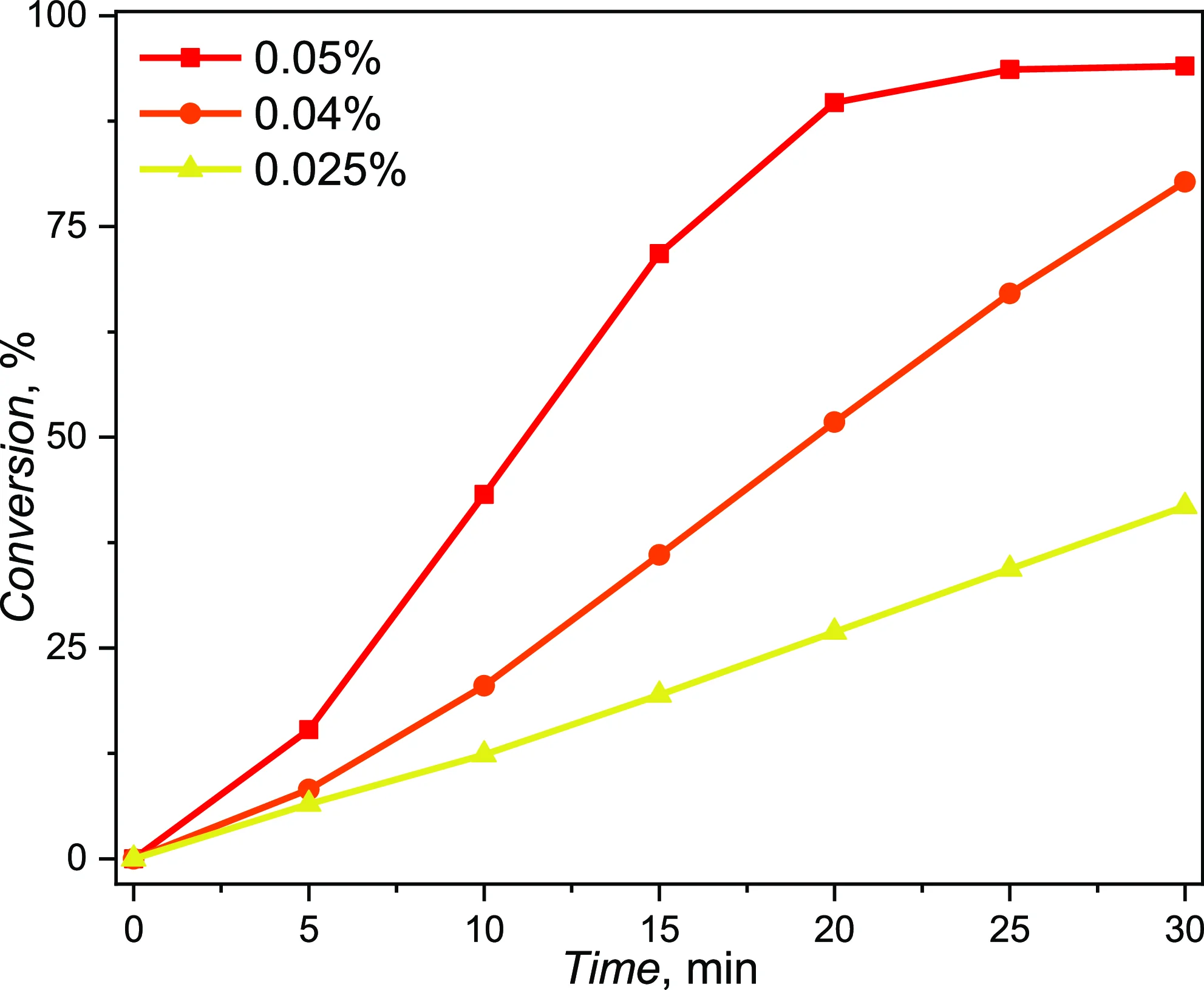
Dependence on H_2_O_2_ concentration for complex **C**_**11**_^–^—catalyst 2.65 μM, morin 160 μM, pH 9.3, 100 mM buffered solution, 30 °C.

The inflection point associated with the induction period becomes less pronounced at lower concentrations of both the catalyst (Figure 12) and oxidant (Figure 13). Probably, unlike the kinetic considerations discussed for the previous case (dependence on temperature), here the reduced visibility of the induction period inflection is due to the suppression of the oxidation kinetics, whose slope progressively approaches that of the induction phase. Also, at lower concentrations of the catalyst, the effect of the autoxidation, not being negligible anymore, can contribute to plot linearization.

#### Extending the Substrate Scope: Oxidation of the Azo Dye Sunset Yellow FCF

Azo dyes constitute the largest class of synthetic colorants used in industrial applications, particularly in textile processing, and are frequently detected in wastewater streams because of their high solubility and chemical stability.^66^ This class of compounds is difficult to remove under conventional treatments in wastewater treatment plants.^67,68^ Among them, Sunset Yellow FCF (SY) is widely employed as a colorant in food, drugs, and cosmetics. It is a water-soluble azo dye that is often used as a model compound in degradation studies due to its well-defined chromophore and spectroscopic properties. Despite its relatively simple structure compared to more complex azo dyes, its degradation still requires efficient oxidative systems capable of cleaving the azo bond or disrupting the conjugated system responsible for its color.^69^

To further evaluate the versatility of the investigated Mn-bleaching system, its performance was investigated in the oxidation of SY under the same mild aqueous conditions employed for morin bleaching. Complex **C**_**11**_^**–**^ was selected to evaluate the ability of manganese­(II) complexes derived from chelidamic acid to oxidize structurally different and more recalcitrant substrates, such as azo compounds. The experimental setup is the same as that reported in Figure 6A, and previously optimized conditions for morin bleaching were used (30 °C, buffer 100 mM). A Sunset Yellow FCF (160 μM) aqueous buffered solution at pH 9.3 in the presence of the catalyst **C**_**11**_^**–**^ (2.65 and 5.33 μM) and SDS as the surfactant were placed in a UV–vis cell. Then, hydrogen peroxide (concentration 0.05% w/w) was injected and the reaction monitored over time following the maximum of absorption at 481 nm; see Figure 14.

**Figure 14:**
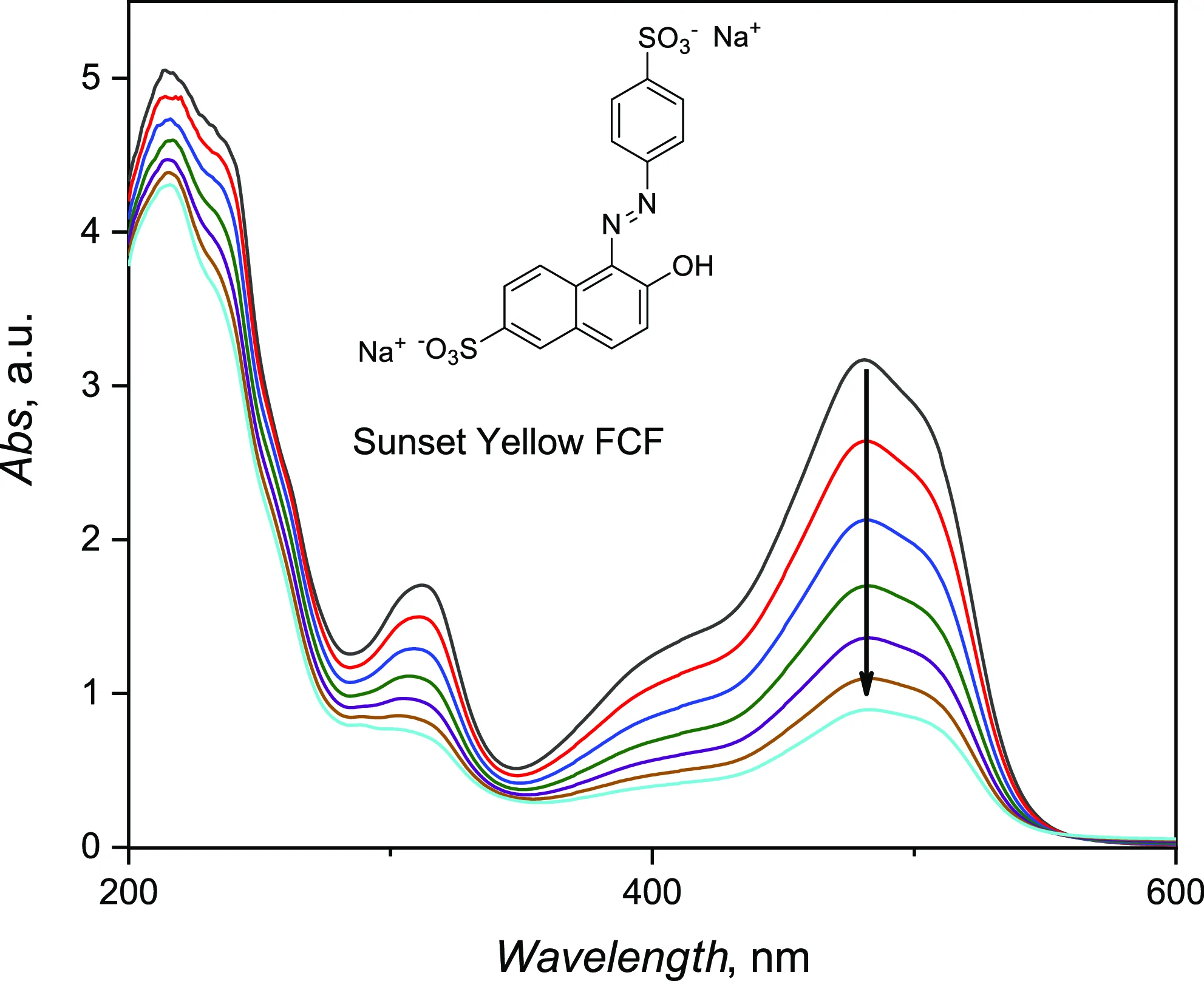
Representative monitoring of Sunset Yellow FCF (SY) oxidation via UV–vis spectra in the following conditions: catalyst 5.33 μM, Sunset Yellow FCF 160 μM, H_2_O_2_ 0.05% w/w, pH 9.3, 100 mM buffered solution, 30 °C.

The SY oxidation was monitored over time (Figure 15) at two substrate–catalyst ratios, 60:1 and 30:1, corresponding to the two used catalyst concentrations (2.65 and 5.33 μM). With the lower substrate–catalyst ratio (60:1), a significant conversion was already achieved at 35 min (60%) and was further improved with the higher ratio (30:1), achieving 77% conversion within the same time. Unlike the oxidation of morin, in the kinetic profile for the oxidation of SY, the induction period is not present. This disappearance can be justified as a different interaction between the azo-compound and the manganese catalyst; in fact, in previous studies, the coordination of azo dyes to manganese­(II) salts is reported during degradation processes.^70,71^ This substrate–metal interaction could alter the catalyst activation pathway and consequently affect the reaction kinetics, leading to the absence of the induction period. These results, achieved in nonspecifically optimized conditions, are particularly remarkable, especially considering the intrinsic difficulty associated with the degradation of azo compounds, and comparing them with the literature data obtained under significantly harsher conditions. In particular, manganese­(II)-based systems reported for azo dye degradation using hydrogen peroxide often achieve complete decolorization only under forcing conditions. These typically involve high substrate-to-catalyst ratios (often exceeding 30:1 and in some cases approaching near-stoichiometric values, e.g., 3:1), together with a large excess of hydrogen peroxide (up to 500–10,000 equiv relative to the catalyst), and more alkaline conditions (pH 10–11).^54,57,70^ In contrast, the present system operates under milder and more sustainable conditions, employing a moderate substrate-to-catalyst ratio and a low hydrogen peroxide loading.

**Figure 15:**
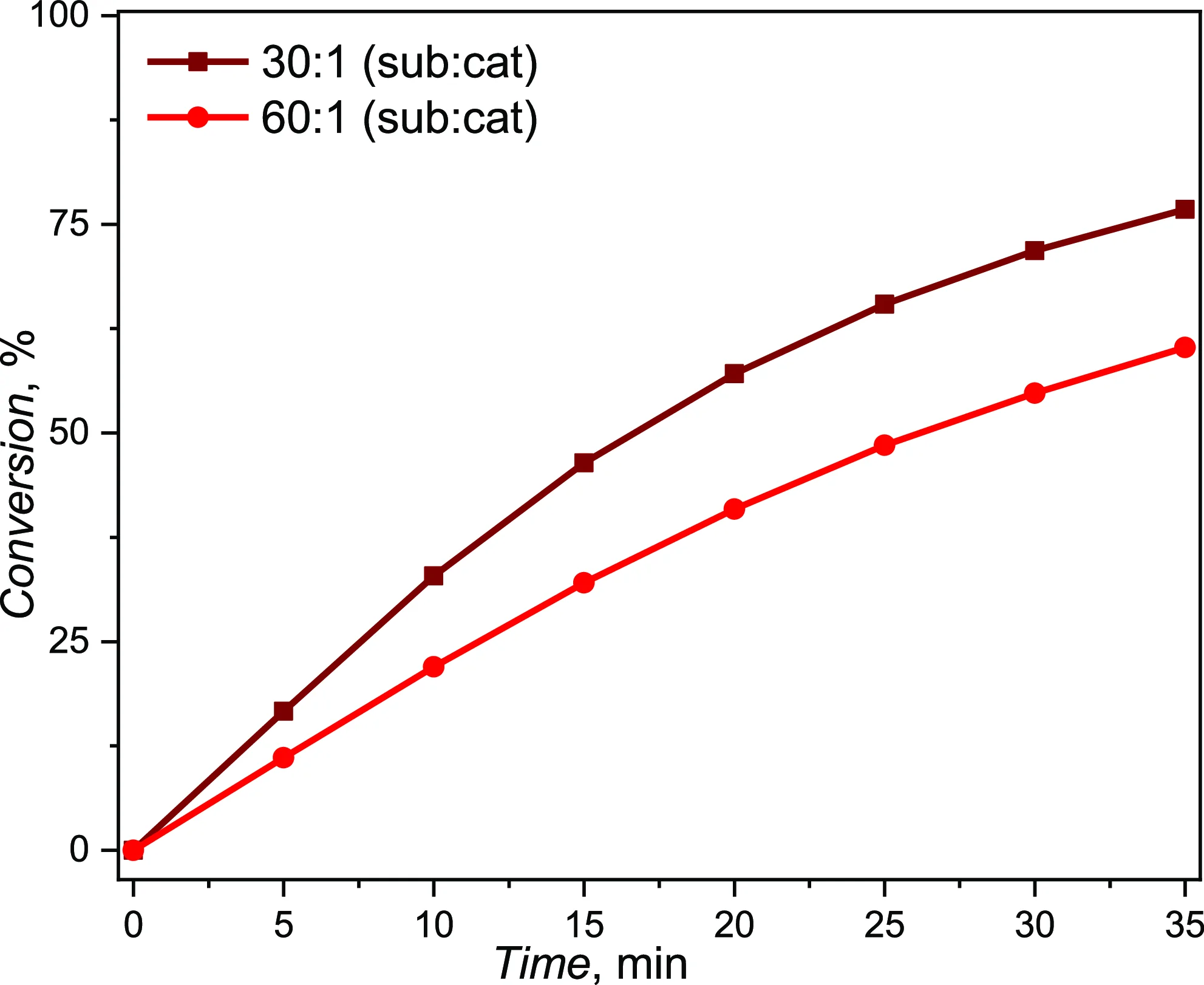
Catalytic activity of **C**_**11**_^–^ in the oxidation of Sunset Yellow FCF—catalyst 2.65–5.33 μM, Sunset Yellow FCF 160 μM, H_2_O_2_ 0.05% w/w, 100 mM buffered solution, 30 °C.

## Experimental Section

### General

All solvents and reagents were purchased from Merck Life Sciences and used without further purification. NMR spectra were recorded in CDCl_3_, D_2_O, and DMSO using a Bruker 400 spectrometer. The following abbreviations are used for describing NMR multiplicities: s, singlet; dd, double doublet; ddd, double double doublet; dq, double quartet; t, triplet; dt, double triplet; td, triple doublet; m, multiplet. UV–vis spectra were recorded with a JASCO V770 spectrophotometer. IR spectra of the complexes were recorded using a FT-IR Spectrophotometer Anton Paar Lyza 7000. RXD spectra of the powder of the complexes were recorded using a Malvern Panalytical Empyrean X-ray Diffractometer with WAXD patterns recorded in the 5°–45° 2θ range using Cu Kα radiation (λ = 1.5418 Å). Each sample was introduced into a liquid chromatography-mass spectrometry (LC-MS) apparatus via an Agilent 6230 TOF mass spectrometer coupled to a Agilent HPLC system (1260 Series). The HPLC separation was carried out on a reverse-phase C18 column (Poroshell 120 EC-C18 3 × 50 mm 2.7 μm from Agilent Life Sciences) by using water and acetonitrile as mobile phases A and B, respectively, both acidified with 0.1% formic acid. A linear gradient was employed by increasing the mobile phase B from 5 to 95% over 20 min. The flow rate was 0.3 mL/min and the injection volume was 20 μL. The mass spectrometer was equipped with an electrospray ionization (ESI) source in positive ion mode. Instrument parameters were set as follows: capillary voltage of 3500 V, gas temperature at 325 °C, dry gas (N_2_) flow at 5 L/min, nebulizer pressure at 35 psi, and fragmentor voltage at 150 V. Spectra were recorded with a rate of 1 spectrum/s over a mass range of 50–1000 *m*/*z*.

### Synthesis of the Ligands

#### Synthesis of Diethyl 4-Hydroxypyridine-2,6-dicarboxylate (**2**)

Chelidamic acid (**L**_**H**_; 24.6 mmol, 4.5 g) was suspended in absolute ethanol (90 mL), and 0.9 mL of sulfuric acid (96%) was carefully added at room temperature with vigorous stirring. The yellow mixture was refluxed for 4 h and the solvents evaporated. The viscous residue was treated with a saturated solution of sodium hydrogen carbonate until pH 8 and the aqueous solution was extracted with dichloromethane (4 × 200 mL). The organic phase was dried with sodium sulfate and evaporated to give diethyl 4-hydroxypyridine-2,6-dicarboxylate (**2**) as a pale solid, which solidified upon standing at room temperature (3.0 g, 50%).^72^ The low yield is due to the formation of monoester as a byproduct, which is difficult to further convert. ^1^H NMR (400 MHz, CDCl_3_) δ 7.36 (s, 2H, py.), 4.44 (q, *J* = 7.1 Hz, 4H, py-COOC*H*_2_CH_3_), 1.40 (t, *J* = 7.2 Hz, 6H, py-COOCH_2_C*H*_3_).

#### Synthesis of Diethyl 4-hydroxypyridine-2,6-dicarboxylic Acid (**L**_***8***_)

Diethyl 4-hydroxypyridine-2,6-dicarboxylate (**2**, 5.5 g, 14.6 mmol), 1-iodooctane (3.9 mL, 22.0 mmol), and potassium carbonate (4.0 g, 29.2 mmol) were dissolved in DMF (80 mL). The reaction mixture was stirred at 90 °C for 20 h and then cooled at room temperature. The solids were filtered off, and DMF was removed under vacuum.^73^ The crude product **3** was dissolved in ethanol (200 mL) in an ice bath, and a solution of potassium hydroxide (2.3 g, 40.3 mmol) in ethanol:water mixture (90:10 v/v) was added to the cooled solution. The resulting suspension was stirred at room temperature for 2 h. Then, ethanol was removed under reduced pressure and an aqueous HCl solution (0.1 M) was added to the obtained aqueous phase dropwise until a pH value of 1 was reached. The white precipitate was collected by vacuum filtration and dried at 70 °C to afford product **L**_**8**_ (3.2 g, 70%).^74^ ^1^H NMR (400 MHz, D_2_O) δ 7.83 (d, *J* = 0.7 Hz, 2H, py.), 4.46 (t, *J* = 6.5 Hz, 2H, py-OC*H*_2_−), 1.91 (p, *J* = 6.7 Hz, 2H, –OCH_2_C*H*_2_−), 1.50 (p, *J* = 7.1 Hz, 2H, −C*H*_2_−), 1.43–1.24 (m, 8H, −(C*H*_2_)_4_−), 0.91–0.81 (m, 3H, −C*H*_3_); ^13^C NMR (400 MHz, D_2_O) δ 172.84 (py-*C*OOH), 166.79 (*C*_4_–OH, pyridine ring), 154.88 (*C*_2_–*C*_6_, pyridine ring), 111.42 (*C*_3_–*C*_5_, pyridine ring), 68.96 (py-O*C*H_2_−), 31.03 (−*C*H_2_−), 28.33 (−*C*H_2_−), 28.32 (−*C*H_2_−), 27.97 (−*C*H_2_−), 25.03 (−*C*H_2_−), 21.98 (−*C*H_2_−), 13.38­(−*C*H_3_).

#### Synthesis of Diethyl 4-(Bromoalkoxy)­pyridine-2,6-dicarboxylate (**4a**–**y**): Example—Synthesis of Diethyl 4-(8-Bromooctyloxy)­pyridine-2,6-dicarboxylate

Diethyl 4-hydroxypyridine-2,6-dicarboxylate (**2**, 10.0 mmol, 2.5 g) was added to a suspension of anhydrous potassium carbonate (50 mmol, 15.0 g) in acetonitrile (50 mL), and then 1,8-dibromooctane (50 mmol, 11.2 mL) was slowly added. The resulting mixture was stirred at 80 °C for 3 h, the reaction was quenched with water (100 mL) and extracted with ethyl acetate (3 × 100 mL), the organic phase was washed with sodium carbonate 1 M (3 × 100 mL) and with a saturated solution of sodium chloride (3 × 100 mL), dried with anhydrous sodium sulfate, and concentrated. The oily residue was then purified thought column chromatography with hexane/ethyl acetate 1:2 as eluent to afford pure **4a** (Rf: 0.40; 2.9 g, 60%).^75^ The low yield obtained is due to the formation of a byproduct, which is the product of disubstitution of the dibromoalkyl by the phenolic oxygen of the chelidamic ester; the same is for compound **4b**. ^1^H NMR (400 MHz, CDCl_3_) δ 7.78 (s, 2H, py.), 4.48 (q, *J* = 7.1 Hz, 4H, py-COOC*H*_2_CH_3_), 4.14 (t, *J* = 6.4 Hz, 2H, py-OC*H*_2_–), 3.43 (t, *J* = 6.8 Hz, 2H, −C*H*_2_Br), 1.93–1.78 (m, 5H, −C*H*_2_–), 1.55–1.33 (m, 13H, −C*H*_2_– and py-COOCH_2_C*H*_3_).

**4b** ^1^H NMR (400 MHz, CDCl_3_) δ 7.76 (s, 2H, py), 4.51 (q, *J* = 7.1 Hz, 4H, py-COOC*H*_2_CH_3_), 4.12 (t, *J* = 6.5 Hz, 2H, py-OC*H*_2_–), 3.40 (td, *J* = 6.9, 1.0 Hz, 2H, −C*H*_2_Br), 1.92–1.76 (m, 4H, −C*H*_2_–), 1.51–1.27 (m, 18H, −C*H*_2_- and py-COOCH_2_C*H*_3_).

#### Synthesis of 4-(Trimethylammonioalkoxy)­pyridine-2,6-dicarboxylate Bromide (**5a**–**b**): Example—Synthesis of Diethyl 4-(8-(Trimethylammonio)­octoxy)­pyridine-2,6-dicarboxylate Bromide

Compound **4a** (4.8 g, 11.0 mmol) and trimethylamine (4.2 M in ethanol, 4.0 mL, 16.6 mmol) were added to ethanol (20.0 mL), and the resulting solution was refluxed overnight. Then, the solvent was removed by evaporation under vacuum, and the residue yellow oil was washed with ethyl ether to give pure **5a** (5.7 g, 99%).^76^ ^1^H NMR (400 MHz, CDCl_3_) δ 7.76 (d, *J* = 1.0 Hz, 2H, py.), 4.47 (q, *J* = 7.2 Hz, 4H, py-COOC*H*_2_CH_3_), 4.13 (t, *J* = 6.3 Hz, 2H, py-OC*H*_2_–), 3.65–3.58 (m, 2H,–C*H*_2_NMe_3_^+^Br^–^), 3.47 (s, 9H, –N­(C*H*_3_)_3_^+^Br^–^), 2.00 (br., 4H, –(C*H*_2_)_2_–), 1.90–1.74 (m, 4H, –(C*H*_2_)_2_–), 1.54–1.35 (m, 10H, –(C*H*_2_)_2_– and py-COOCH_2_C*H*_3_).

**5b** ^1^H NMR (400 MHz, CDCl_3_) δ 7.76 (s, 2H, py.), 4.46 (q, *J* = 7.1 Hz, 4H, py-COOC*H*_2_CH_3_), 4.13 (t, *J* = 6.4 Hz, 2H, py-OC*H*_2_–), 3.57 (d, *J* = 17.2 Hz, 2H, −C*H*_2_NMe_3_^+^Br^–^), 3.46 (s, 9H, –N­(C*H*_3_)_3_^+^Br^–^), 1.73 (m, 8H, –(C*H*_2_)_4_–), 1.45 (t, *J* = 7.1 Hz, 6H, py-COOCH_2_C*H*_3_), 1.40–1.28 (m, 8H, –(C*H*_2_)_4_–).

#### Synthesis of 4-(Trimethylammonioalkoxy)­pyridine-2,6-dicarboxylic Acid Bromide (**L**_***n***_^***+***^): Example—Synthesis of 4-[8-(Trimethylammonio)­octyloxy]­pyridine-2,6-dicarboxylic Acid Bromide, **L**_**8**_^**+**^

The synthesis of this compound was adapted from the procedure described by Balkenende et al.^73^ A solution of KOH (0.34 g, 6.13 mmol) in water (20 mL) was added to a solution of compound **5a** (2.10 mmol, 1.03 g) in 60 mL of THF, and the resulting mixture was stirred at room temperature for 16 h. THF was then removed under vacuum, and the aqueous phase was washed three times with dichloromethane. An aqueous HCl solution (0.1 M) was added dropwise to the aqueous phase until a pH value of 4 was reached. Water was distilled off and the white solid residue was extracted with MeOH. After filtration, removal of the solvent under vacuum afforded pure **L**_**8**_^**+**^ (0.71 g, 91%). ^1^H NMR (400 MHz, CD_3_OD) δ 7.81 (s, 2H, py.), 4.26 (t, *J* = 6.3 Hz, 2H, py-OC*H*_2_–), 3.41–3.32 (m, 2H, −C*H*_2_NMe_3_^+^Br^–^), 3.18 (s, 9H, –N­(C*H*_3_)_3_^+^Br^–^), 1.85 (m, 4H, –(C*H*_2_)_2_–), 1.49 (m, 2H, −C*H*_2_–), 1.41 (br., 6H, –(C*H*_2_)_3_–); ^13^C NMR (400 MHz, CD_3_OD) δ 170.35 (py-*C*OOH), 166.33 (*C*_4_–OH, pyridine), 149.64 (*C*_2_–*C*_6_, pyridine ring), 115.03 (*C*_3_–*C*_5_, pyridine ring), 70.97 (-pyO*C*H_2_–), 67.79 (−*C*H_2_NMe_3_^+^Br^–^), 53.81 (–N­(*C*H_3_)_3_^+^Br^–^), 29.76 (−*C*H_2_–), 29.72 (−*C*H_2_–), 29.31 (−*C*H_2_–), 26.91 (−*C*H_2_–), 26.41 (−*C*H_2_–), 23.71 (−*C*H_2_–). **L**_**10**_^**+** ^^1^H NMR (400 MHz, CD_3_OD) δ 7.82 (s, 2H, py.), 4.26 (t, *J* = 6.4 Hz, 2H, py-OC*H*_2_–), 3.42–3.35 (m, 2H, −C*H*_2_NMe_3_^+^Br^–^), 3.18 (s, 9H, –N­(C*H*_3_)_3_^+^Br^–^), 1.86 (d, *J* = 7.5 Hz, 4H, –(C*H*_2_)_2_–), 1.52 (p, *J* = 7.0 Hz, 2H, −C*H*_2_–), 1.46–1.28 (m, 10H, –(C*H*_2_)_5_–). ^13^C NMR (400 MHz, CD_3_OD) δ 168.89 (py-*C*OOH), 165.22 (*C*_4_–OH, pyridine), 149.00 (*C*_2_–*C*_6_, pyridine ring), 113.61 (*C*_3_–*C*_5_, pyridine ring), 69.52 (−pyO*C*H_2_−), 66.56 (−*C*H_2_NMe_3_^+^Br^–^), 52.41 (–N­(*C*H_3_)_3_^+^Br^–^), 29.05 (−*C*H_2_–), 29.03 (−*C*H_2_–), 28.88 (−*C*H_2_–), 28.79 (−*C*H_2_–), 28.33 (−*C*H_2_–), 25.94 (−*C*H_2_–), 25.48 (−*C*H_2_–), 22.60 (−*C*H_2_–).

#### Synthesis of Diethyl 4-(11-(methoxycarbonyl)­undecyloxy)­pyridine-2,6-dicarboxylate (**6**)

Diethyl 4-hydroxypyridine-2,6-dicarboxylate (**2**, 12 mmol, 3.0 g) was added to a suspension of anhydrous potassium carbonate (24 mmol, 3.5 g) in DMF (70 mL) and then methyl-11-bromoundecanoate (19 mmol, 4.6 mL) was slowly added. After stirring the mixture at 90 °C for 20 h, the solid was filtered off and the DMF was removed under vacuum. The residue was then purified thought column chromatography with hexane/ethyl acetate 1:2 as eluent to afford pure **6** (Rf: 0.73, 3.4 g, 65%);^73^ the low yield is due to the poor reaction conversion. ^1^H NMR (400 MHz, CDCl_3_) δ 7.76 (s, 2H, py.), 4.47 (q, *J* = 7.1 Hz, 4H, py-COOC*H*_2_CH_3_), 4.12 (t, *J* = 6.4 Hz, 2H, py-OC*H*_2_–), 3.66 (s, 3H, −COOC*H*_3_), 2.30 (t, *J* = 7.5 Hz, 2H, −C*H*_2_COOCH_3_), 1.88–1.77 (m, 2H, −C*H*_2_–), 1.63 (d, *J* = 7.2 Hz, 3H, −C*H*_2_–), 1.45 (t, *J* = 7.1 Hz, 6H, –(C*H*_2_)_3_–), 1.39–1.25 (m, 11H, −C*H*_2_– and py-COOCH_2_C*H*_3_).

#### Synthesis of 4-((11-Carboxyundecyl)­oxy)­pyridine-2,6-dicarboxylic Acid (**L**_***11***_^***–***^)

A solution of KOH (82.3 mmol, 4.6 g) in water (40 mL) was added to a solution of compound **6** (8.2 mmol, 3.6 g) in 100 mL of THF, and the mixture was stirred at room temperature for 16 h. THF was then removed and an aqueous HCl solution (0.1 M) was added dropwise until a pH value of 4 was reached. A viscous white precipitate was formed that was filtered off and dried in an oven at 70 °C to afford pure **L**_**11**_^**–**^ (3.1 g, 98%).^73^ ^1^H NMR (400 MHz, D2O) δ 7.52 (s, 2H, py.), 4.22 (t, *J* = 6.5 Hz, 2H, py-OC*H*_2_–), 2.17 (t, *J* = 7.5 Hz, 2H, −C*H*_2_–), 1.83 (p, *J* = 6.9 Hz, 2H, −C*H*_2_–), 1.50 (dt, *J* = 29.0, 7.5 Hz, 4H, –(C*H*_2_)_2_–), 1.32 (d, *J* = 12.1 Hz, 10H, –(C*H*_2_)_5_–); ^13^C NMR (400 MHz, D_2_O) δ 184.36 (−CH_2_ *C*OOH), 172.88 (py-*C*OOH), 166.83 (*C*_4_–OH, pyridine ring), 154.86 (*C*_2_–*C*_6_, pyridine ring), 111.38 (*C*_3_–*C*_5_, pyridine ring), 69.01­(O–CH_2_–CH_2_−), 37.67 (−*C*H_2_COOH), 28.76­(−*C*H_2_–), 28.65 (−*C*H_2_–), 28.61 (−*C*H_2_–), 28.55 (−*C*H_2_–), 28.42 (−*C*H_2_–), 28.04 (−*C*H_2_–), 25.93 (−*C*H_2_–), 25.06 (−*C*H_2_–).

#### Synthesis of Manganese Complexes **C**_**n**_^**i**^

A mixture of ligands **L**_**n**_^**i**^ (1.0 mmol) and Mn­(OAc)_2_·4H_2_O (1.0 mmol) in 50 mL of methanol was refluxed for 3 h. For complexes **C**_**H**_, **C**_**8**_, and **C**_**11**_^**–**^: after 3 h, the system was cooled and the white precipitate was filtered off and dried. For the complexes **C**_**8**_^**+**^, **C**_**10**_^**+**^: after 3 h, the system was cooled and diethyl ether was added to precipitate the complex as a white solid that was filtered and dried. The complexes were obtained with the following yields: **C**_**H**_: 0.249 g, 98%; **C**_**8**_: 0.348 g, 95%; **C**_**8**_^**+**^: 0.484 g, 96%; **C**_**10**_^**+**^: 0.516 g, 97%; **C**_**11**_^–^: 0.461 g, 95%. LC-ESI-MS: (**C**_**H**_) 184.02 [L + H]^+^, retention time (Rt) = 1.6 min; (**C**_**8**_) 296.15 [L + H]^+^, Rt = 14.7 min.; (**C**_**8**_^**+**^) 353.21 [L + H]^+^, Rt = 11.0 min; (**C**_**10**_^**+**^) 381.24 [L + H]^+^, Rt = 11.6 min; (**C**_**11**_^**–**^) 368.17 [L + H]^+^, Rt = 13.4 min. IR spectrum (cm^–1^): **C**_**H**_: 3453.79,2876.61, 2820.88, 1576.29, 1395.84, 1359.35, 1159.0, 1009.73, 933.43, 768.91, 713.84, 570.54. **C**_**8**_: 3310.49, 2923.05, 2852.73, 1654.58, 1590.89, 1564.35, 1456.88, 1417.07, 1381.25, 1338.79, 1309.60, 1118.53, 1040.25, 806.72, 721.80, 583.81. **C**_**8**_^**+**^: 3180.4, 3100.8, 2920.39, 2850.0, 1724.9, 1597.5, 1564.4, 1440.9, 1399.8, 1340.11, 1117.20, 1040.25, 908.89, 886.33, 808.05, 793.45, 732.42, 583.81. **C**_**10**_^**+**^: 3236.19, 2919.07, 2848.74, 1569.20, 1567.01, 1439.63, 1397.17, 1358.69, 1175.59, 1050.86, 899.60, 808.05, 744.36, 582.48. **C**_**11**_^–^: 3315.80, 2924.38, 2854.05, 1592.22, 1563.03, 1415.75, 1378.59, 1337.46, 1277.75, 1114.55, 1037.59, 963.29, 932.77, 885.0, 806.72, 793.45, 731.09, 583.81.

### Bleaching Activity

Preparation of the solutions: buffer solution (**A**) was prepared by dissolving sodium hydrogen carbonate (8.4 g, 0.10 mol) in water (1.0 L). The pH was then adjusted to the desired value by addition of sodium carbonate. A solution (**B**) of morin (7.0 mM) was prepared by dissolving morin hydrate (0.016 g, 0.050 mmol) in ethanol (8 mL). Solutions (**C**) of the catalysts (0.2 mM) were prepared by dissolving the complexes in an SDS (0.57 mmol, 11.4 mM) buffered solution (**A**, 50 mL). Catalytic solution (**D**) for oxidation of morin: in a volumetric flask (25 mL), an aliquot of the catalyst solution **C** (331 μL) was diluted in the appropriate buffer solution **A** (20 mL), and an aliquot of the morin solution (571 μL) was added with stirring. Then, the volume was adjusted to 25 mL adding solution **A**. The initial concentrations were as follows: [catalyst] = 2.65 μM, [morin] = 160 μM. Solution **D** (3 mL) was then added in a UV–vis cell, and 3 μL of an aqueous solution of H_2_O_2_ (50% w/w) was added to obtain a H_2_O_2_ concentration of 0.05% w/w. Progress of the reaction was monitored by following the reduction of the absorption at 402 nm (ε = 1.94 × 10^4^ M^–1^ cm^–1^). For the oxidation of Sunset Yellow FCF: a solution (**E**) of Sunset Yellow FCF (7.0 mM) was prepared by dissolving the azo-compound (0.025 g, 0.056 mmol) in ethanol (8 mL). Catalytic solution **F**: in a volumetric flask (25 mL), an aliquot of the catalyst solution **C** (331 μL) was diluted in an appropriate buffer solution **A** (20 mL), and an aliquot of the Sunset Yellow FCF solution (571 μL) was added with stirring. Then, the volume was adjusted to 25 mL adding solution **A**. The initial concentrations were as follows: [catalyst] = 2.65 μM, [Sunset Yellow FCF] = 160 μM. Solution **F** (3 mL) was then added in a UV–vis cell, and 3 μL of an aqueous solution of H_2_O_2_ (50% w/w) was added to obtain a H_2_O_2_ concentration of 0.05% w/w. Progress of the reaction was monitored by following the reduction of the absorption at 481 nm (ε = 1.99 × 10^4^ M^–1^ cm^–1^). The conversion is obtained with the formula

$$\mathrm{conversion}\;\left(\%\right)=\frac{A_{t_{0}}-A_{t_{i}}}{A_{t_{0}}}\times 100$$

where *A*_*t*0_ is the absorbance at time zero, while *A*_*ti*_ is the absorbance at time *i*. An example of the conversion calculation is provided in Figure S29.

The apparent TOF value is obtained by linear fitting of the kinetic curves in the range of 5–20 min. The TOF is calculated by the slope of the obtained lines, as in the formula

$$\mathrm{TOF}\;\left(h^{-1}\right)=\frac{\mathrm{slope}}{100}\times \frac{\left[\mathrm{morin}\right]}{\left[\mathrm{cat}\right]}\times 60$$

where the slope is expressed in min^–1^, and the concentrations are reported in μM.

## Conclusion

In this work, manganese­(II) complexes derived from chelidamic acid were synthesized and evaluated as catalysts for hydrogen peroxide activation in dye bleaching under mild aqueous conditions. The ligands were functionalized at the 4-position with alkyl chains of varying lengths (C_8_–C_10_) and different terminal groups (anionic, cationic, and neutral). These structural modifications were found to significantly influence catalytic performance. In particular, structural modifications to the ligand made it possible to tune the activity as a function of pH, yielding three highly active complexes (**C**_**8**_, **C**_**10**_^**+**^, and **C**_**11**_^**–**^) with activity peaks at different pH values (8.8, 9.8, and 9.3, respectively) depending on the charge on the alkyl chain (none, +, and −, respectively). This result provides a versatile set of catalysts, allowing the choice of one over another to be made based on the desired operating conditions. Overall, the substituted derivatives significantly outperform the parent hydroxyl ligand.

Kinetic investigations revealed the presence of an induction period, indicating that catalyst activation and the *in situ* formation of reactive oxidizing species are key steps governing the overall process. In this context, the bicarbonate buffer is suggested to play a noninnocent role, with a strong dependence of the catalytic activity on its concentration.

The catalytic performance was optimized by changing the parameters, such as, temperature, catalyst loading, and H_2_O_2_ concentrations, with optimal activity observed under mild conditions at a catalyst concentration of 2.65 μM (sub-ppm range) and an oxidant loading of 0.05% w/w. Significant activity was also retained at lower catalyst concentrations. Importantly, efficient bleaching was achieved at temperatures as low as 20–30 °C, highlighting the potential of these systems to reduce energy consumption in practical applications.

The versatility of the catalytic system was also expanded, proving to be effective in different oxidation reactions. The pollutant azo dye Sunset Yellow FCF was oxidized under the same mild conditions as the oxidation of morin. This catalytic system demonstrates its applicability to more recalcitrant substrates beyond those of model flavonoids.

Overall, the combination of catalytic efficiency, structural tunability, and operation under mild conditions makes these manganese complexes promising candidates for the development of next-generation, sustainable bleaching systems.

## Supplementary Material



## Notes

^1^H and ^13^C NMR spectra (Figures S1–S15), FT-IR spectra (Figures S16–S23), MS spectra (Figures S24–S28), and an example of morin conversion calculation (Figure S29) (PDF)
