# Supplementary material for: Boosting Peroxide Reactivity: New Mn(II) Chelidamate Catalysts for Efficient Low-Temperature Bleaching
Source: ACS Omega. 2026 Aug 7;11(32):48483–96. doi: 10.1021/acsomega.6c04886 (PMC13491884; doi:10.1021/acsomega.6c04886)
Supplement: Supplementary file 1 [file ao6c04886_si_001.pdf]

# Supporting information

# Boosting Peroxide Reactivity: New Mn(II) Chelidamate Catalysts for Efficient Low-Temperature Bleaching

*Immacolata Manco,[a] Vincenzo Langellotti,[a] Maria E. Cucciolito,[a, b] Carlo De Luca,[a] Roberto Esposito,[a, c,*] Massimo Melchiorre,[b, c] Gabriella Pinto,[a] , Matteo Lega,[d] Oreste Tarallo,[a] and Francesco Ruffo [a, b]*

[a] Dipartimento di Scienze Chimiche, Università di Napoli Federico II, Via Cintia 21, 80126, Napoli, Italy.

[b] Consorzio Interuniversitario di Reattività Chimica e Catalisi, Via Celso Ulpiani 27, 70126, Bari, Italy.

[c] ISusChem Srl, Piazza Carità 32, 80134, Napoli, Italy.

[d] Impresa Fater SpA, Via Mare Adriatico 122, 65010 Spoltore, Italia.

* roberto.esposito@unina.it

---

**25 Pages; 29 Figures**

# Table of Contents


NMR Spectra of prepared compounds ....................................................................................3

**Figure S1.** $^{1}H$ NMR spectrum of compound **2** in $CDCl_3$. ....................................................3

**Figure S2.** $^{1}H$ NMR spectrum of compound **3** in $CDCl_3$. ....................................................4

**Figure S3.** $^{1}H$ NMR spectrum of compound **$L_8$** in $D_2O$. ....................................................5

**Figure S4.** $^{13}C$ NMR spectrum of compound **$L_8$** in $D_2O$. ....................................................6

**Figure S5.** $^{1}H$ NMR spectrum of compound **4a** in $CDCl_3$. ....................................................7

**Figure S6.** $^{1}H$ NMR spectrum of compound **4b** in $CDCl_3$. ....................................................8

**Figure S7.** $^{1}H$ NMR spectrum of compound **5a** in $CDCl_3$. ....................................................9

**Figure S8.** $^{1}H$ NMR spectrum of compound **5b** in $CDCl_3$. ....................................................10

**Figure S9.** $^{1}H$ NMR spectrum of compound **$L_8^+$** in $CD_3OD$. ....................................................11

**Figure S10.** $^{13}C$ NMR spectrum of compound **$L_8^+$** in $CD_3OD$. ....................................................12

**Figure S11.** $^{1}H$ NMR spectrum of compound **$L_{10}^+$** in $CD_3OD$. ....................................................13

**Figure S12.** $^{13}C$ NMR spectrum of compound **$L_{10}^+$** in $CD_3OD$. ....................................................14

**Figure S13.** $^{1}H$ NMR spectrum of compound **6** in $CDCl_3$. ....................................................15

**Figure S14.** $^{1}H$ NMR spectrum of compound **$L_{11}^-$** in $D_2O$. ....................................................16

**Figure S15.** $^{13}C$ NMR spectrum of compound **$L_{11}^-$** in $D_2O$. ....................................................17

IR spectra of compounds ....................................................................................18

**Figure S16.** IR spectrum of compound **$L_H$**. ....................................................18

**Figure S17.** IR spectrum of compound **$L_8$**. ....................................................18

**Figure S18.** IR spectrum of compound **$L_8^+$**. ....................................................19

**Figure S19.** IR spectrum of compound **$L_{10}^+$**. ....................................................20

**Figure S20.** IR spectrum of compound **$C_H$**. ....................................................21

**Figure S21.** IR spectrum of compound **$C_8$**. ....................................................21

**Figure S22.** IR spectrum of compound **$C_8^+$**. ....................................................22

**Figure S23.** IR spectrum of compound **$C_{10}^+$**. ....................................................23

MS spectra of compounds ....................................................................................23

**Figure S24.** MS spectrum of compound **$C_H$**. ....................................................23

**Figure S25.** MS spectrum of compound **$C_8$**. ....................................................24

**Figure S26.** MS spectrum of compound **$C_8^+$**. ....................................................24

**Figure S27.** MS spectrum of compound **$C_{10}^+$**. ....................................................24

**Figure S28.** MS spectrum of compound **$C_{11}^-$**. ....25
Calculation of conversions and experimental setup ....25
**Figure S29.** Example of calculation of the conversions based on morin spectra. ....25
**Figure S30.** Experimental setup used for following the degradation of morin and Sunset Yellow FCF. ....26

# NMR Spectra of prepared compounds

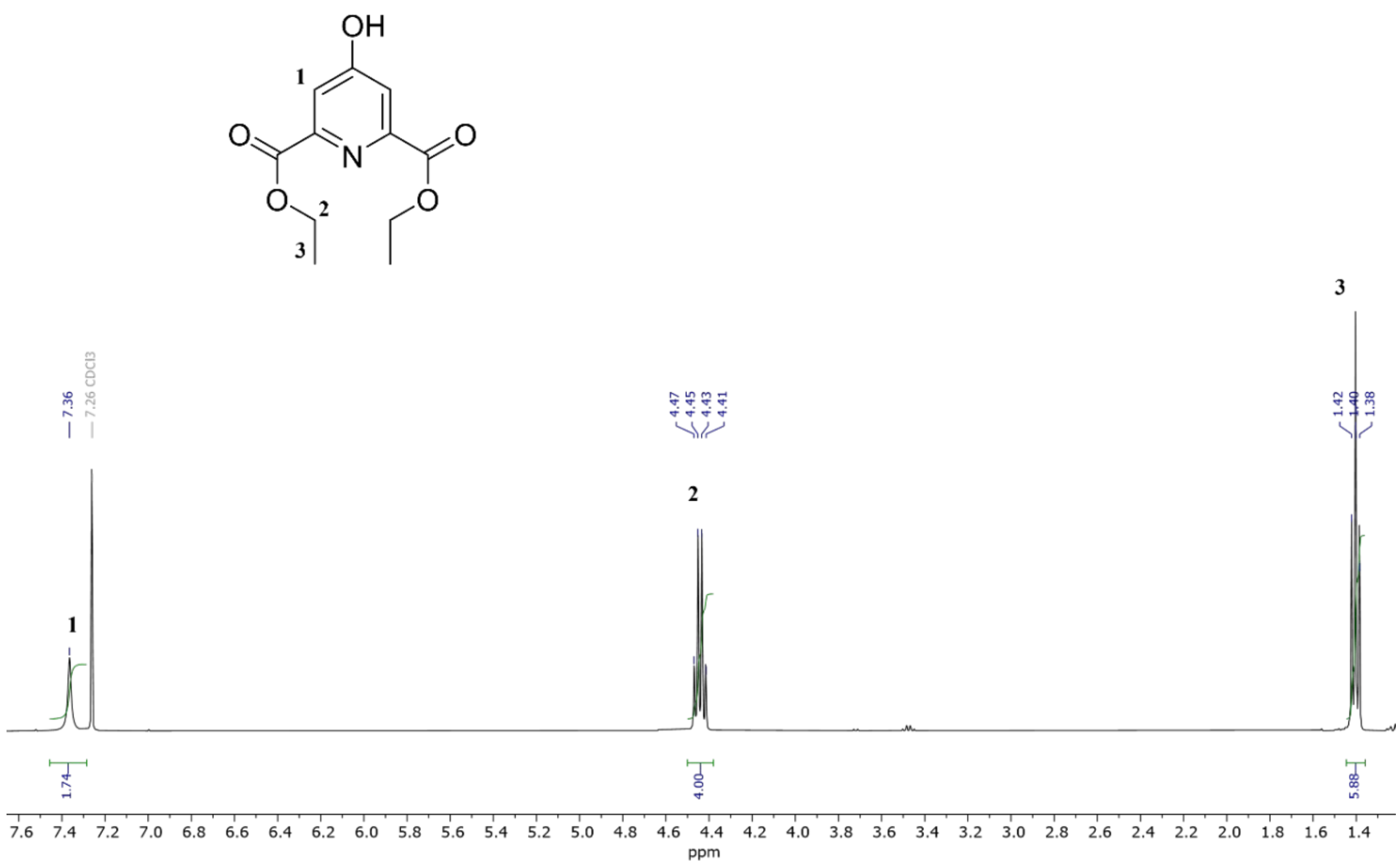

**Figure S1.** $^1$H NMR spectrum of compound **2** in $CDCl_3$.

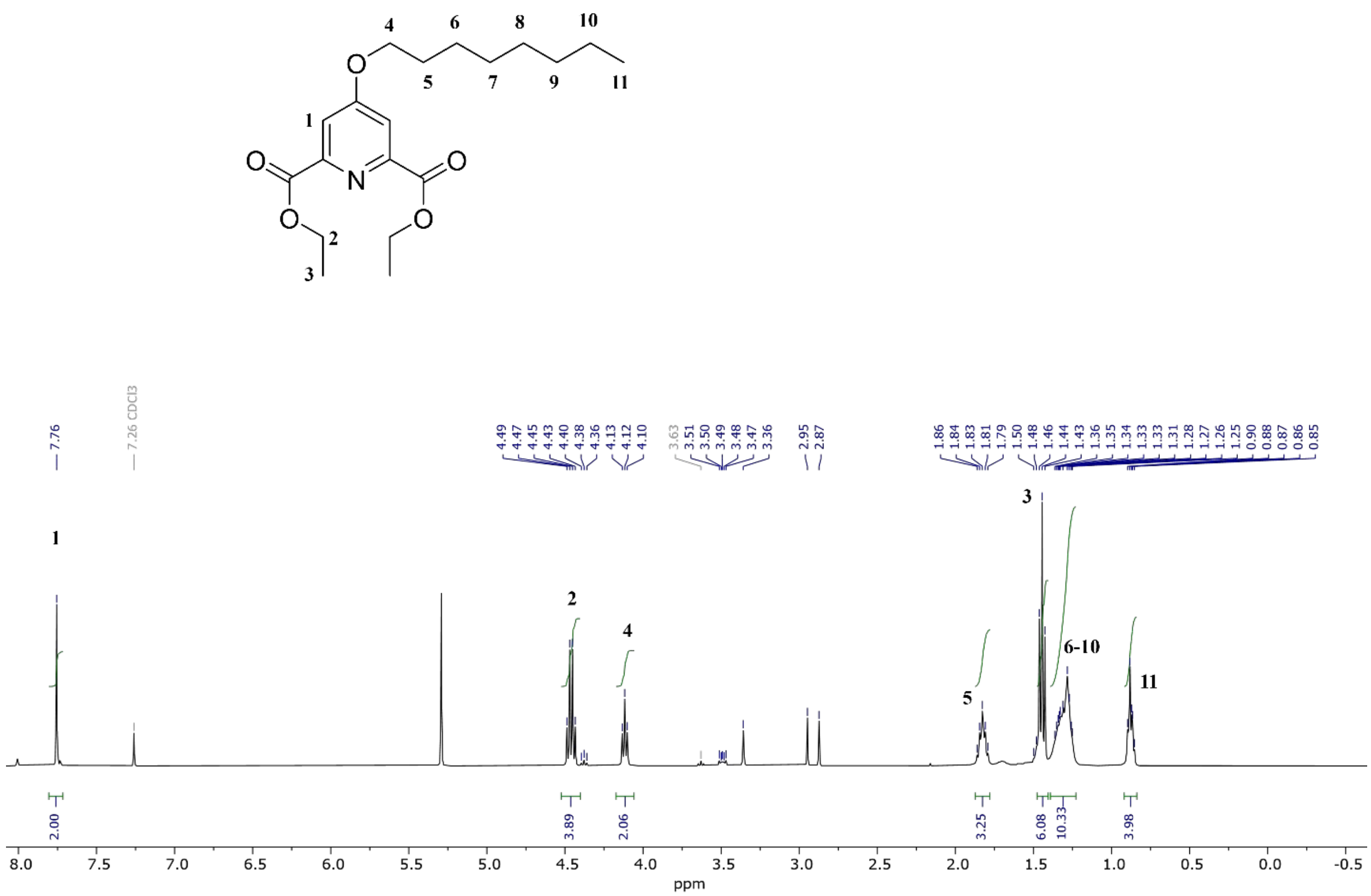

**Figure S2.** $^{1}H$ NMR spectrum of compound **3** in $CDCl_3$.

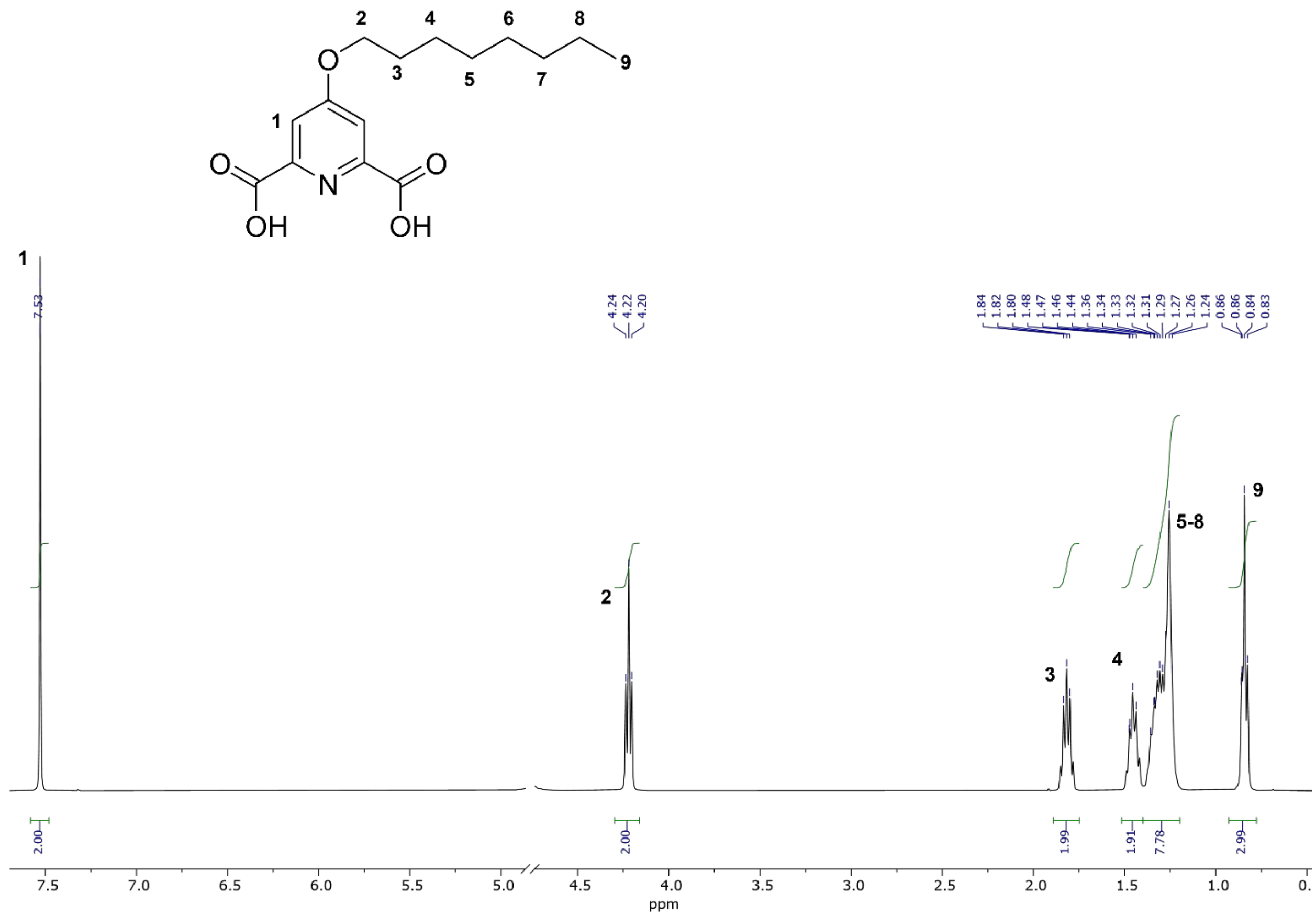


**Figure S3**. $^{1}H$ NMR spectrum of compound $\mathbf{L_8}$ in $D_2O$.

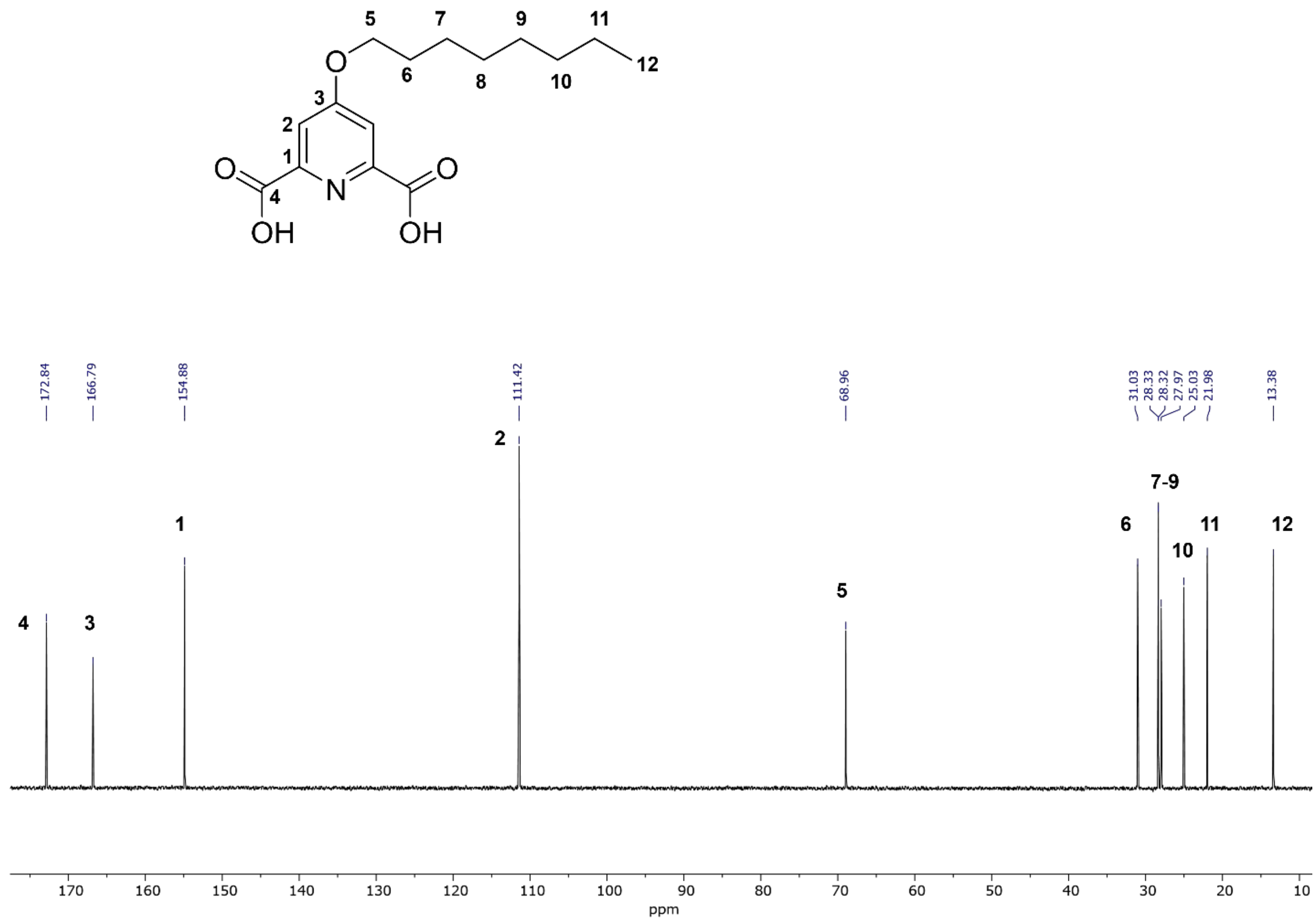


**Figure S4.** $^{13}$C NMR spectrum of compound **$L_8$** in $D_2O$.

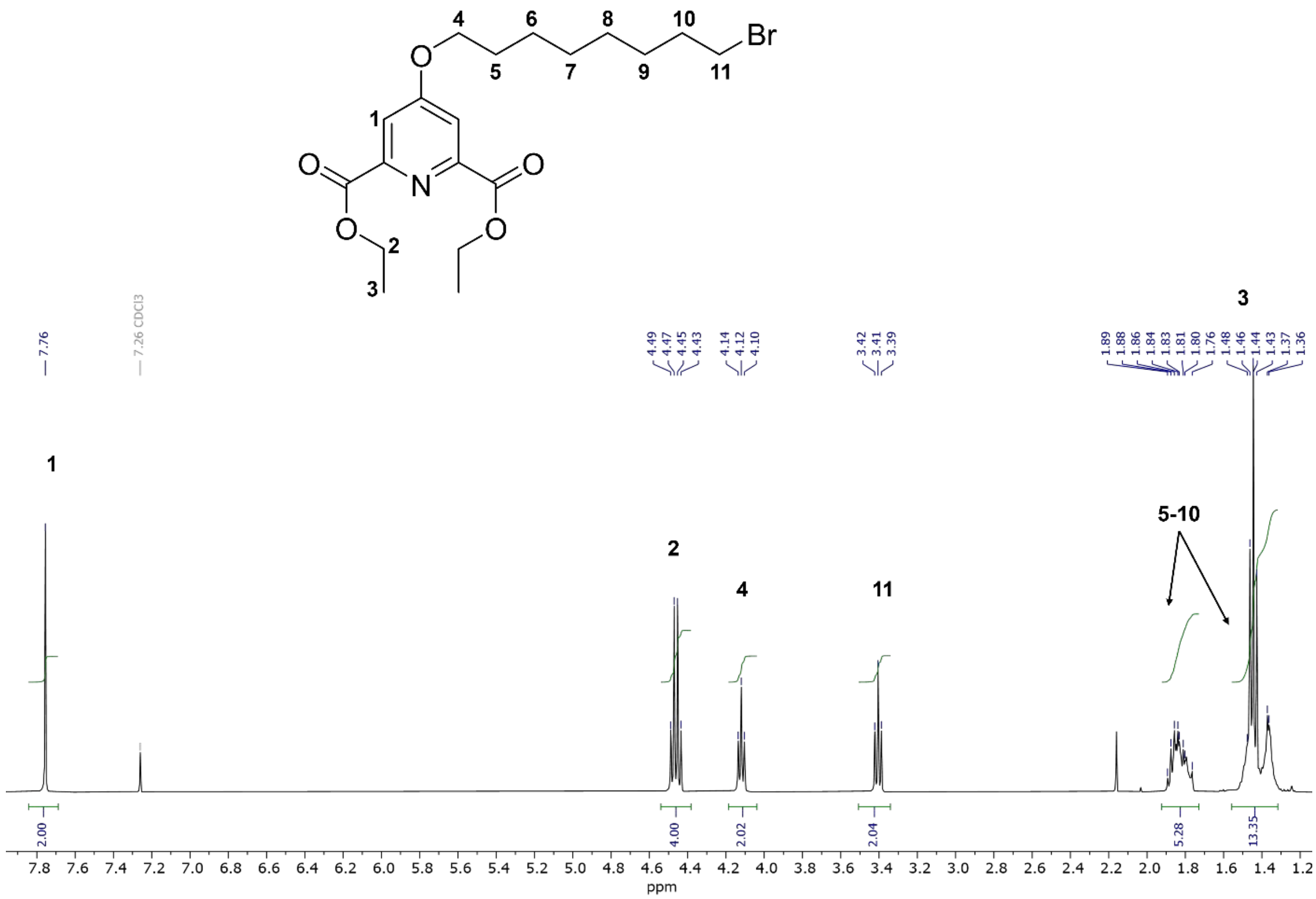


**Figure S5.** $^1$H NMR spectrum of compound **4a** in $CDCl_3$.

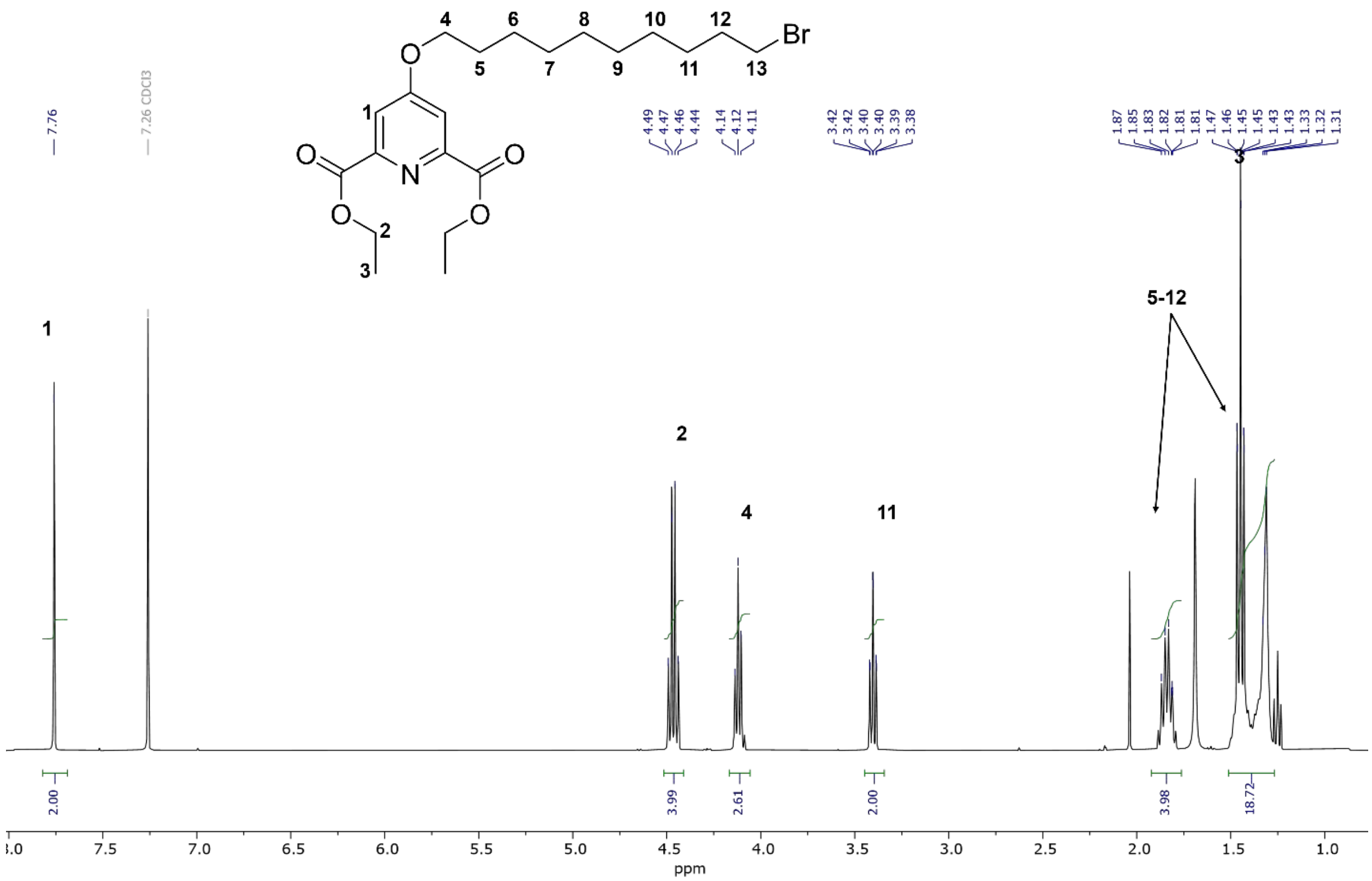

**Figure S6.** $^{1}H$ NMR spectrum of compound **4b** in $CDCl_3$.

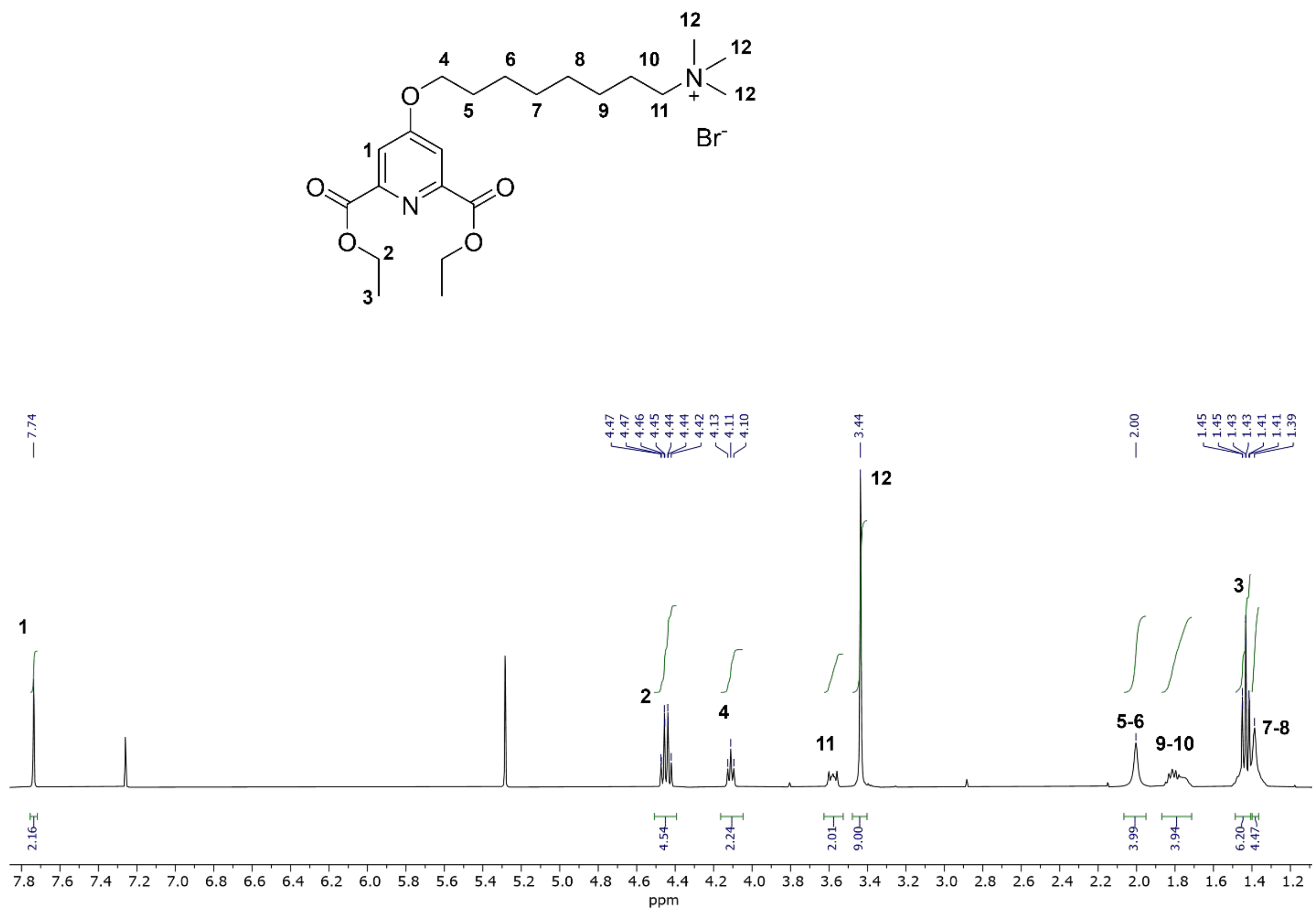


**Figure S7.** $^1H$ NMR spectrum of compound **5a** in $CDCl_3$.

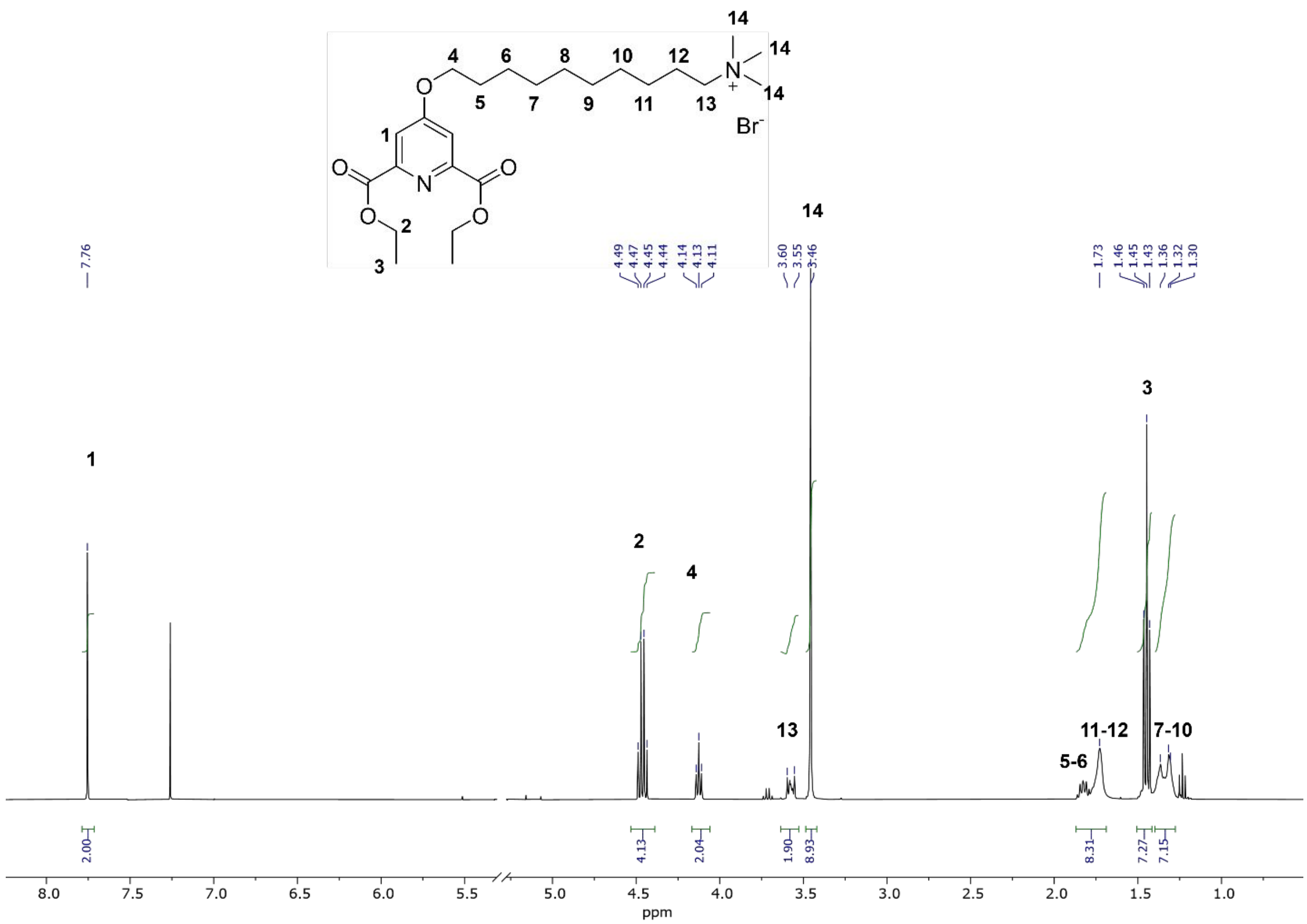


**Figure S8.** $^{1}H$ NMR spectrum of compound **5b** in $CDCl_3$.

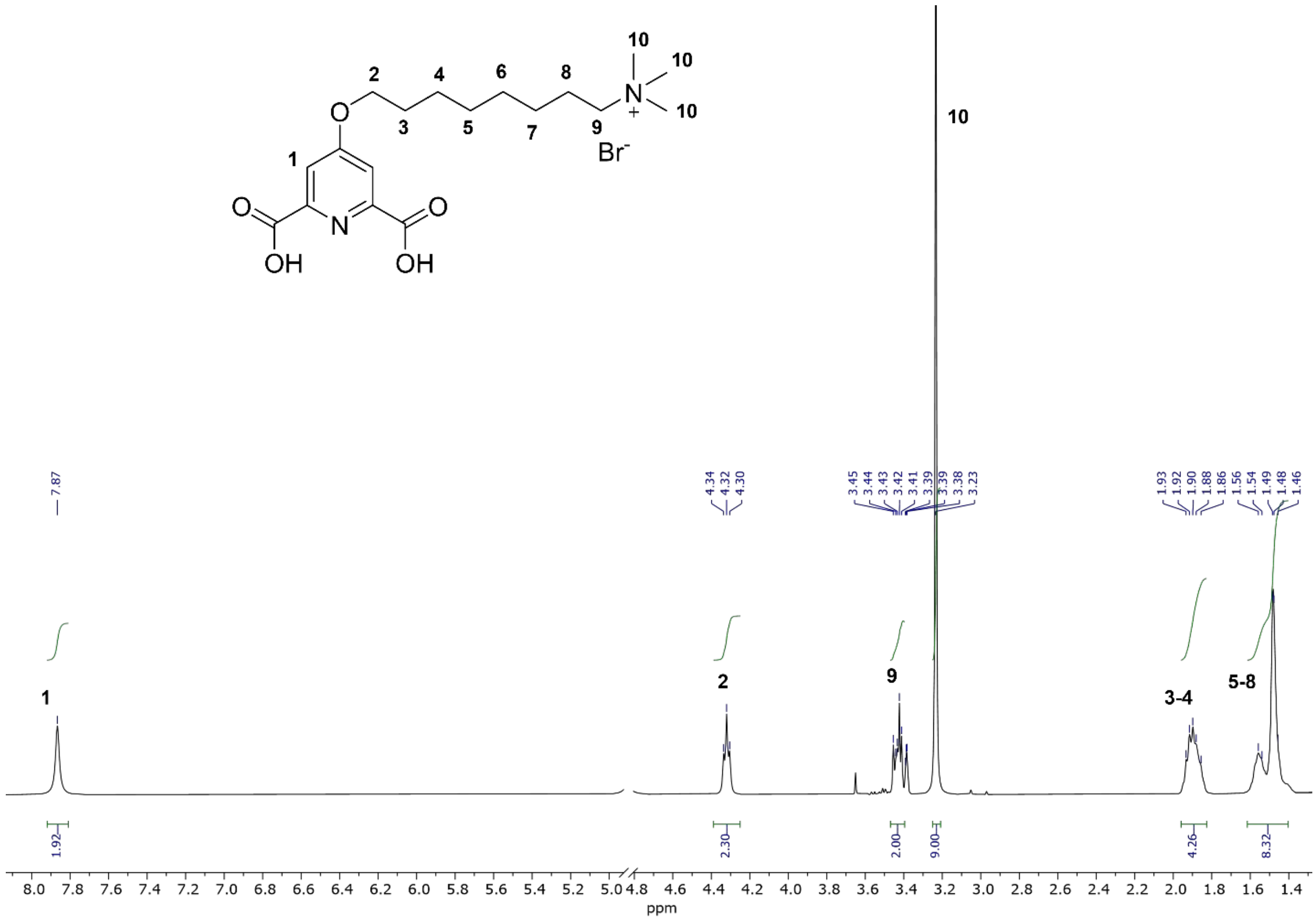


**Figure S9.** $^1$H NMR spectrum of compound $\mathbf{L_8^+}$ in $CD_3OD$.

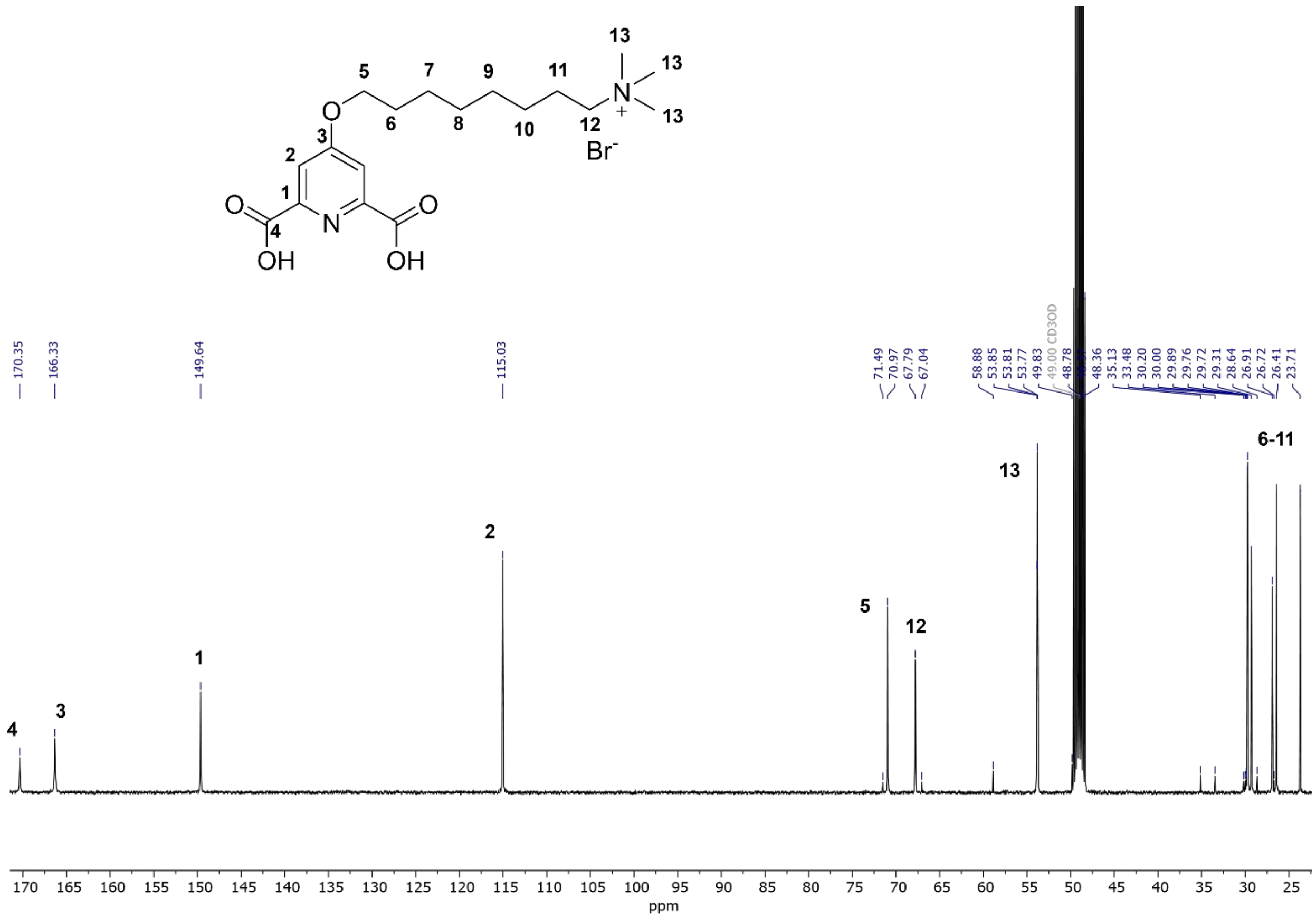


**Figure S10.** $^{13}C$ NMR spectrum of compound $L_8^+$ in $CD_3OD$.

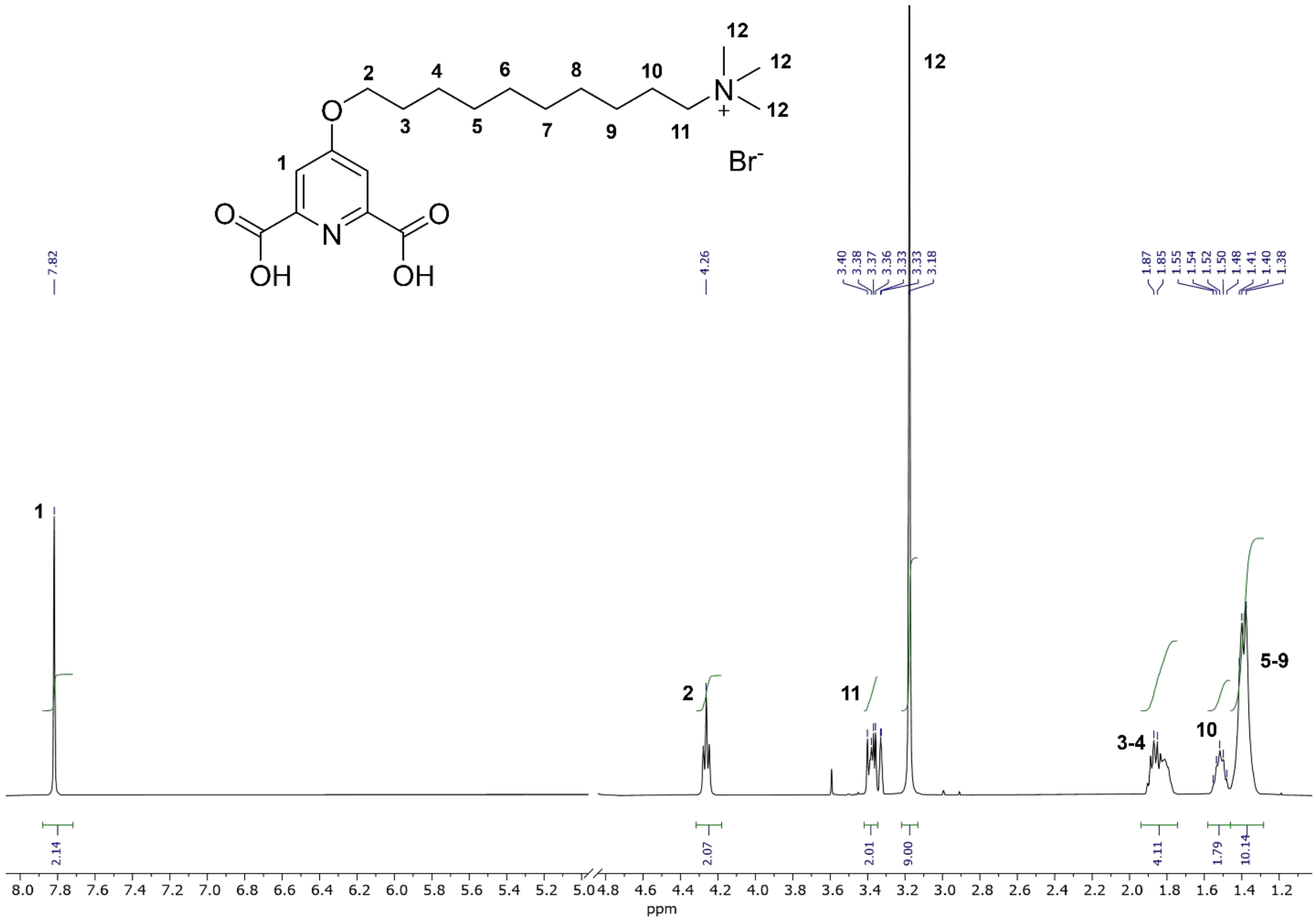


**Figure S11.** $^1H$ NMR spectrum of compound $\mathbf{L_{10}}^+$ in $CD_3OD$.

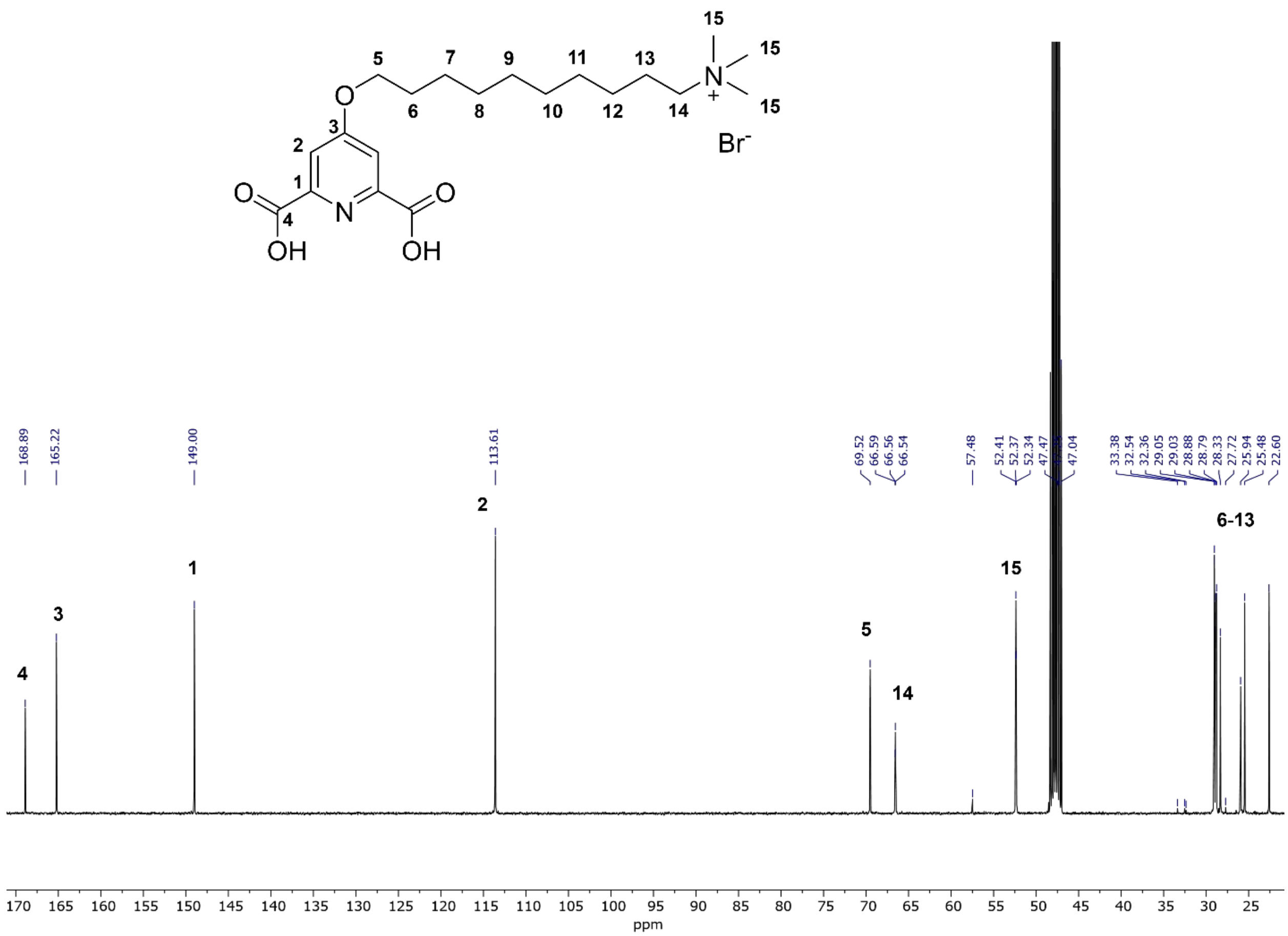


**Figure S12.** $^{13}$C NMR spectrum of compound $\mathbf{L_{10}}^+$ in $CD_3OD$.

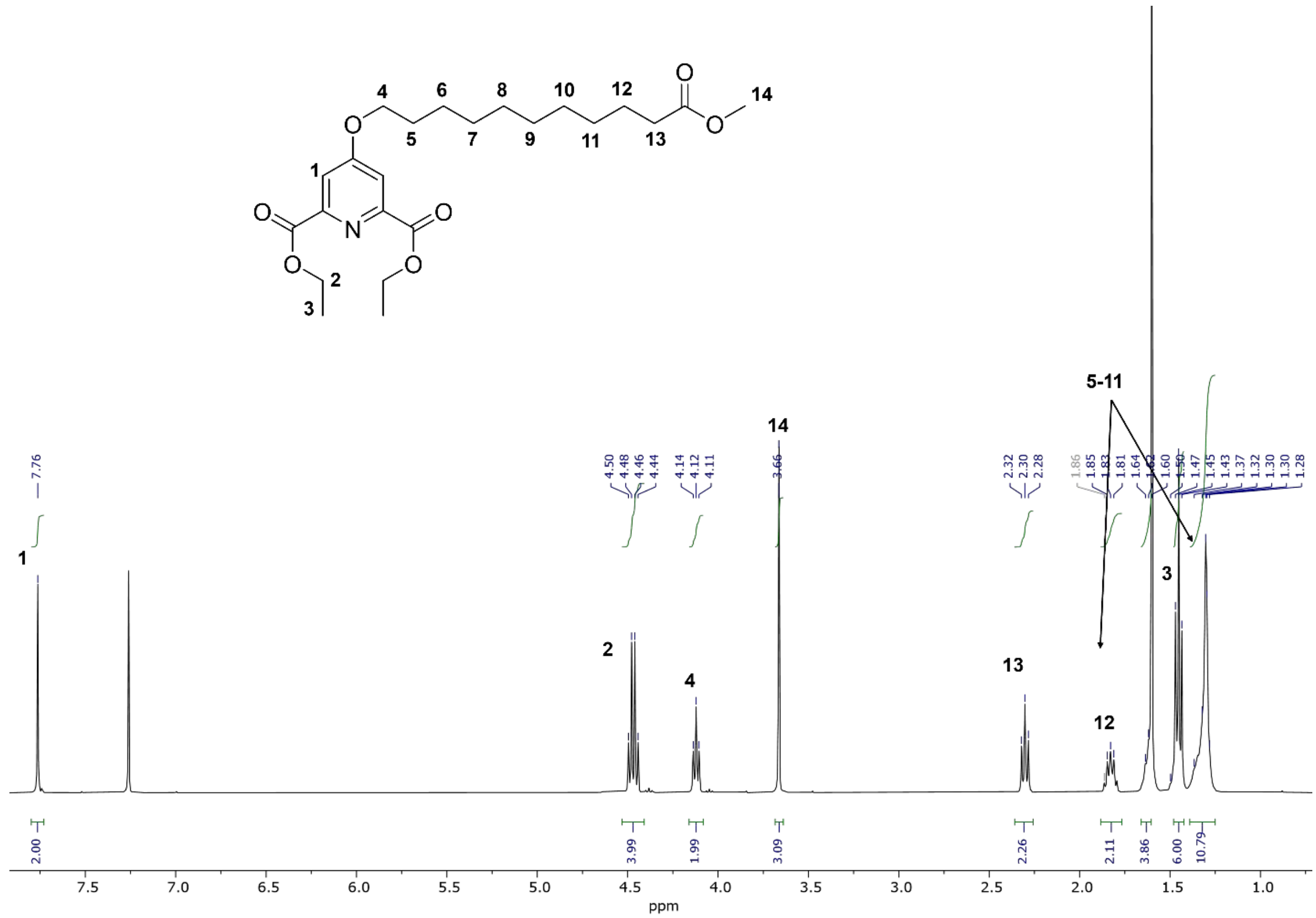


**Figure S13.** $^{1}H$ NMR spectrum of compound **6** in $CDCl_3$.

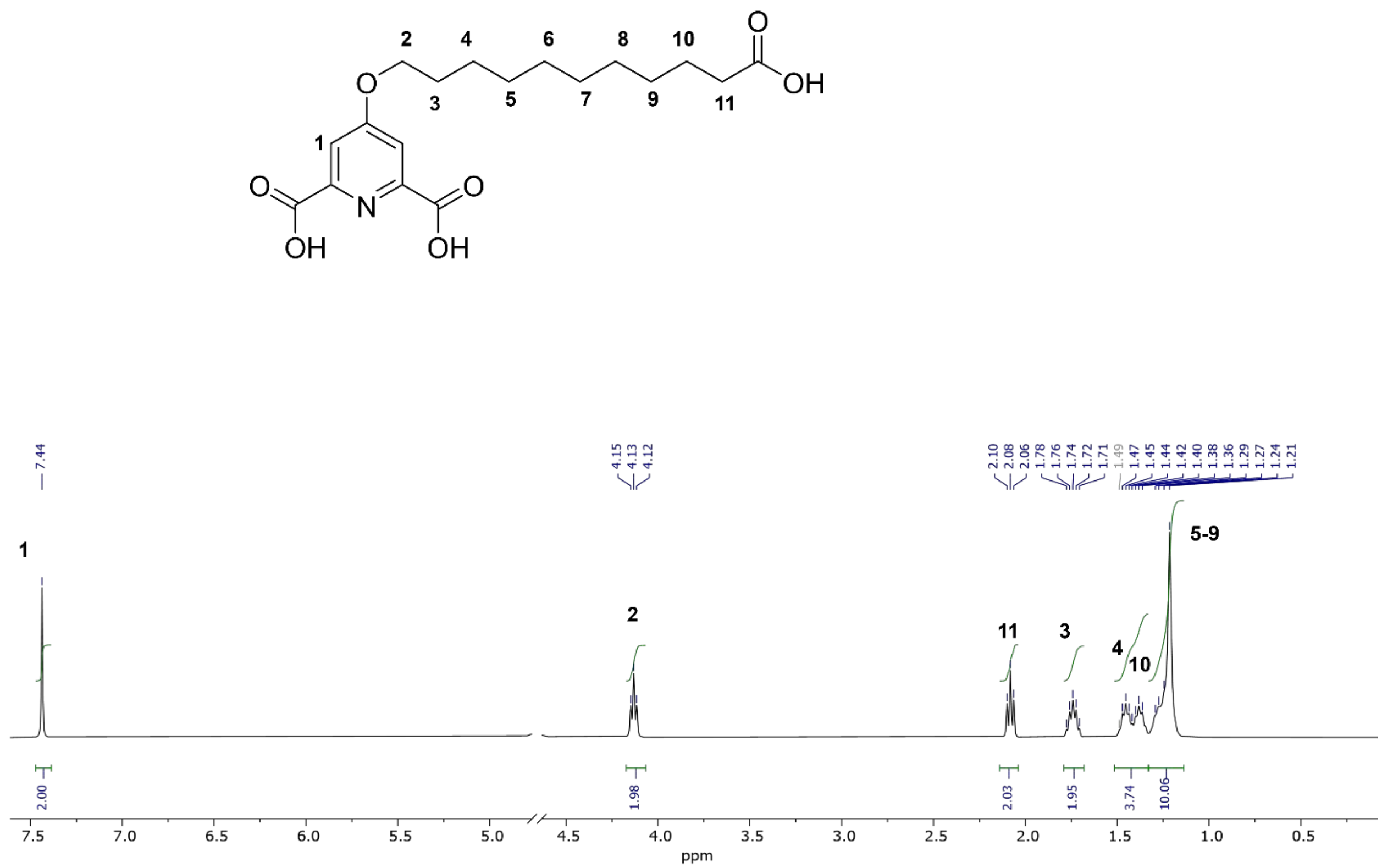

**Figure S14.** $^{1}$H NMR spectrum of compound $\mathbf{L_{11}}^{-}$ in $D_2O$.

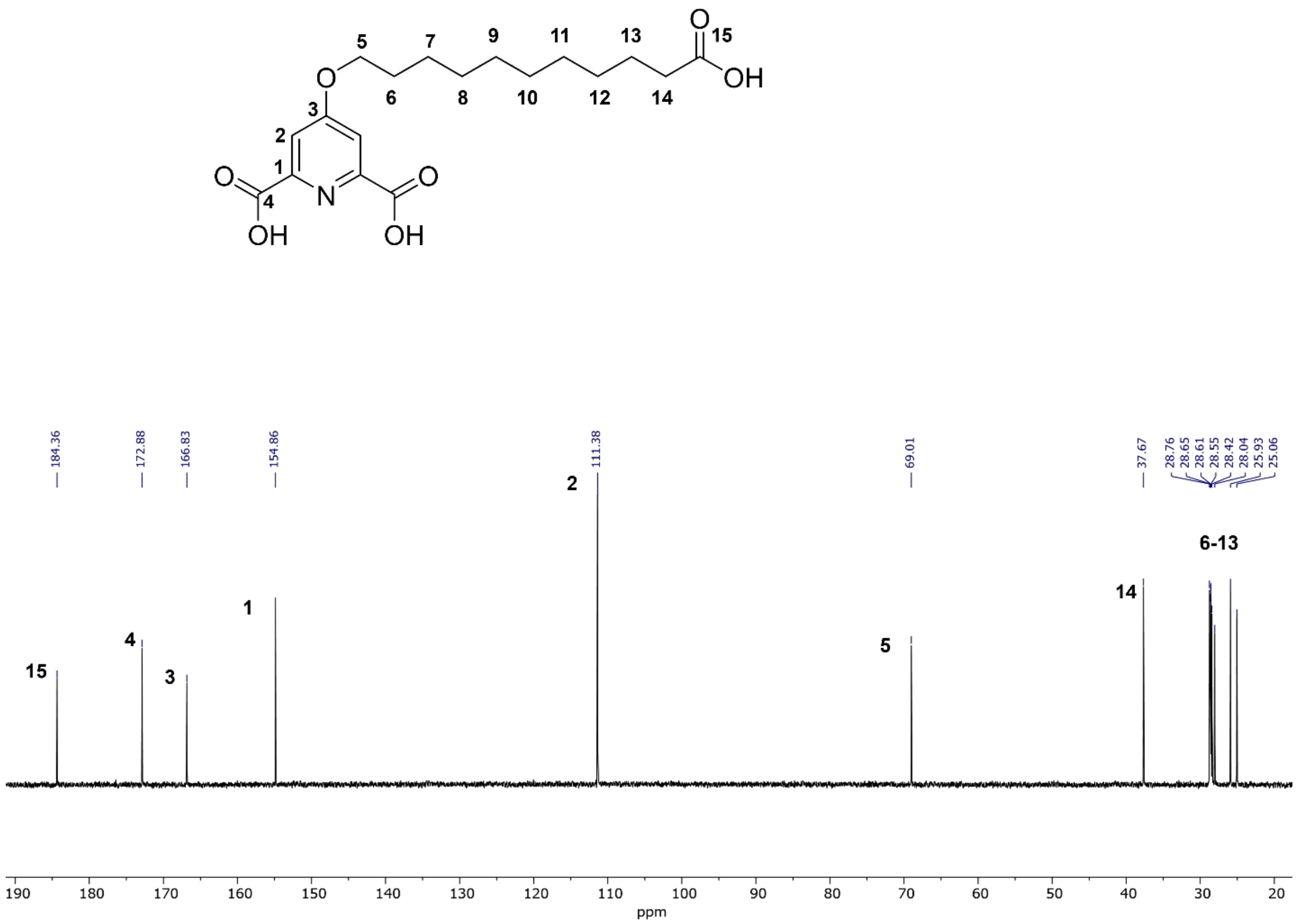


**Figure S15.** $^{13}C$ NMR spectrum of compound $\mathbf{L_{11}}^-$ in $D_2O$.

# IR spectra of compounds

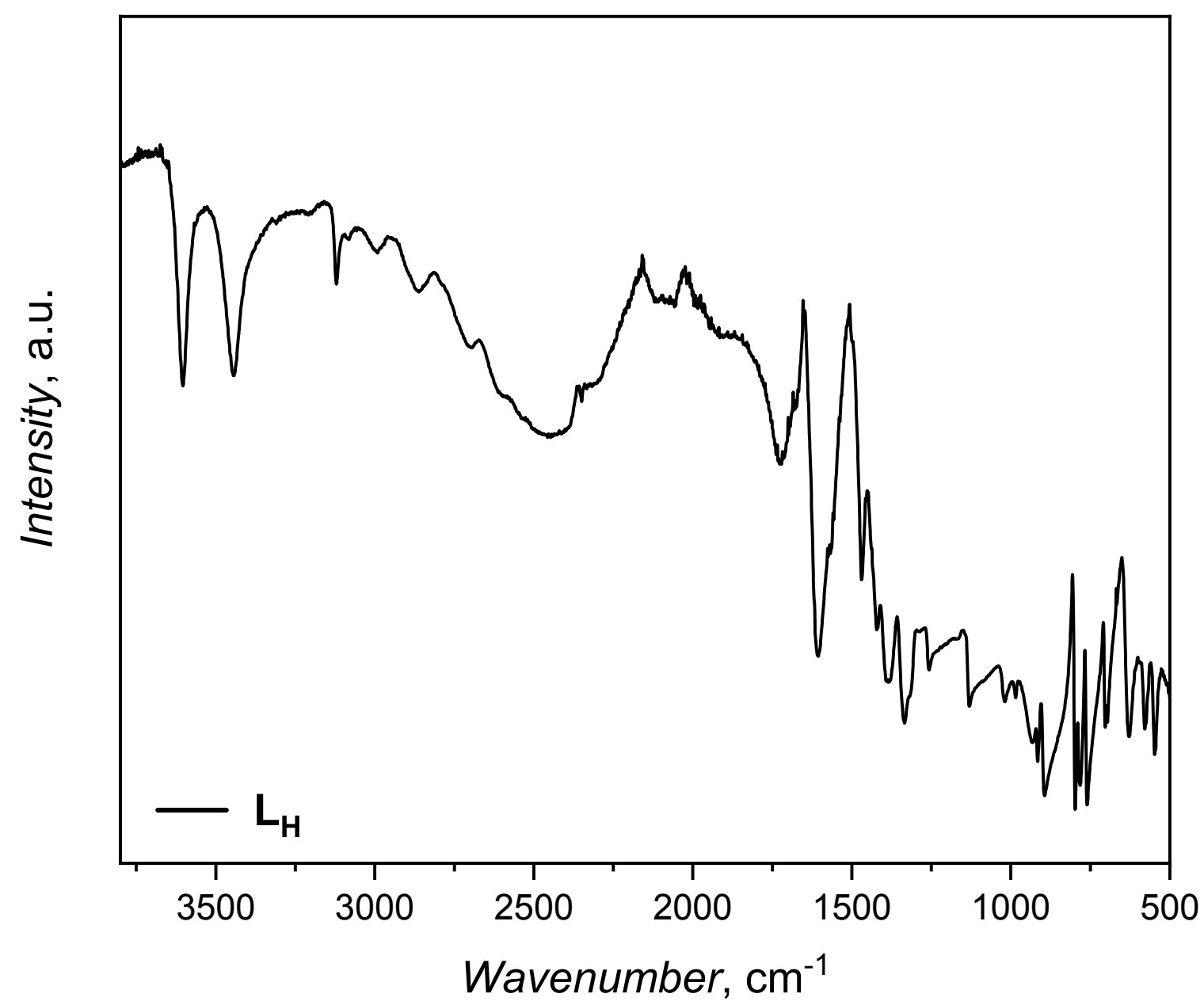


**Figure S16.** IR spectrum of compound $\mathbf{L_H}$.

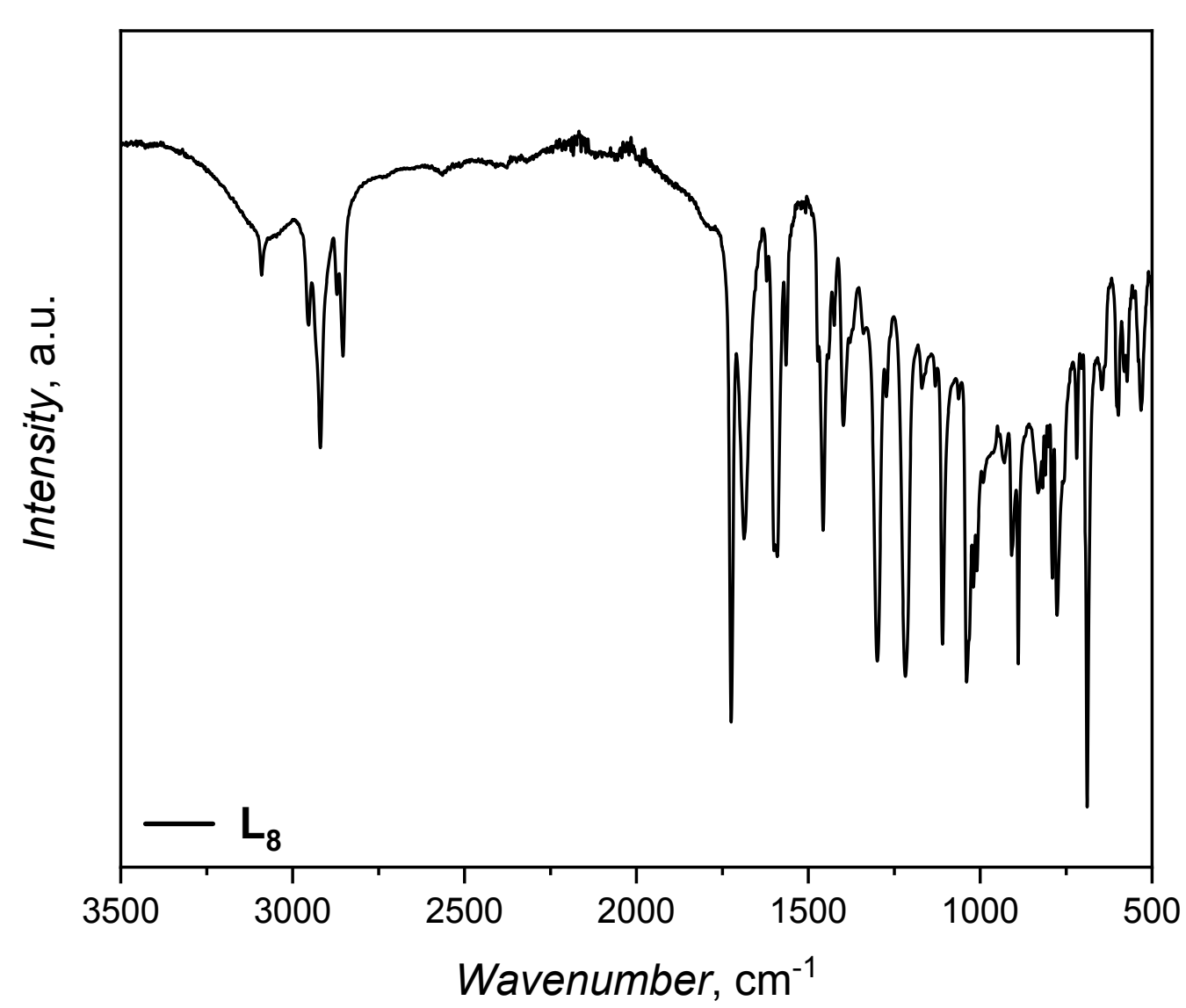


**Figure S17.** IR spectrum of compound $\mathbf{L_8}$.

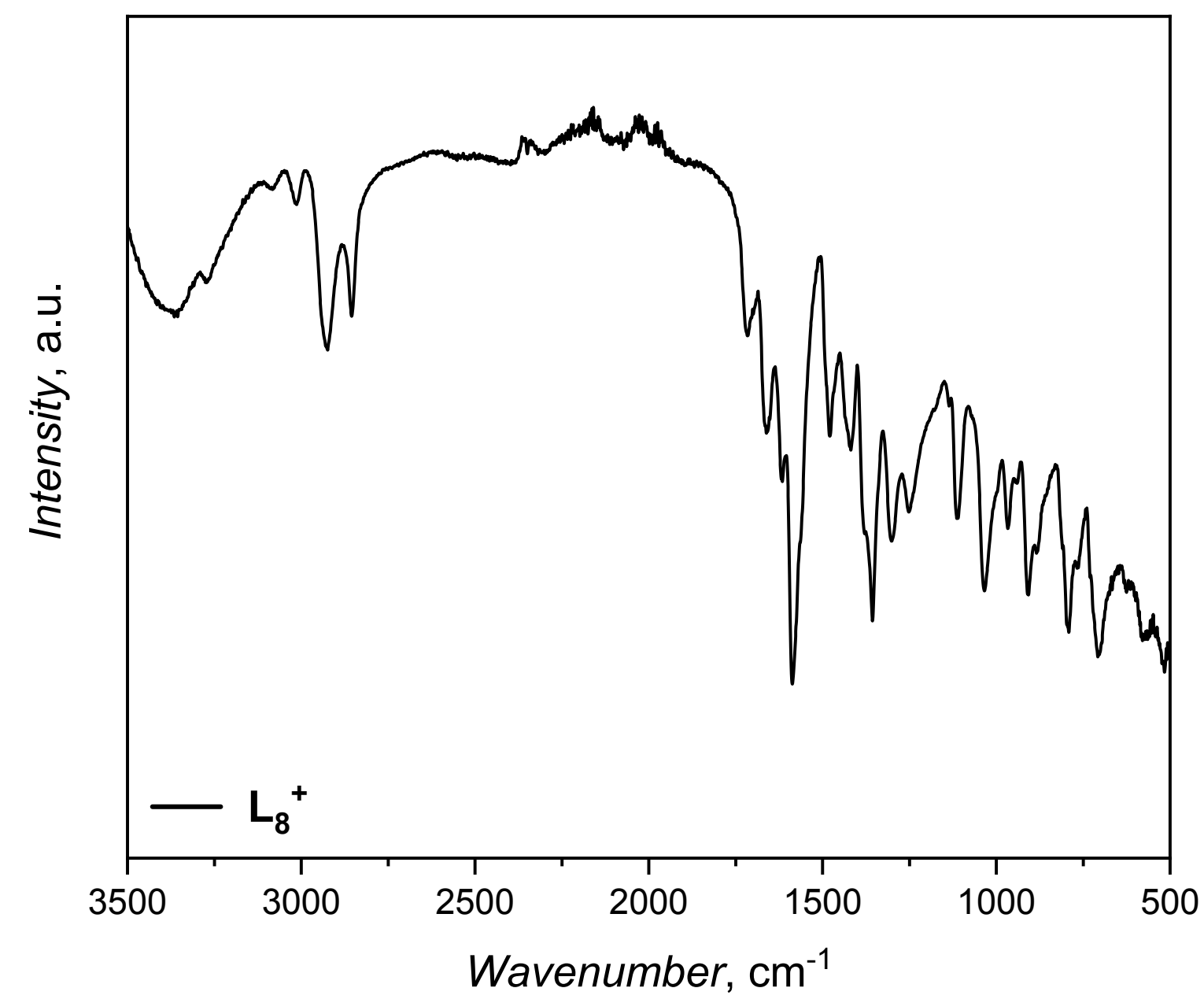


**Figure S18.** IR spectrum of compound $L_8^+$.

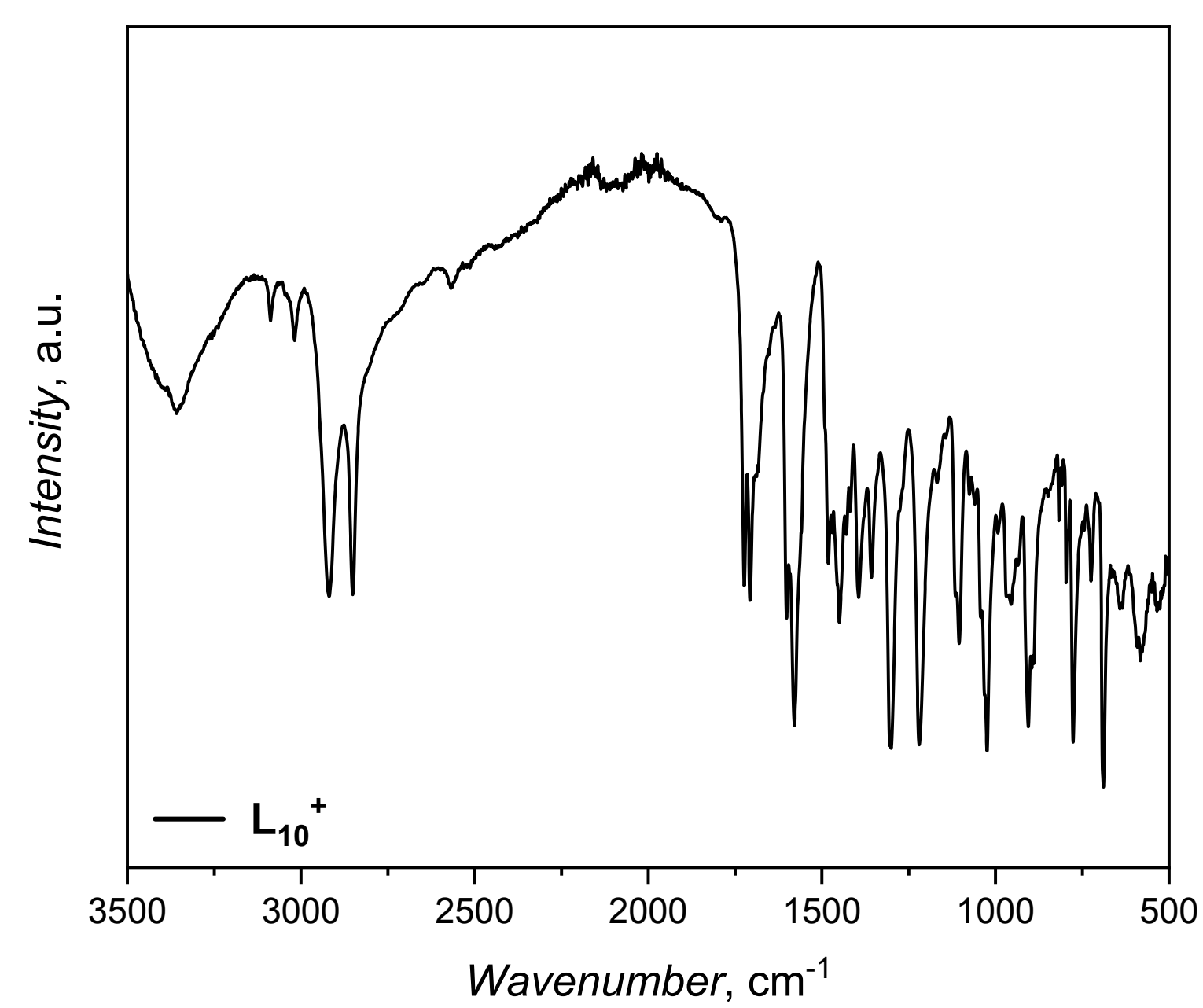

**Figure S19**. IR spectrum of compound $\mathbf{L_{10}}^{+}$.

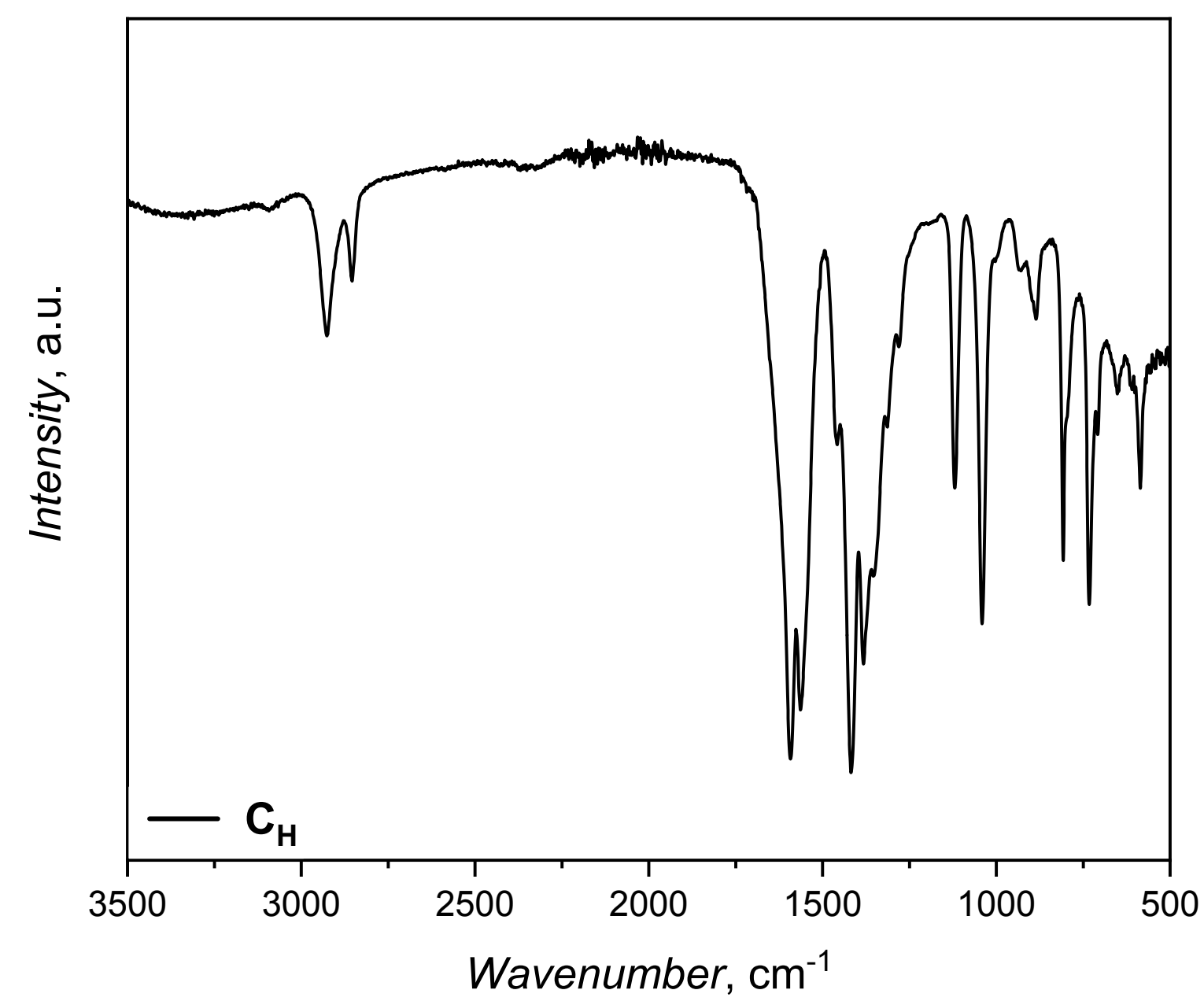

**Figure S20.** IR spectrum of compound $\mathbf{C_{H.}}$

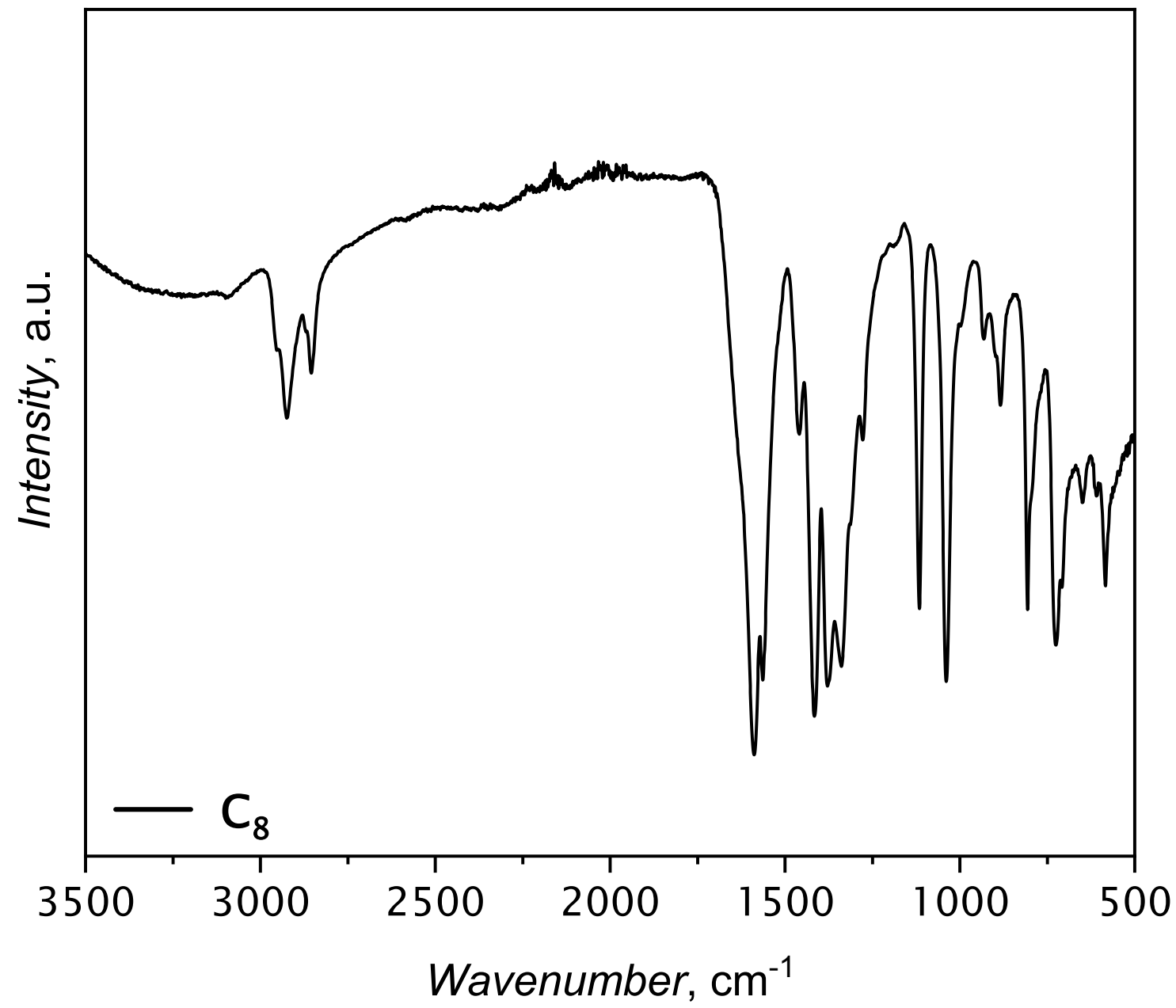


**Figure S21.** IR spectrum of compound $\mathbf{C_{8.}}$

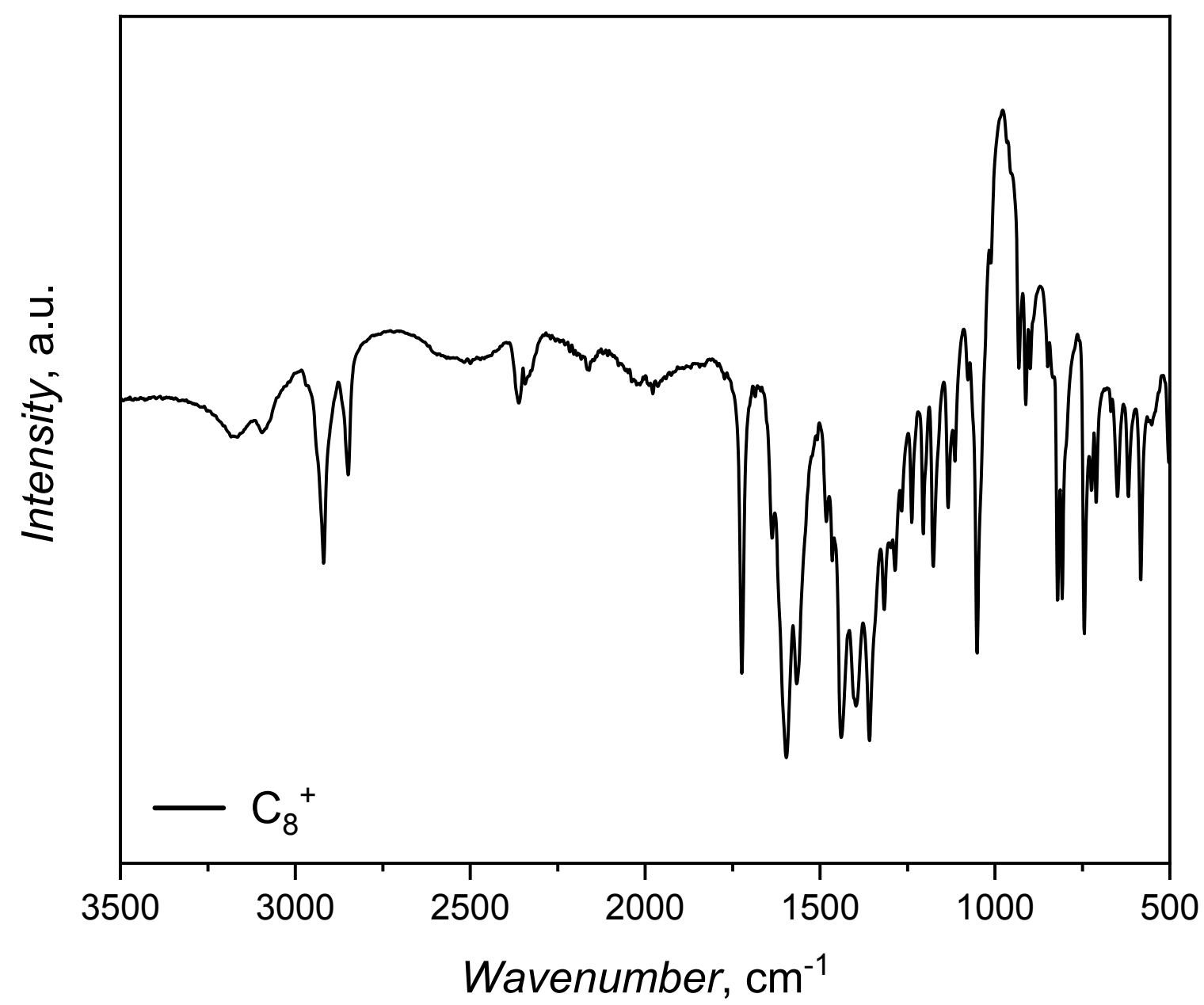


**Figure S22.** IR spectrum of compound $\mathbf{C_8^+}$.

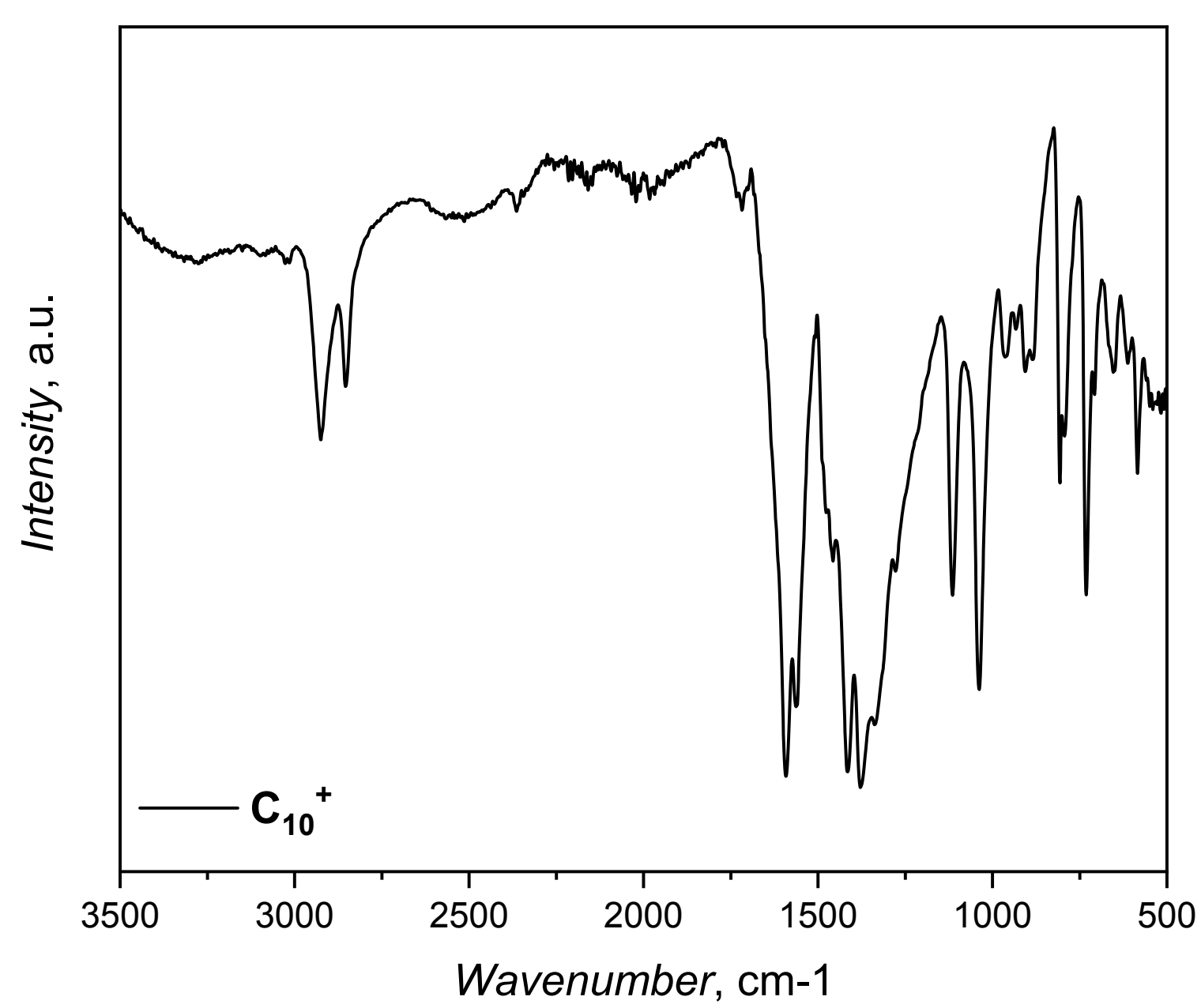

**Figure S23.** IR spectrum of compound $\mathbf{C_{10}^{+}}$.

# MS spectra of compounds

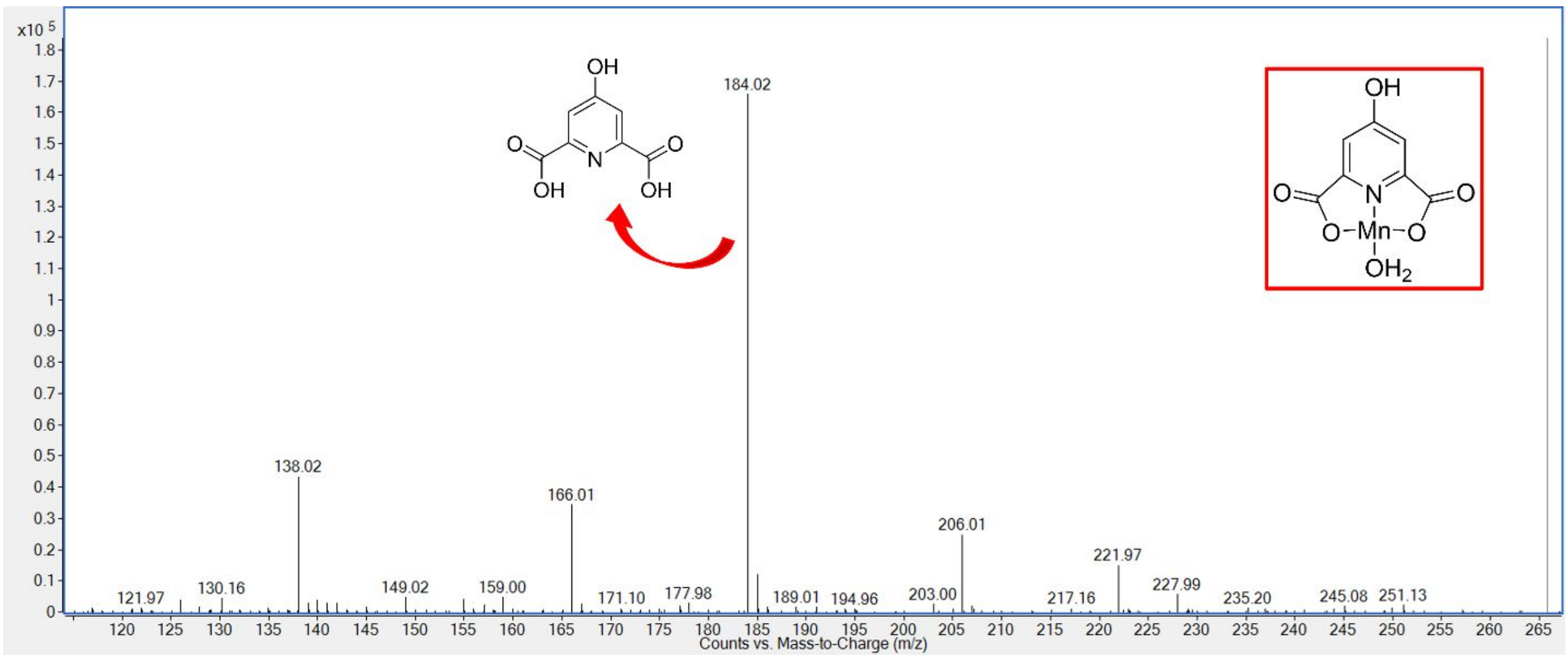


**Figure S24.** MS spectrum of compound $\mathbf{C_{H}}$.

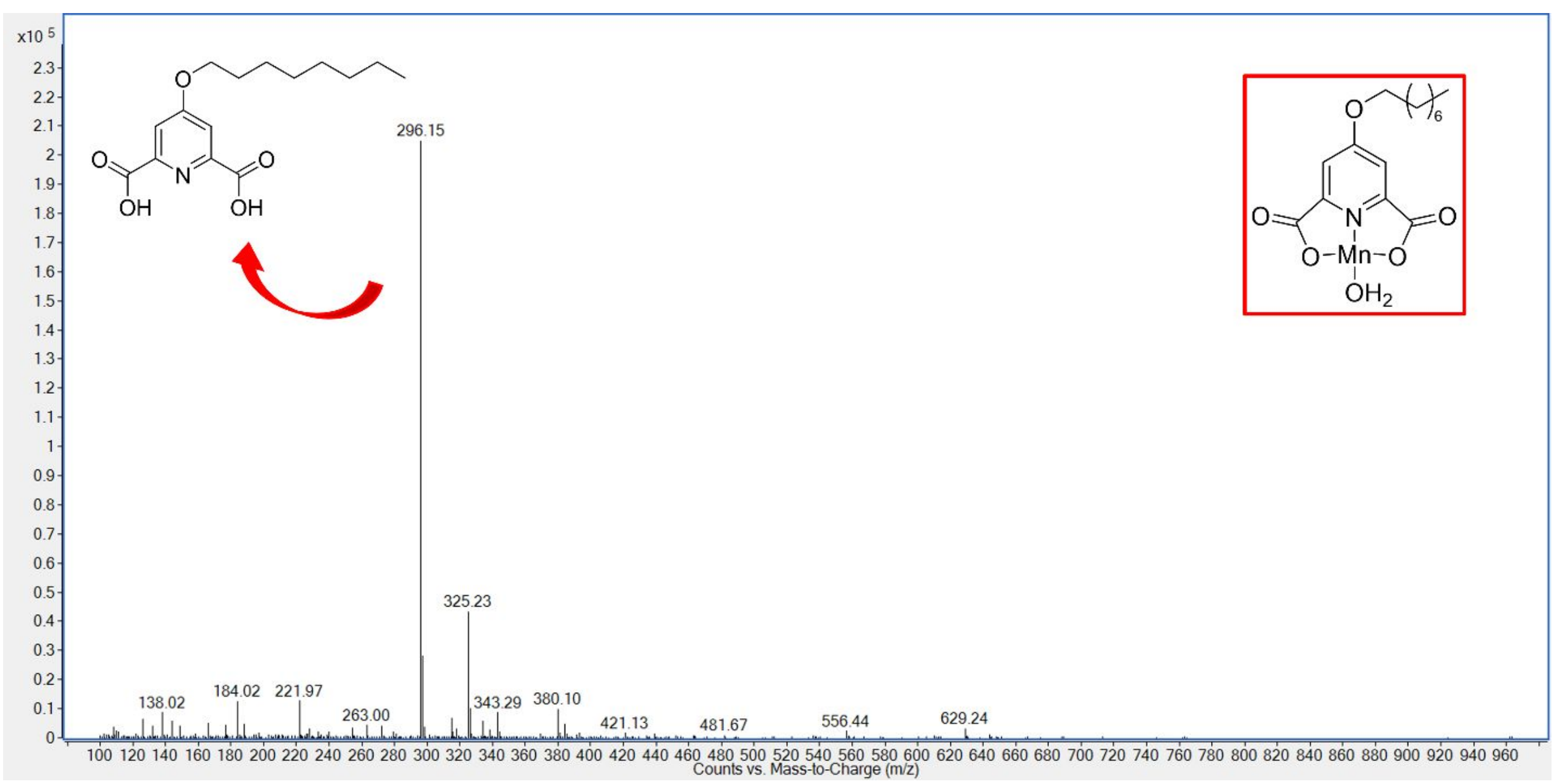

**Figure S25.** MS spectrum of compound $\mathbf{C_8}$.

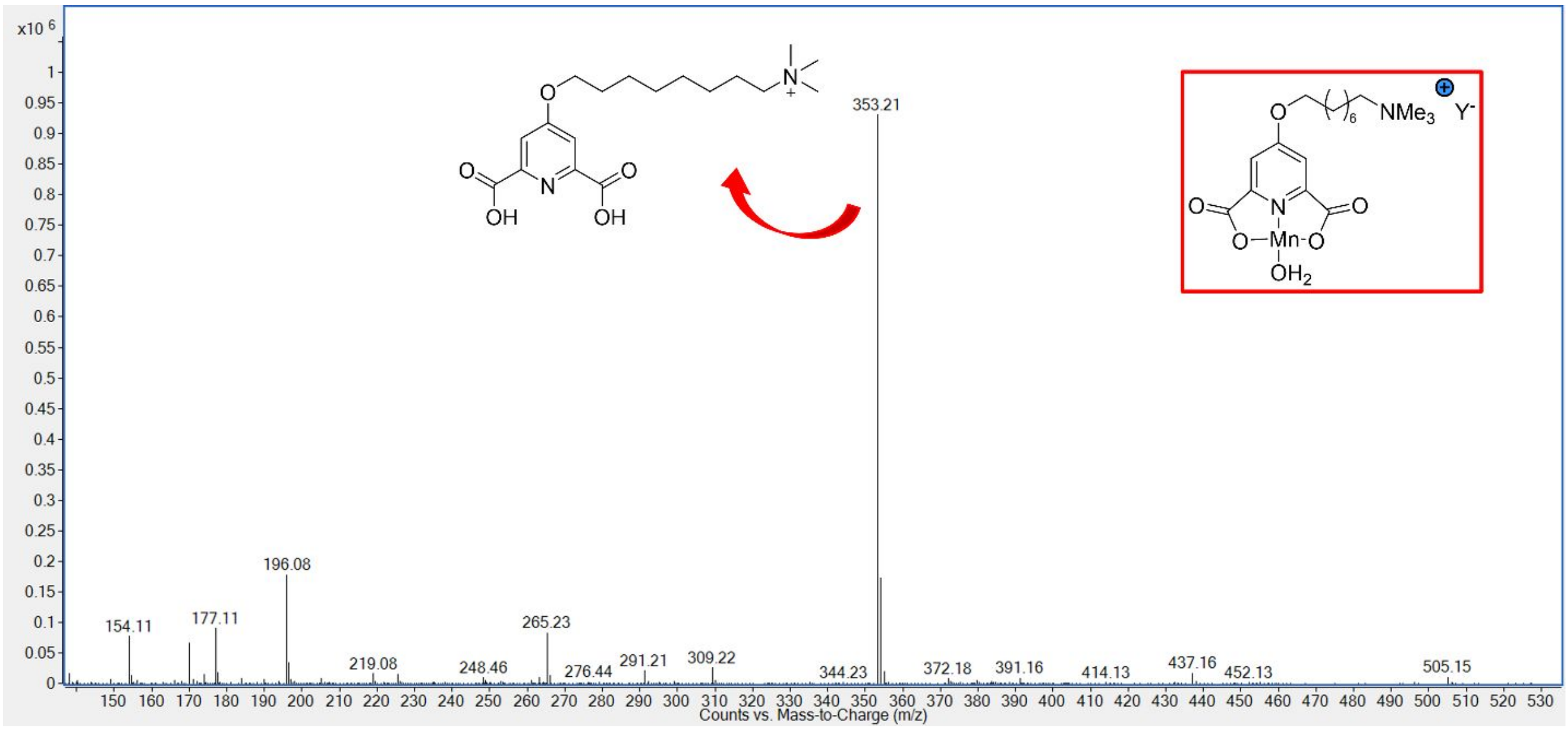


**Figure S26.** MS spectrum of compound $\mathbf{C_8}^+$.

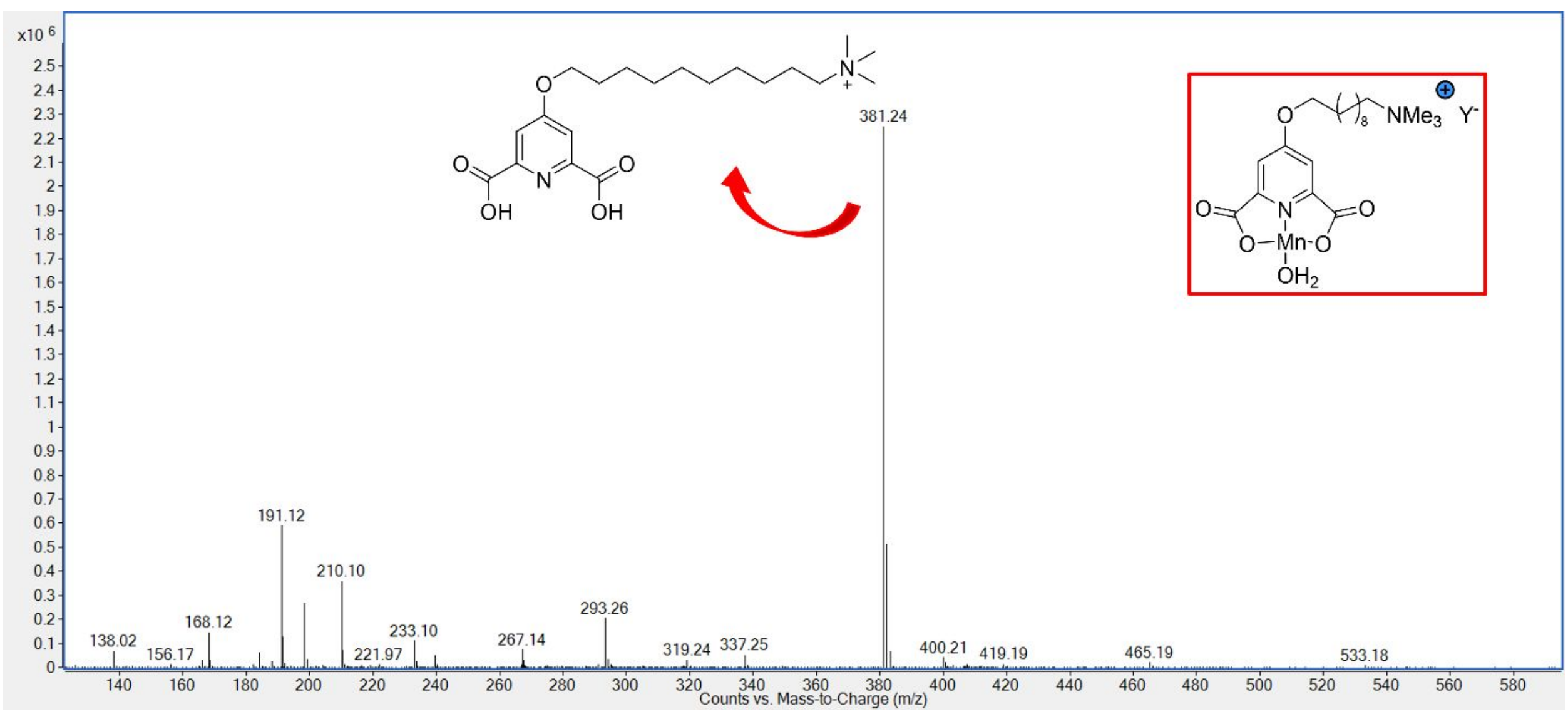


**Figure S27.** MS spectrum of compound $\mathbf{C_{10}}^+$.

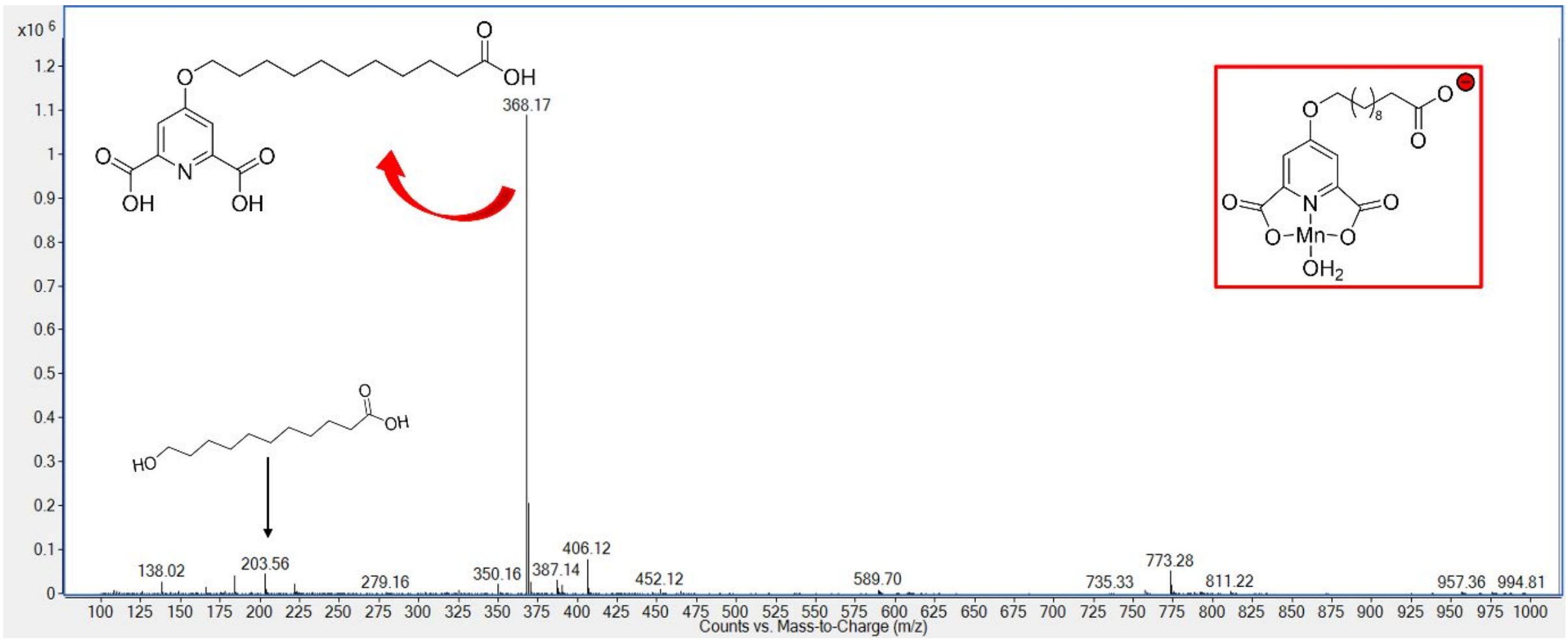


**Figure S28.** MS spectrum of compound $\mathbf{C_{11}^{-}}$.

# Calculation of conversions and experimental setup

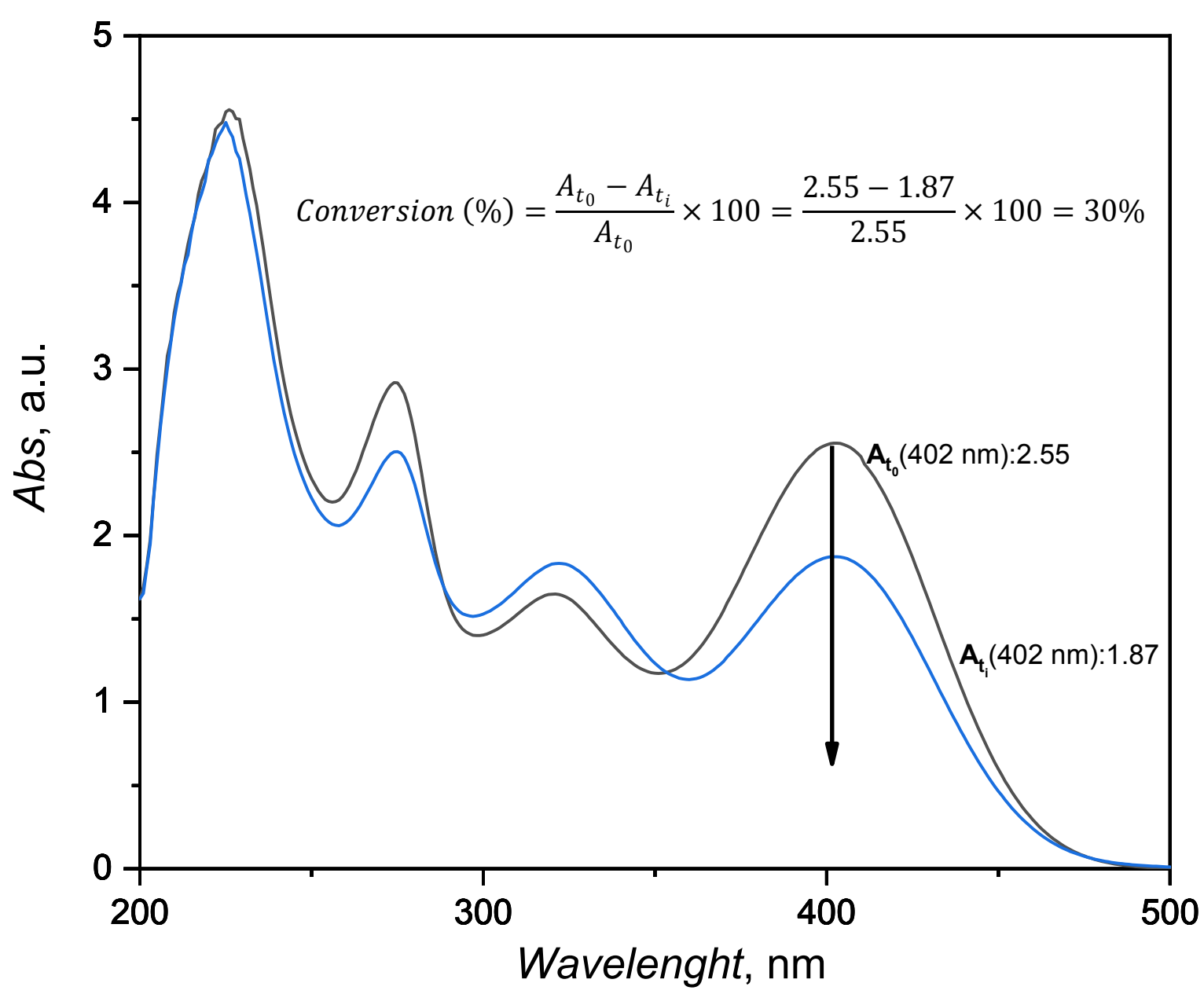


**Figure S29.** Example of calculation of the conversions based on morin spectra.

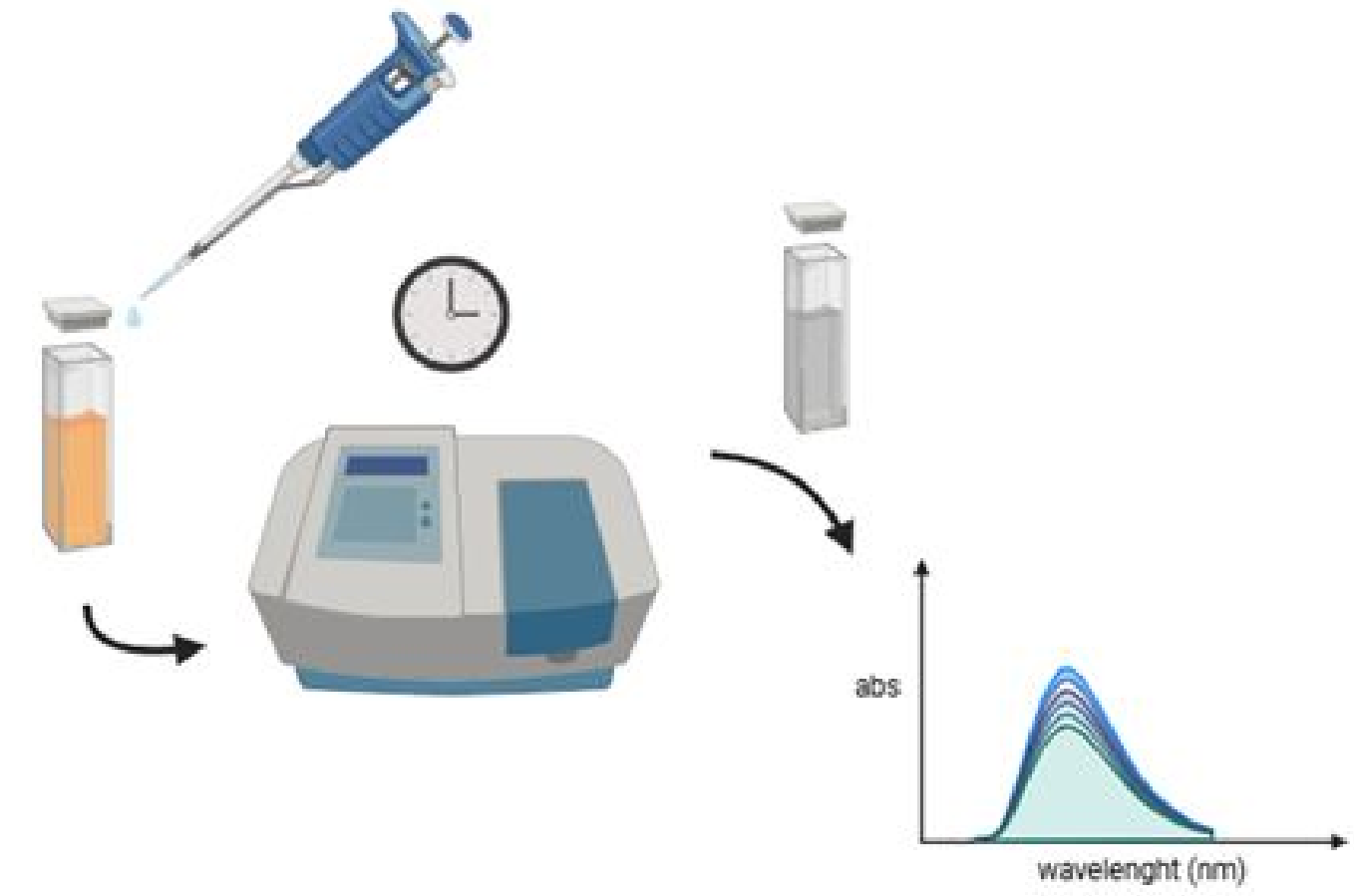


**Figure S30.** Experimental setup used for following the degradation of morin and Sunset Yellow FCF.
